# What Are We Looking for in Computer-Based Learning Interventions in Medical Education? A Systematic Review

**DOI:** 10.2196/jmir.5461

**Published:** 2016-08-01

**Authors:** Tiago Taveira-Gomes, Patrícia Ferreira, Isabel Taveira-Gomes, Milton Severo, Maria Amélia Ferreira

**Affiliations:** ^1^ Department of Medical Education and Simulation Faculty of Medicine University of Porto Porto Portugal; ^2^ Department of Clinical Neurosciences and Mental Health, Medical Psychology Unit Faculty of Medicine University of Porto Porto Portugal; ^3^ Department of Clinical Epidemiology, Predictive Medicine and Public Health Faculty of Medicine University of Porto Porto Portugal

**Keywords:** medical education, internet-based learning, computer-based learning, e-learning, b-learning, systematic review

## Abstract

**Background:**

Computer-based learning (CBL) has been widely used in medical education, and reports regarding its usage and effectiveness have ranged broadly. Most work has been done on the effectiveness of CBL approaches versus traditional methods, and little has been done on the comparative effects of CBL versus CBL methodologies. These findings urged other authors to recommend such studies in hopes of improving knowledge about which CBL methods work best in which settings.

**Objective:**

In this systematic review, we aimed to characterize recent studies of the development of software platforms and interventions in medical education, search for common points among studies, and assess whether recommendations for CBL research are being taken into consideration.

**Methods:**

We conducted a systematic review of the literature published from 2003 through 2013. We included studies written in English, specifically in medical education, regarding either the development of instructional software or interventions using instructional software, during training or practice, that reported learner attitudes, satisfaction, knowledge, skills, or software usage. We conducted 2 latent class analyses to group articles according to platform features and intervention characteristics. In addition, we analyzed references and citations for abstracted articles.

**Results:**

We analyzed 251 articles. The number of publications rose over time, and they encompassed most medical disciplines, learning settings, and training levels, totaling 25 different platforms specifically for medical education. We uncovered 4 latent classes for educational software, characteristically making use of multimedia (115/251, 45.8%), text (64/251, 25.5%), Web conferencing (54/251, 21.5%), and instructional design principles (18/251, 7.2%). We found 3 classes for intervention outcomes: knowledge and attitudes (175/212, 82.6%), knowledge, attitudes, and skills (11.8%), and online activity (12/212, 5.7%). About a quarter of the articles (58/227, 25.6%) did not hold references or citations in common with other articles. The number of common references and citations increased in articles reporting instructional design principles (*P*=.03), articles measuring online activities (*P*=.01), and articles citing a review by Cook and colleagues on CBL (*P*=.04). There was an association between number of citations and studies comparing CBL versus CBL, independent of publication date (*P*=.02).

**Conclusions:**

Studies in this field vary highly, and a high number of software systems are being developed. It seems that past recommendations regarding CBL interventions are being taken into consideration. A move into a more student-centered model, a focus on implementing reusable software platforms for specific learning contexts, and the analysis of online activity to track and predict outcomes are relevant areas for future research in this field.

## Introduction

Medical education is a field that reflects the constant revision of medical knowledge, educational technology, and teaching strategies. For over a century, education in general [1] and medical education in particular [2-4] have been shifting from the traditional instructor-centered model to a learner-centered model, a shift in which the learner has greater control over the learning methodology and the teacher becomes a facilitator of the learning process [5]. This transition was required, since advances in medical knowledge and changes in health care delivery have weighed on the teaching responsibilities of medical schools [6]. The need to review and incorporate emerging fields into the curricula required medical schools to look for means to deliver education with less reliance on instructor availability [6]. The broadening of the setting in which health care is delivered—from the hospital to the community setting—prompted adaptation of these venues to ensure education could be delivered remotely [7]. Digital technology enabled the development of computer-based learning (CBL) and, later, Web-based learning methodologies, which allowed medical schools to cope with the pressing changes in the medical education landscape [4].

The increasing interest in and pervasiveness of CBL and Web-based learning was accompanied by research on how such methods compared with traditional instruction on a wide spectrum of educational end points, leading Friedman in 1994 to reflect on the research we should be doing on CBL [8]. In 2000, Adler and Johnson quantified the medical literature on CBL, concluding that researchers should focus on determining in which settings CBL methods are most adequate, rather than comparing them with the classroom setting [9]. According to these authors, provided that CBL offers tools that cannot be replicated by other means, the typical classroom setting cannot be considered a sound comparison group, as it undermines study internal validity [9,10].

The apparent lack of accommodation of this recommendation in subsequent studies, which kept growing in variety of setting and design, led Cook in 2005 to establish an agenda for research in medical education, suggesting once again that CBL research should look at relative benefits between different CBL methods [11]. In 2008, Cook et al conducted a broad meta-analysis of the effects of CBL in health sciences education, showing that CBL interventions are generally better than no intervention and marginally superior to traditional instruction [12]. Studies using multimedia learning content and student feedback reported the best results [12].

While the issue around CBL arose nearly 22 years ago, and over 8 years have passed since the Cook et al meta-analysis, comparative research between CBL methods is still a contemporary problem [13]. It is relevant to study what features of educational software researchers are reporting, how interventions are being conducted, what end points are being measured, and whether prior recommendations are informing current research. To our knowledge, since 2008 this issue has not been looked at again in a broad and systematic way, and is yet to be carried out specifically in medical education, as opposed to health sciences education in general.

Thus, this work aimed to identify reports of CBL software and CBL interventions, specifically in medical education, and systematically describe features of educational software, instructional design considerations, and the design, setting, and end points of CBL interventions. Finally, we intended to summarize these findings through determining subgroups of similar articles about educational software features and intervention end points, and to understand the extent to which prior work is being taken into consideration by analyzing the reference and citation network of these publications.

## Methods

### Study Eligibility

We included medical education studies written in English regarding the development of educational software, interventions using educational software, or both. We considered interventions during training or clinical practice that reported effects on learner attitudes, knowledge, and skills, as well as records of online activity. We included pretest-posttest studies, randomized and nonrandomized studies, parallel group and crossover studies, and studies in which a software-based intervention was added to other instructional methods [12].

We did not include studies that exclusively surveyed perceptions and attitudes of students or professionals toward CBL in general, nor studies that solely described course structure or reported how CBL strategies were implemented in medical schools.

### Study Identification

We designed a strategy to search MEDLINE, Scopus, Web of Science, and EBSCO databases. Search terms were “medical education,” “medical students,” “e-learning,” “blended learning,” “information technology,” “instructional design,” “software,” and “Web-based platform,” among other terms. The exact queries are available in Multimedia Appendix 1. We established an 11-year period from January 1, 2003 to December 31, 2013. We performed the final database search on January 5, 2015.

### Study Selection

Working independently and in duplicate, reviewers (PF, ITG) screened all article titles and abstracts, and in full text all potentially eligible abstracts, abstracts with disagreement, or abstracts with insufficient information. Independently and in duplicate the reviewers considered the eligibility of studies in full text with adequate chance-adjusted interrater agreement of .92 by intraclass correlation (ICC) using the psych package, version 1.5.1 [14] for the R programming language.

### Study Analysis

#### Data Extraction

We conducted data extraction and reporting in accordance with the Preferred Reporting Items for Systematic Reviews and Meta-Analyses (PRISMA) guidelines [15,16]. Reviewers abstracted data from each eligible study using a standardized data abstraction spreadsheet. We developed, tested, and revised the spreadsheet based on the review results of the first 30 assessed articles. Conflicts were resolved by consensus with a third reviewer (TTG). We abstracted information on publication year, country, study design, software used, instruction delivery method, CBL interactive features, CBL sharing features, instructional design principles, participant number and training level, study duration, type of comparison between groups, instruments used for assessment of knowledge, attitudes, and skills, correlations between study end points, and records of student online activity. For all categories, information was based on an explicit report of the variables of interest, except for instructional design principles, which we inferred from descriptions and figures using standardized criteria, whenever there were no explicit references [17]. In addition, articles that reported interventions were graded using the Medical Education Research Study Quality Index (MERSQI) for article reporting quality in medical education [18,19].

#### Data Analysis

We manipulated and prepared data for statistical analysis using NumPy [20] and pandas [21] libraries for the Python language. Latent class analysis uncovered distinct homogeneous groups of articles from the study population, considering that the performance of each article in a set of articles is explained by a categorical latent variable with *k* classes, commonly called latent classes [22]. Interpretation of the model was based on article profiles for each category, obtained from the probability of observing each variable in each class. We defined the number of latent classes according to the Bayesian information criterion (BIC), which is a measurement of model fit that penalizes models with many parameters, preventing model overfit [22]. Starting from a model with 1 class and increasing 1 class at a time, we chose the best model as the one with best interpretability and lowest BIC [22]. We created 2 latent class models, one taking into consideration educational software variables, and the other taking into consideration intervention end point variables. We did not use variables reported in <2% of the studies to compute the classes. Statistical analysis was conducted using the R programming language (The R Foundation). Class models were fitted using the poLCA package [23]. Summary panels were created using the ggplot2 package [24].

### Reference and Citation Analysis

#### Data Extraction

We obtained references of the included papers from Scopus using digital object identifiers (DOIs). We obtained citations of the included papers from Google Scholar by searching for each of the articles by title and abstracting the papers on the “cited by” link. This procedure was carried out using a script built with the WebDriver library [25] for the JavaScript programming language. In order to uniquely identify every reference and citation, we performed a duplicate match and removal procedure by looking for similar matches of the title and authors’ names using the fuzzywuzzy library [26] for the Python programming language. We considered 2 references or citations to be the same when the matching probability was >85%. Matching probability was computed using the Levenshtein string distance [27].

#### Data Analysis

We analyzed the distribution of the total number of references and citations for each paper, and grouped papers based on whether they had ≥1 references or citations in common. We looked for the relationship between the number of citations and interventions comparing traditional instruction versus CBL methods, or CBL versus CBL. In addition, we assessed whether the number of related papers was associated with educational software latent classes or intervention end point latent classes, and with specific references to reviews by Cook and colleagues on CBL [11,12,28]. We used linear models adjusted for article publication year for this purpose. Statistical analysis was performed using the R language. We analyzed the article network using the graph-tool library for the Python programming language [29]. Error plots were created using the ggplot2 package [24] the R programming language.

## Results

### Study Eligibility, Identification, and Selection

**Figure 1:**
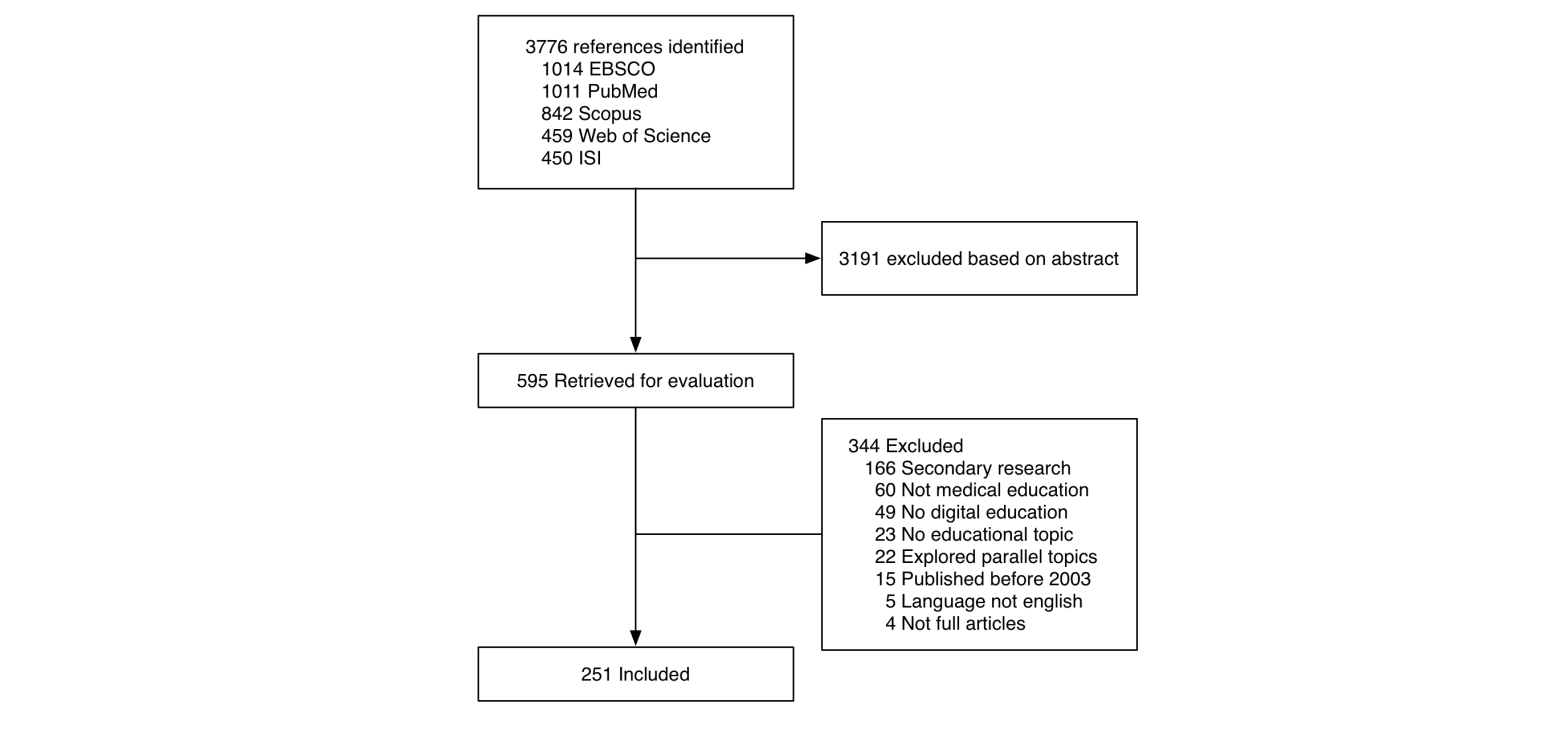
Flow of a systematic review of the literature published January 1, 2003 to December 31, 2013 regarding either the development of instructional software or interventions using instructional software.

The search strategy yielded 3776 citations, of which we identified 595 potentially eligible articles based on their abstract. Of these, we excluded 344 articles based on a full-text review. In total, we included and analyzed 251 articles. Overall mean ICC was .98. Specific ICCs are reported for variables that were not always explicitly present and relied on reviewer judgment, or when <.95. Figure 1 shows details regarding the study flow.

### Study Analysis

**Figure 2:**
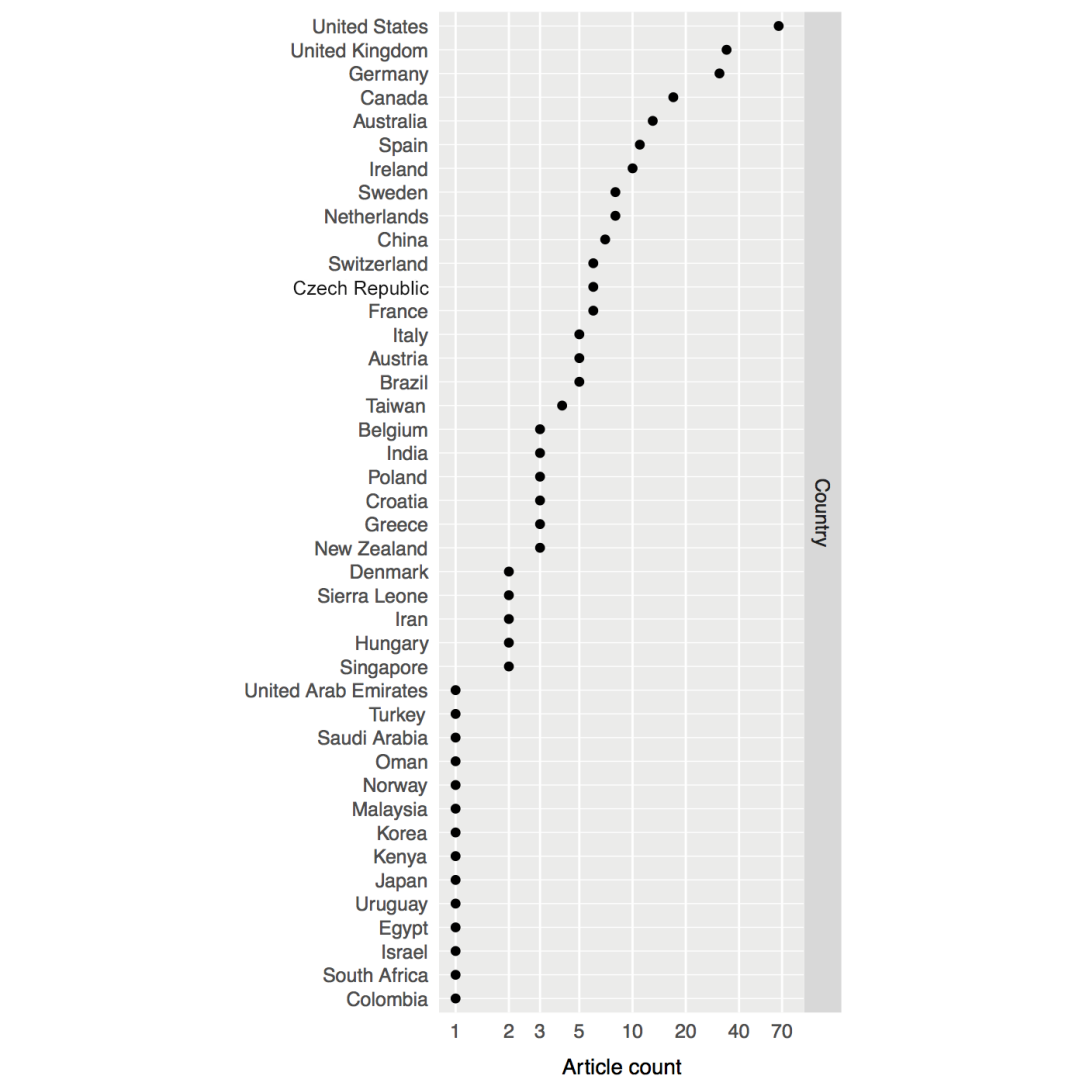
Articles published per country of medical school. The article count axis is presented in logarithmic scale for better data representation.

The number of publications rose over the years, from 13 of the 251 publications in 2003–2004 (5.2%), to 82 in 2012–2013 (32.7%). Medical schools in Germany, the United Kingdom, and the United States contributed more than 30 papers each between 2003 and 2013. Medical schools in Australia, Canada, and Spain contributed more than 10 papers each. Figure 2 presents contributions per medical school nationality. A total of 38 different software platforms were reported, which are listed in Multimedia Appendix 2. Of these, 13 were general educational platforms (34%), the most frequently used being Moodle [30-37] and Blackboard [38-46], mentioned in 8 papers, and WebCT [47-52], mentioned in 6 papers. The online virtual world Second Life [53,54] was mentioned in 2 papers, and 9 additional platforms were mentioned once. Of the 38 platforms, 25 were developed specifically for medical education (66%). Of these platforms, 4 were virtual patient simulators that were mentioned in 3 papers each: CASUS [55-58], HINTS [59-61], INMEDEA [62-64], and Web-SP [34,65,66]. One learning management system named MEFANET [67,68] was mentioned in 2 papers. Finally, 20 other platforms were mentioned once. These platforms were either learning management systems or virtual patient simulators. Of these, 4 systems were specialized in medical fields: a serious 3D game named EMSAVE [69], a system for learning electrocardiography named EKGtolkning [70], a platform entitled Radiology Teacher [71], and a virtual microscope named MyMiCROscope [72].

**Figure 3:**
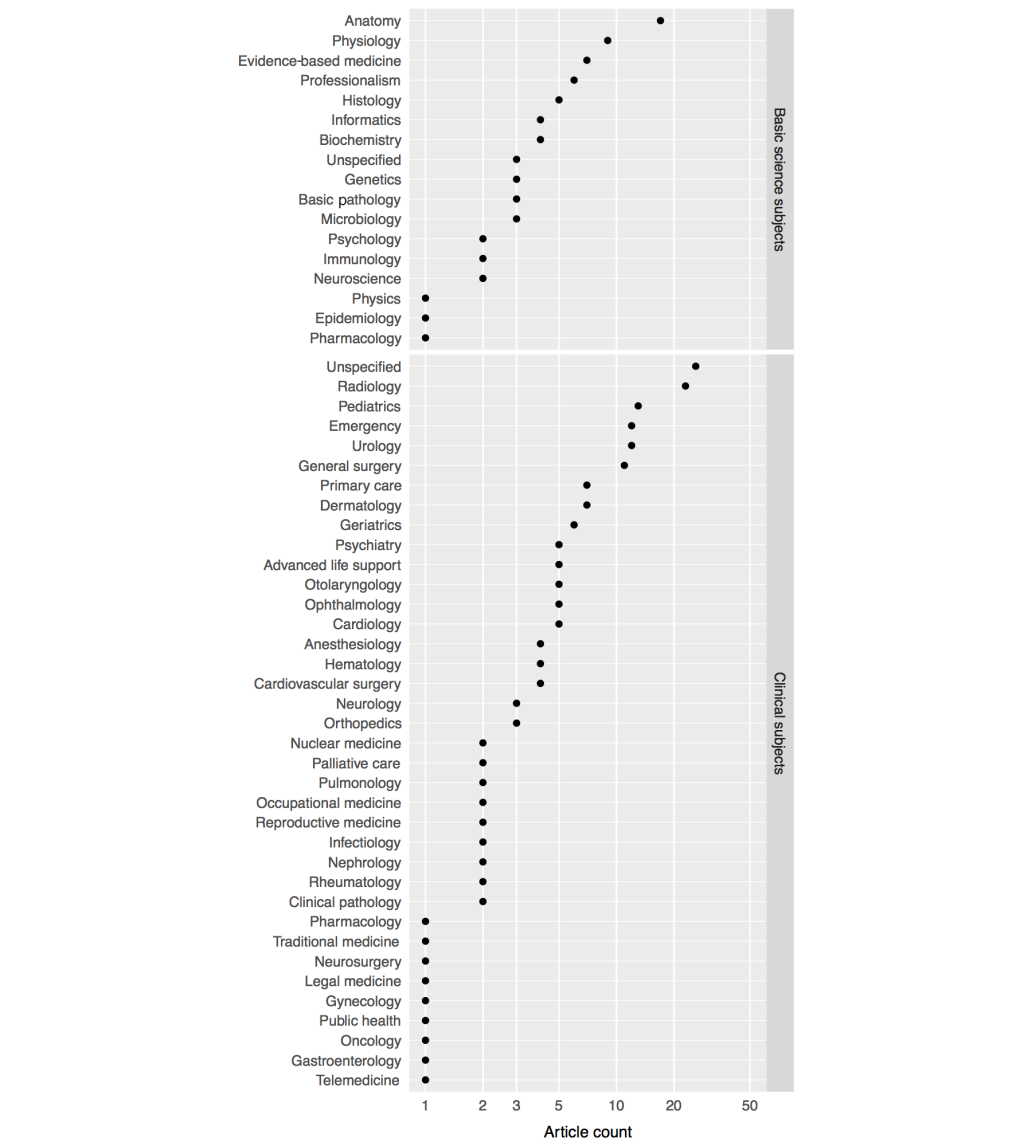
Articles per basic science and clinical subject. The article count axis is presented in logarithmic scale for better data representation.

A total of 146 studies were conducted on clinical specialties (58.2%), 70 studies on basic sciences (27.9%), and 36 studies on surgical specialties (14.3%). Radiology was the clinical specialty with most studies, in 23 articles (9.2%), followed by pediatrics with 13 (5.2%). The basic science subjects with most publications were anatomy with 18 articles (7.2%) and physiology with 9 articles (3.6%). The most studied surgical specialties were urology with 12 studies (4.8%) and general surgery with 10 (4.0%). There was at least one article in most basic sciences and medical specialties, as Figure 3 shows.

**Figure 4:**
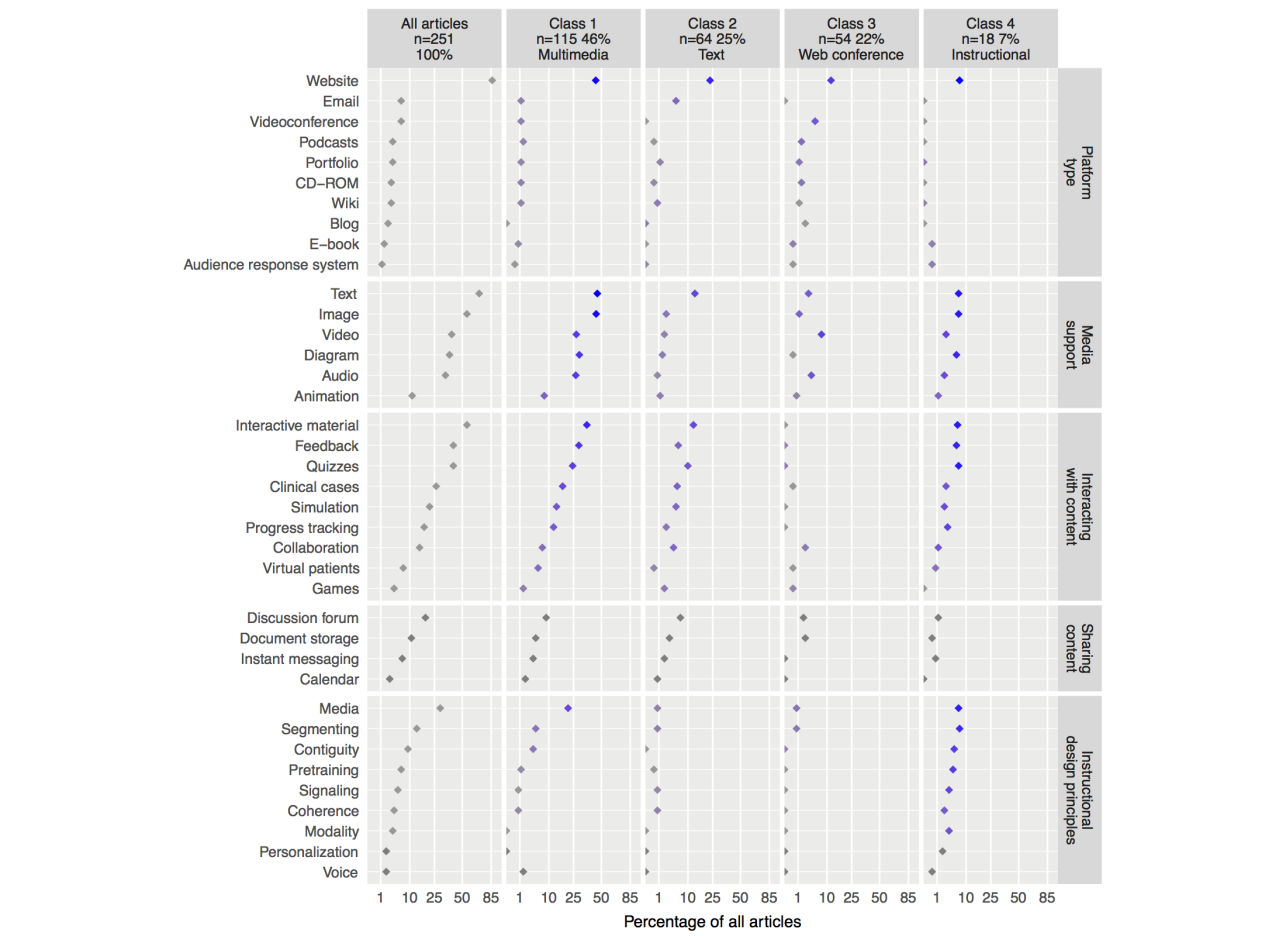
Prevalence of articles per educational software feature and educational software latent class. Horizontal axis ranges between 0 and 100 on a squared root scale. Point color specifies the probability of assigning a paper to each class based on the presence of each variable (gray indicates a probability of 0, ranging to dark blue indicating the highest probability). From the listed variables, those present in more than 2% of all articles were used to determine the educational software latent classes.

### Web-Based Learning Software

Of the 251 studies assessed, 113 reported blended learning environments (45.0%, ICC=.98) and 138 reported e-learning environments (55.0%, ICC=.99). Figure 4 summarizes the results for this section and depicts the percentage of studies and relative contribution of each of the learning software variables to the software latent classes described below.

#### Platform Type

A total of 217 studies used websites (86.5%), 16 used videoconference (6.4%), and 16 other studies used email (6.4%); 9 used podcasts (3.6%) and 9 used portfolios (3.6%). Wikis (3.2%, ICC=.90) and CDs (3.2%, ICC=.83) were both reported in 8 studies. Blogs were reported in 6 studies (2.4%). E-books were reported in 4 studies (1.6%) and audience response systems in 3 articles (1.2%).

#### Media Support

Of the 251 studies, 174 provided content in text format (69.3%), and 138 used images (55.0%). Video was reported in 99 studies (39.4%) and diagrams in 94 studies (37.5%). Audio was used in 85 articles (33.9%), and animations were reported in 28 articles (11.2%).

#### Interacting With Content

A total of 138 studies reported unspecified features (55.0%). The software provided feedback to the learner in 103 studies (41.0%); 103 articles reported quizzes (41.0%), 66 reported clinical cases (26.3%), 54 described simulations (21.5%), and 45 tracked learner performance (17.9%). Features allowing collaboration between learners and instructors were reported in 38 studies (15.1%). Virtual patients were reported in 18 studies (7.2%) and games were described in 10 studies (4.0%).

#### Sharing Content

Of the 251 studies, 47 reported communication and content sharing through discussion forums (18.7%), 27 reported the ability to store documents (10.8%), and 7 used instant messaging communication systems (2.8%). Calendar features were also reported in 7 studies (2.8%).

#### Instructional Design Principles

The media principle was apparent in 74 studies (29.5%, ICC=.94), followed by the segmenting principle in 34 studies (13.6%, ICC=.98) and the contiguity principle in 23 studies (9.2%, ICC=1.00). The pretraining principle was identified in 16 studies (6.4%, ICC=.98) and the signaling principle in 13 studies (5.2%, ICC=.97). The coherence principle was identified in 10 studies (4.0%, ICC=.97) and the modality principle in 9 studies (3.6%, ICC=1.00). Finally, the personalization principle and the voice principle were identified in 5 studies each (2.0%, ICC=1.00).

#### Latent Classes

We considered 4 distinct classes for educational software, according to the model statistics in Table 1. Class 1 was composed of 115 studies (45.8%), mostly of website-based interactive systems presenting content using text, images, audio, and video. Student feedback features were frequently described, namely quizzes and clinical cases. Aside from the multimedia principle, instructional design considerations were rarely present. We thus labeled class 1 *multimedia*.

**Table 1:** Latent class analysis model fit per number of classes for educational software.

No. of classes	Log likelihood	Parameter number	BIC^a^
1	–2340	21	4797
2	–2017	43	4273
3	–1923	65	4207
4^b^	–1866	87	4214^b^
5	–1854	109	4230

^a^BIC: Bayesian information criterion.

^b^The number of classes selected for the educational software model. This decision was based on picking the model with the best interpretability and lowest BIC.

Class 2 was composed of 64 studies (25.5%) using websites, and to a smaller extent email, to deliver instructional content mostly in the form of text. Interactive features were less frequent than in class 1, and instructional design considerations were scarce. We thus labeled class 2 *text*.

Class 3 was composed of 54 studies (21.5%) making use of websites and videoconference platforms to provide video and audio content. Interactivity and instructional design principles were nearly nonexistent. We thus labeled class 3 *Web conference*.

Class 4 contained 18 studies (7.2%) mostly using Web-based interactive multimedia apps in which the use of multiple instructional principles was frequent. We thus labeled class 4 *instructional*.

The four right-hand columns in Figure 4 depict the composition of each class and the relative weight of each variable on class assignment.

### Interventions

**Figure 5:**
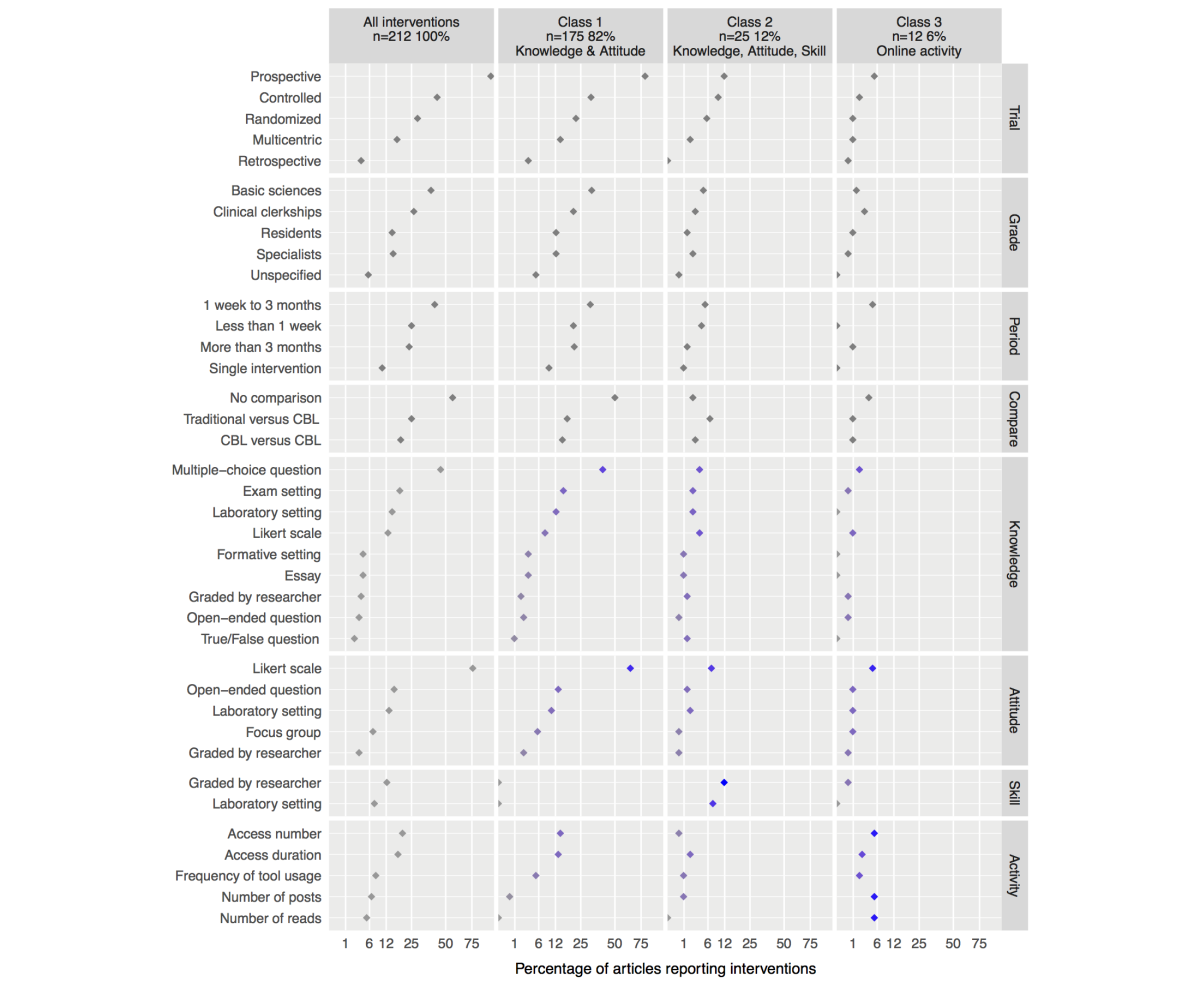
Prevalence of articles per intervention feature and intervention endpoint latent class. Horizontal axis ranges between 0 and 100 on a squared root scale. Point color specifies the probability of assigning a paper to each class based on the presence of each variable (gray indicates a probability of 0, ranging to dark blue indicating the highest probability). Only variables regarding assessment of knowledge, attitudes, skills, and online activity (the 4 last panels) were used to determine intervention end point latent classes. CBL: computer-based learning.

Of the 251 articles included in this study, we identified 212 conducting interventions on the end points of interest (84.5%). Figure 5 summarizes the results for this section and depicts the percentage of studies for each intervention characteristic, and the relative contribution of intervention end point variables to the *intervention end point* latent class described below.

#### Study Design and Study Sample

A total of 81 of 212 studies were conducted with medical students from preclinical years (38.2%) and 56 studies involved students during clinical rotations (26.4%). In addition, 32 studies were conducted with specialist medical doctors (15.1%), and 31 studies were conducted with medical residents (14.6%).

In total, 55 interventions were carried out with <50 participants (25.9%), 97 studies had a sample size ranging between 50 and 200 participants (45.8%), and 59 studies were conducted with >200 students (27.8%).

Of the 212 studies, 54 were conducted over <1 week (25.5%), 90 articles reported interventions lasting between 1 week and 3 months (42.5%), and 50 studies were conducted for >3 months (23.6%).

In addition, 84 studies repeatedly tested participants in a pre-post approach (39.6%), and 93 made use of control groups (43.9%). A total of 61 studies were randomized (28.8%) and 37 studies had participants from more than one institution (17.5%); 40 studies compared different CBL approaches (18.9%), while 53 studies compared CBL with traditional methods (25.0%).

The mean MERSQI score for the assessed studies was 9.54 (SD 1.84).

#### Conducted Comparisons Between Groups

Of the 212 studies, 28 studied controlled interventions between blended learning approaches and traditional lectures (13.2%), while 11 studies compared e-learning approaches with traditional lectures (5.2%). A total of 8 studies compared spaced repetition versus bolus learning (3.8%), and 7 studies compared e-learning *v* ersus no intervention (3.3%). In addition, 5 studies compared the use of 3D models versus 2D images (2.4%). A multitude of other comparisons were performed, such as exploratory versus blocked learning approaches [73-75], complex versus simple user interfaces [73,76,77], immediate versus delayed completion of lectures in CBL systems [78], and multimedia versus text on CBL media [73,79-81]. Multimedia Appendix 3 lists the different comparison groups we identified for each of the 212 articles reporting interventions.

#### Knowledge End Point

Knowledge outcomes were assessed in 120 of 212 articles (56.6%). Objective knowledge was assessed using multiple choice quizzes in 98 of 120 studies (81.7%), 9 articles used free-text fields (7.5%), and 8 articles used open-ended questions (6.7%, ICC=.89). In addition, 5 studies used true/false questions (4.2%). Judgments of knowledge were collected using Likert scales in 27 studies (22.5%). Researchers directly assessed knowledge in 9 studies (7.5%). A total of 31 studies were conducted in a laboratory setting (25.8%). Knowledge assessment was part of a final examination in 39 articles (32.5%), and in 9 studies assessment was part of a formative assessment (7.5%). Of the 120 studies, 90 reported that interventions improved knowledge acquisition (75.0%), while 27 studies did not find significant effects (22.5%) and 3 multicenter randomized controlled trials reported that interventions did not positively affect knowledge acquisition (2.5%) [66,82,83].

#### Attitude End Point

Of 212 studies, 172 assessed student attitudes (81.1%); of these, 163 used Likert scales (94.8%) and 34 used free-text fields (19.8%). In 8 articles researchers assessed participants’ attitudes directly (4.7%). A total of 29 studies were conducted in a laboratory setting (16.9%) and 16 studies made use of focus groups (9.3%). In addition, 161 studies found positive attitudes toward interventions (93.6%), 8 found neutral attitudes (4.7%), and 3 reported negative attitudes (1.7%) [84-86].

#### Skill End Point

Of 212 studies, 31 assessed subject skills (14.6%). In 26 of these studies, skills were assessed directly by researchers (84%) and in 16 studies assessment was conducted in a laboratory setting (52%). In addition, 24 studies found positive effects on skills acquisition (77%), 5 reported that the interventions had no effect on assessed skills (16%), and 2 reported that the intervention had negative effects (6%) [82,86].

#### Online Activity End Point

Online activity was measured in 76 of 212 studies (35.9%). Of these studies, 46 measured total logins to the system (60%), 39 measured time spent in the system (51%), and 18 measured the number of times students used specific learning tools (24%). Further, 16 studies measured the number of student posts (21%), and 12 measured the number of times students viewed the learning materials (16%). A total of 41 studies found no relationship between activity patterns and learning outcomes (54%), 34 articles reported increased activity to have positive effects on learning outcomes (45%), and 1 study found a negative effect (1%) [66].

#### Intervention End-Point Latent Classes

We considered 3 distinct classes to group the 212 studies taking into consideration intervention end point variables. Class 1 contained 175 articles assessing knowledge and attitudes (82.5%). We labeled class 1 *knowledge and attitude*. Class 2 contained 25 intervention studies (11.8%). In addition to assessing knowledge and attitudes, articles in this class also assessed skills. We labeled class 2 *knowledge, attitude, and skill*. Class 3 contained 12 studies that assessed online activity, specifically through the number of posts and number of reads (5.7%). Attitudes were always assessed, but knowledge and skill assessment were nearly absent. We labeled class 3 *online activity*. Table 2 reports model statistics for the intervention end point latent classes, and Figure 5 depicts the prevalence of articles per intervention feature and intervention end point latent class.

**Table 2:** Latent class analysis model fit per number of latent classes for intervention end points.

No. of classes	Log likelihood	Parameter number	BIC^a^
1	–1631	22	3382
2	–1510	45	3265
3^b^	–1451	68	3270^b^
4	–1424	91	3268

^a^BIC: Bayesian information criterion.

^b^The number of classes for the intervention end point model. This decision was based on picking the model with the best interpretability and the lowest BIC.

#### Reported Correlations Between Assessment Outcomes

Of 212 studies, 25 correlated different variables with knowledge outcomes (11.8%). Of these, 1 study correlated system interactivity with knowledge scores and concluded that lower levels of interactivity benefitted knowledge acquisition [73]. Correlations between knowledge gains and time spent using online platforms were also sought. These were found to be positive in 4 studies [49,87-89] and neutral in 1 study [76]. In addition, 1 study described a modest positive correlation between increased knowledge scores on the learning system and an increase in examination scores [90]. Increased learning platform usage was correlated positively with knowledge acquisition in 5 studies [90-94], while 4 found no association [46,95-97]. Other studies found positive relationships between knowledge and the number of posts in online forums [98,99] and comprehensiveness of student study materials [100]. Regarding attitudes, 2 articles found a mild positive correlation between judgments of knowledge and knowledge score [101,102]. Other correlations were assessed, namely confidence and skill [103], study duration and skill [104], and study duration and learning style [105], but did not reach statistical significance.

### Reference and Citation Network Analysis

#### Reference and Citation Analysis

We obtained references and citations for 227 of the 251 articles included in this review (90.4%). The mean number of references was 26.12 (SD 17.41). In total, the abstracted articles had 4010 references to other articles. The most referenced articles were from Ruiz et al [4], Cook et al [12], Chumley-Jones et al [106], Greenhalgh [107], Ward et al [108], Muller [109], and Ellaway and Masters [110]. The mean number of article citations of the 227 abstracted articles was 14.43 (SD 12.12). More than half of the references were common to various abstracted articles, while a smaller percentage of studies had independent sets of references.

#### Related Article Analysis

**Figure 6:**
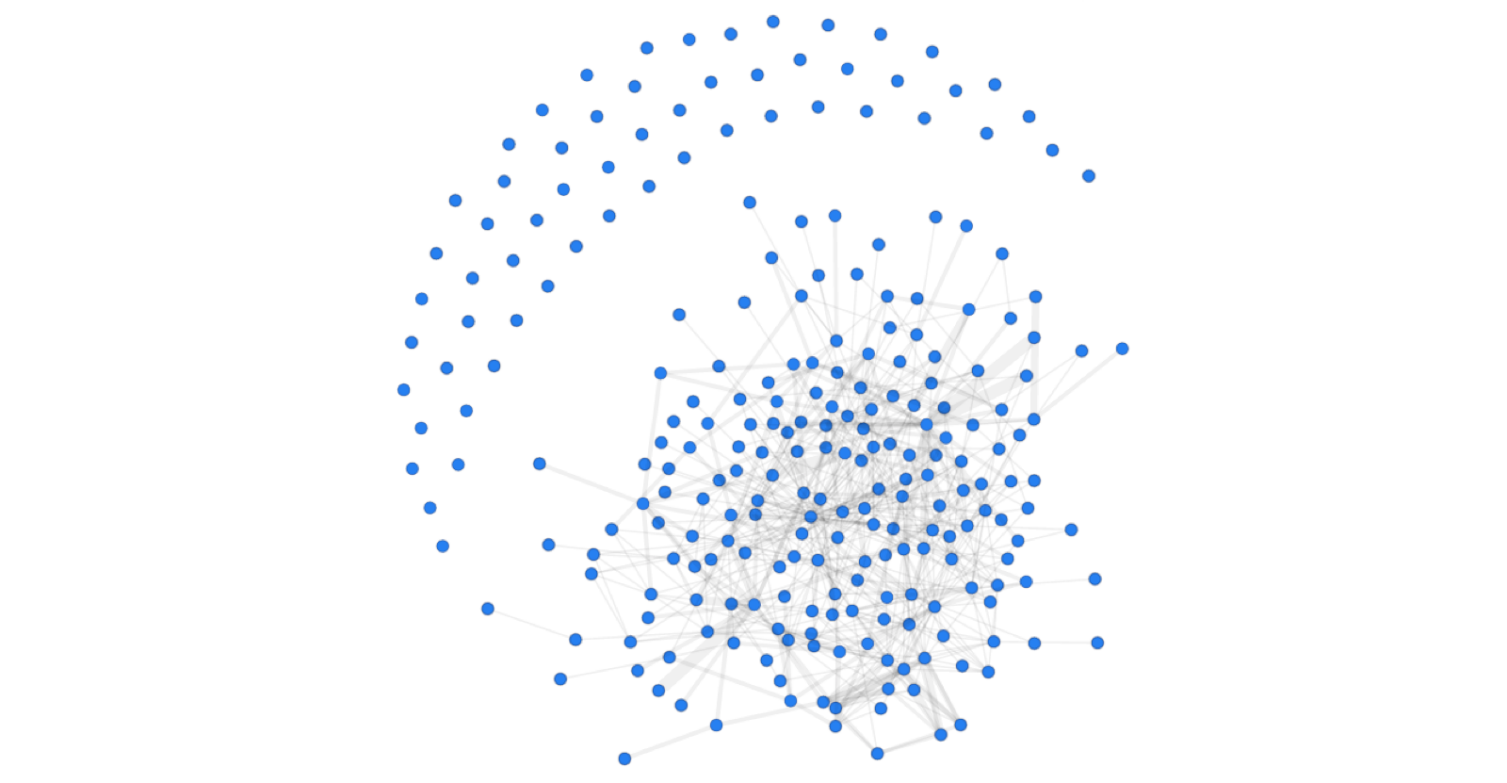
Relationships between articles included in this review (indicated by nodes). Links between nodes indicate that articles have references and citations in common. The width of the link indicates the number of studies in common, ranging from 1 to 5. About a quarter of the studies have no common references or citations. Only 227 of the 251 studies were included in this analysis due to missing information (90.4%).

Of the 227 articles, 169 had at least one reference or citation in common with other abstracted articles (74.4%), and were thus said to be related, as depicted in Figure 6. A total of 58 articles were not related to any other article, since they did not share references or citations (25.6%). The mean number of related studies for each article included in this review was 4.74 (SD 5.42).

#### Citation Differences Between Intervention Group Types

**Figure 7:**
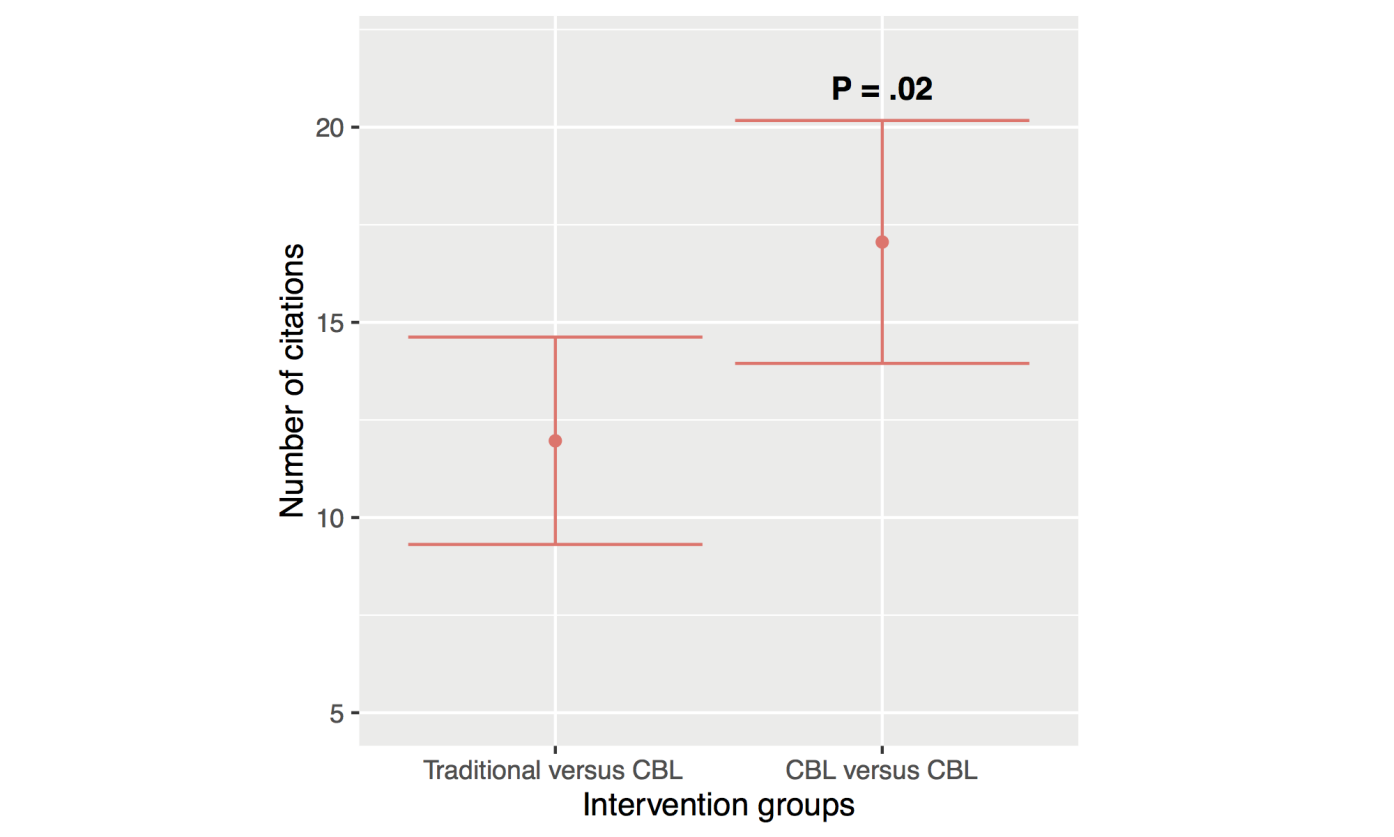
Mean citation number differences between traditional versus computer-based learning (CBL), and CBL versus CBL, adjusted for publication date. For CBL versus CBL, only 227 of the 251 studies were included in this analysis due to missing information (90.4%). Error bars represent the 95% CI.

Studies comparing traditional versus CBL methods were cited a mean of 11.92 times (95% CI 9.31–14.6). Studies comparing different CBL methods were cited a mean of 16.71 times, which was statistically significant (95% CI 13.95–20.17, *P*=.02). Figure 7 shows this result.

#### Associations With Latent Classes and the Cook et al Review

Regarding educational software latent classes, articles in the multimedia class had a mean of 3.95 related studies (95% CI 2.99–4.91), while the text class had a mean of 4.98 (95% CI 3.69–6.26, *P*=.19). Articles from the Web conference class had a mean of 5.02 relationships to other studies (95% CI 3.64–6.45, *P*=.22) and articles in the instructional class had a statistically significant mean of 6.78 studies (95% CI 4.37–9.20, *P*=.03) in common. Regarding the intervention end point latent classes, articles in the knowledge and attitude class had a mean of 2.63 related studies (95% CI 1.46–3.80) and the knowledge, attitude, and skill class had a mean of 2.88 studies in common, reaching statistically significance versus the knowledge and attitude class (95% CI 0.71–5.04, *P*=.04). Articles from the online activity class had a mean of 6.78 related studies (95% CI 3.60–9.96, *P*=.01), also reaching a significant value when compared with the knowledge and attitude class.

**Figure 8:**
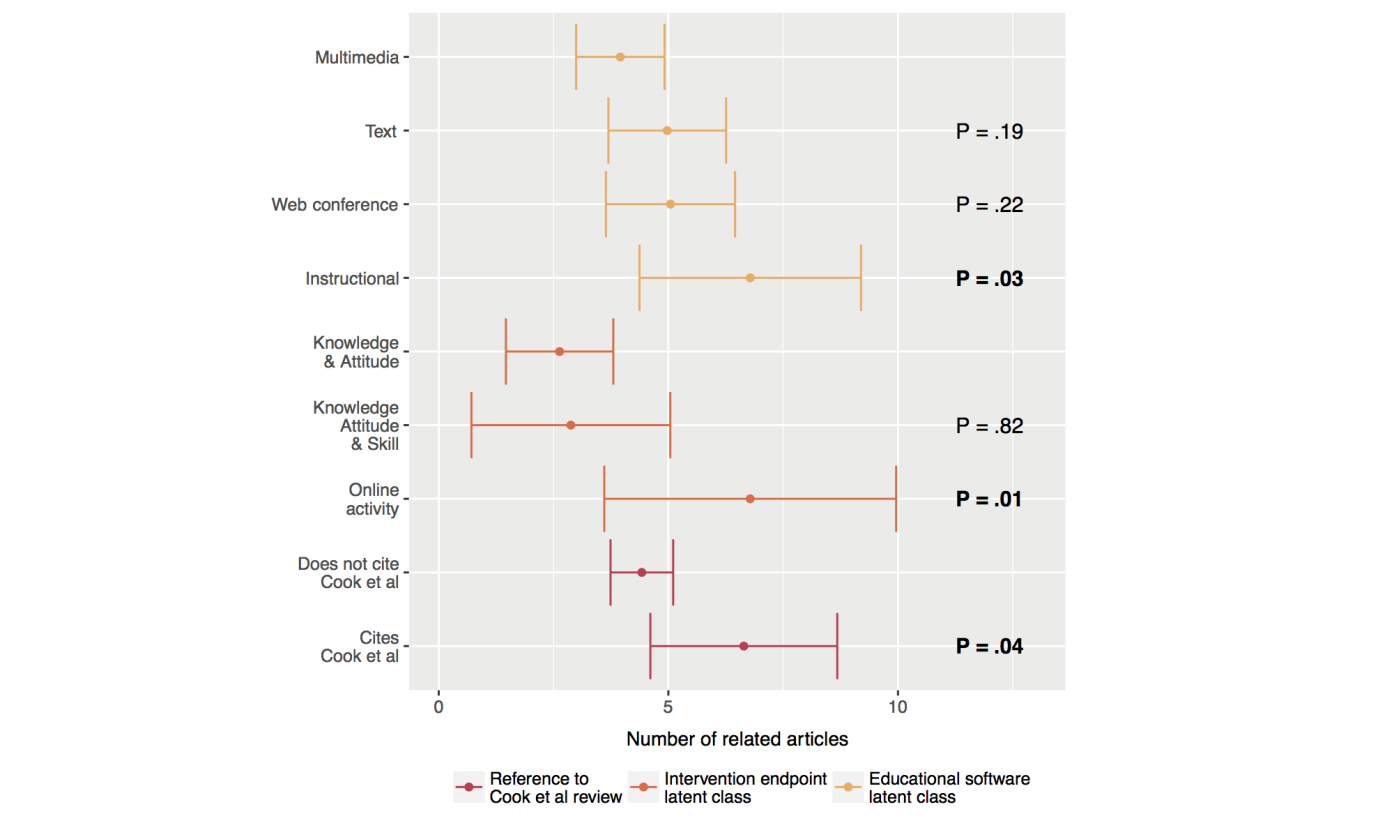
Mean number of related articles per latent class and reference to the Cook et al review. Number of related articles is adjusted for publication date. *P* values indicate intraclass pairwise differences from the topmost element of each color-coded class. Significant relationships are marked in bold typeface. Only 227 of the 251 studies were included in this analysis due to missing information (90.4%). Error bars represent the 95% CI.

Finally, articles not citing the Cook et al work had a mean related article count of 4.42 (95% CI 3.74–5.11), while articles citing Cook et al had a mean count of 6.64 (95% CI 4.61–8.68, *P*=.04), which was significantly different. Figure 8 plots the complete results for this section.

## Discussion

The number of articles on CBL in medical education has been rising, with reports of over 38 different software systems, 25 of which were specifically developed for medical education (66%). Of the 251 studies we analyzed, most used interactive websites making use of text and images (46%) and, to a smaller extent, websites delivering text-based materials (25%). A similar number of reports delivered instruction using Web conferencing systems (22%), and a smaller group of studies reported highly interactive websites with multimedia learning content built according to instructional design principles (7%). Of the 212 interventions, most did not use comparison groups and lasted between 1 week and 3 months. CBL versus CBL studies were less numerous than traditional versus CBL studies. Nearly all studies assessed student attitudes, of which a large fraction also assessed knowledge (82%), and a smaller fraction assessed knowledge and skills (12%). A smaller set of studies looked specifically for patterns of online activity, namely the number of reads and posts (6%). Finally, nearly 75% of articles had references and citations in common, while 25% of the analyzed articles did not have any references in common. Articles comparing different CBL methods were cited more often than were studies comparing traditional versus CBL methods, independent of publication date. Articles reporting instructional design principles, articles measuring online activity, and articles citing the Cook et al CBL reviews had significantly more references and citations in common than did other articles.

### Comparison With Previous Reviews

The last systematic review and meta-analysis of this topic encompassed data from 1990 to 2006 and highlighted the problems of intervention variability and lack of evidence for comparative effects of CBL methods [12,13,28]. Recent reviews have also demonstrated that practice exercises, interactivity, feedback, and repetition can favorably influence learning outcomes [13,49]. Other reviews summarized technologies and methods used [111,112], and addressed specific topics such as the role of blogs [113], wikis [114], portfolios [115], simulations in general [116] and for surgery in particular [117], gastroenterology [118], catheterization [119], and airway management [120]. Other authors focused on specific aspects of the effects of Web-based learning on problem-based learning [121], and the implications of recent Web capabilities, namely Web 2.0 [122,123] and Web 3.0 [124], for medical education. Our study complements previous reviews by encompassing recent work concerning these fields over a large base of abstracted articles. Despite the considerable time overlap with similar reviews, assessments such as latent class analysis and citation network analysis had not yet been conducted during the considered time period [13].

### Limitations and Strengths

This study has limitations. We scrutinized databases that frequently index medical education articles. Although we did not query EMBASE, Scopus covers most of the literature indexed in EMBASE and thus Scopus provided a reasonable proxy. However, we did not abstract the gray literature or references from other articles, and thus our article search cannot be considered exhaustive. We narrowed the study participants to medical education only. This can be considered a limitation insofar as these findings cannot be generalized to other health professions. Other reviews have performed similar searches including articles in health professions in general [12]. We performed the article abstraction step manually. While the independent reviewing method and ICC results indicate a low probability of coding error, we cannot completely exclude it. Variables regarding instructional design and assessment outcomes were often not explicitly declared and relied on reviewer judgment. We could not retrieve references and citations for 27 of the 251 articles (10.8%), and unique reference and citation matching relied on probabilistic algorithms that considered a small but nonnegligible error margin.

This study also has strengths. We performed a broad analysis of the literature and accounted for aspects that, to our knowledge, were not previously assessed, such as specific platforms and their features, and correlations assessed between learning end points and types of comparisons. We systematically summarized data using latent class analysis, which, to our knowledge, was for the first time performed in this setting. We described the article citation network and explored relationships between these and the article latent classes and CBL considerations, which, to our knowledge, were also for the first time performed in the field. Finally, we have made these results available through an interactive visualization that allows researchers to deeply explore articles [125].

### Implications

#### CBL Research Should Include Evidence From More Medical Schools

Our findings show that, while CBL in medical education varies significantly, most published articles are from medical schools in a small set of countries. Medical education has geographical specificities, which makes contributions from different geographical areas particularly enriching and should incite more schools to conduct research in this field.

#### Platform Development Should Avoid Reinventing the Wheel

Over 25 platforms and software projects were built specifically for medical education, despite having significant overlap in goals and features. While a few provided means to interact with learning materials, such as microscopy images [72], in ways not before possible, it would be worthwhile for researchers to try to develop open and generalizable systems addressing specific learning contexts that can be reused by researchers from other medical schools. Initiatives to design pluggable modules for mainstream learning management systems and reusable learning materials, such as learning objects [126], aimed at specific medical contexts should be preferred over building closed systems from scratch.

#### Instructional Design Considerations Should be Reported

The diversity of methods encompassed by CBL in terms of delivery medium, context, learner, and purpose, without reports of instructional design considerations, obfuscates the effect of different intervention aspects, for which instructional design—or the lack of it—is partly accountable [8,9,13,121]. The value of reporting interactive tools, such as quizzes with feedback, would also increase. Determining which principles best apply to different medical settings and medical knowledge is an issue of interest for future research [8].

#### Interventions Should Focus on Assessing Unexplored Outcomes

Studies generally report positive outcomes on knowledge, attitudes, and skills. Interestingly, studies that found no positive effect in any of the learning outcomes were often randomized controlled trials [66,83-86], some of them running in multiple institutions [127,128]. Studies with little or no description of the learning and teaching methodology had neutral findings [82,129]. Once again, the lack of comparable arms, such as CBL versus traditional instruction, makes it difficult to assess intervention outcomes. Furthermore, data showing that objective knowledge assessment and skills increase with interventions can be used in deeper ways. Real-time collection of student activity, together with objective performance assessment through multiple choice quizzes, may have predictive value. Judgments of knowledge together with other student activity metrics may provide data for a next generation of intelligent tutoring systems able to track, manage, and predict student performance [130]. An increase in studies reporting online activity measurements and correlations with other learning outcomes using reproducible tools, as described before, would generate useful evidence on the effectiveness of CBL methods in enhancing learning [131]. Metrics could include, for example, student communication style and sentiment [132,133] and time spent on different types of materials [134].

#### CBL Research Seems to be Progressing on the Right Track

Even though 25% of the articles seemed not to be based on common CBL literature, our findings suggest that research is moving toward favoring studies comparing CBL methods rather than comparison with traditional methods. Indeed, we found that, while traditional versus CBL articles were more numerous, articles comparing different CBL methods were cited more often than articles comparing CBL versus traditional settings. We take this as a sign that recommendations put forward by previous authors are being taken into consideration [8,9,11]. Articles in the instructional and online activity latent classes, as well as those citing the Cook et al meta-analysis [12], had more references and citations in common with other articles, demonstrating greater awareness of research in this field and possibly indicating future research directions.

#### A Further Push Into a Student-Centered Models is Key

The shift to student-centered models needs to continue. However, only a few reports put students at the center of the education process, focusing usually on aspects related to teaching [135]. Part of the success of CBL features comes from empowering students to conduct study sessions at their own pace, providing them with richer interactions with learning materials, and facilitating communication, which were not otherwise feasible. Promoting student self-directedness through social media and reward-based systems may lead to increased engagement and improved learning outcomes [136]. Active learning through engagement in collaborative user-generated content, facilitated communication, and feedback in which instructors act as moderators may further promote this change [137]. Engaging students in the creation of content can be a good way to help faculty cope with the increasing demand for learning material [138]. Social media tools such as wikis have been used in the medical context for various purposes [139], but in medical education they still are limited in their format, management, and collaborative features [140]. Other approaches using 3D virtual worlds may offer great potential to learners through immersive exploratory worlds and a rich feedback environment that may be used to engage learners and simulate real-world scenarios of medical doctors [140].

### Conclusions

We have come a long way in CBL in medical education. While the field is highly variable and some studies seemed to be unaware of advances in the field, recommendations on comparing different CBL methods seem to have been taken into consideration. Incorporating instructional design principles in the design of learning materials and developing further educational software in ways that can be shared between researchers are paths for further improvement. A focus on measuring online activity and correlating it with other outcomes may provide insights into ways to keep promoting student-centered approaches tailored to specific learning settings.

## Appendix

Multimedia Appendix 1 Search queries.

Multimedia Appendix 2 Educational software of abstracted papers.

Multimedia Appendix 3 Complete reference of abstracted papers.

Multimedia Appendix 4 Interactive article explorer.

## Abbreviations

BIC: Bayesian information criterion
CBL: computer-based learning
DOI: digital object identifier
ICC: intraclass correlation
MERSQI: Medical Education Research Study Quality Index
PRISMA: Preferred Reporting Items for Systematic Reviews and Meta-Analyses
