# Supplementary material for: What Are We Looking for in Computer-Based Learning Interventions in Medical Education? A Systematic Review
Source: J Med Internet Res. 2016 Aug 1;18(8):e204. doi: 10.2196/jmir.5461 (PMC4985611; doi:10.2196/jmir.5461)
Supplement: Multimedia Appendix 1 [file jmir_v18i8e204_app1.pdf]

Appendix 1

PubMed

```
(
    medical education OR
    education, medical[MeSH] OR
    medical students OR
    students, medical[MeSH]
) AND (
    evidence-based learning OR
    student-centered learning OR
    blended learning OR
    spaced learning OR
    e-learning
) AND (
    information technology OR
    e-learning software OR
    software[MeSH] OR
    software tool OR
    web-based platform OR
    blogging[MeSH] OR
    e-portfolio OR
    audience response system OR
    instant messaging OR
    streaming video OR
    computer simulation OR
    computer simulation[MeSH] OR
    computer games OR
    video games[MeSH] OR
    telecasts OR
    podcasts
) AND ("2003/01/01"[PDAT] : "2013/12/31"[PDAT])
```

Scopus

```
(
    ALL("medical education") OR
    ALL("medical students")
) AND (
ALL("evidence-based learning") OR
    ALL("student-centered learning") OR
    ALL("blended learning") OR
    ALL("spaced learning") OR
    ALL("e-learning")
) AND (
    ALL("information technology") OR
    ALL("e-learning software") OR
    ALL("software tool") OR
    ALL("web-based platform") OR
    ALL("e-portfolio") OR
    ALL("audience response system") OR
    ALL("instant messaging") OR
    ALL("streaming video") OR
    ALL("computer simulation") OR
    ALL("computer games") OR
    ALL("telecasts") OR
    ALL("podcasts")
)
AND PUBYEAR > 2002
AND PUBYEAR < 2014
AND LANGUAGE(english)
AND DOCTYPE(ar)
```

EBSCO Host

"medical education" OR "medical students"

AND

"evidence-based learning" OR

"student-centered learning" OR

"blended learning" OR

"spaced learning" OR

"e-learning"

AND

"information technology" OR

"e-learning software" OR

"software tool" OR

"web-based platform" OR

"e-portfolio" OR

"audience response system" OR

"instant messaging" OR

"streaming video" OR

"computer simulation" OR

"computer games" OR

"telecasts" OR

"podcasts"

Source TX All Text

Limit to: scholarly (peer reviewed) journals

Source types: Academic journals

Date: 2003 - 2013

Science Direct / Web of Knowledge

```
(
   "medical education" OR
   "medical students"
)
AND
(
   "evidence-based learning" OR
   "student-centered learning" OR
   "blended learning" OR
   "spaced learning" OR
   "e-learning"
)
AND
(
   "information technology" OR
   "e-learning software" OR
   "software tool" OR
   "web-based platform" OR
   "e-portfolio" OR
   "audience response system" OR
   "instant messaging" OR
   "streaming video" OR
   "computer simulation" OR
   "computer games" OR
   "telecasts" OR
   "podcasts"
)
```

Date: 2003 - 2013
