# Supplementary material for: What Are We Looking for in Computer-Based Learning Interventions in Medical Education? A Systematic Review
Source: J Med Internet Res. 2016 Aug 1;18(8):e204. doi: 10.2196/jmir.5461 (PMC4985611; doi:10.2196/jmir.5461)
Supplement: Multimedia Appendix 2 [file jmir_v18i8e204_app2.pdf]

Appendix 2 - Educational software

General educational software used in the studies

Software	Articles	Description
Blackboard [1–9]	8	Learning management system
Moodle [10–17]	8	Learning management system
WebCT [18–23]	6	Learning management system
Second Life [24,25]	2	Online virtual world
Adobe Connect [26]	1	Web-conference software
Angel LMS [27]	1	Learning management system
Blender [28]	1	Open-source 3D software
CLIX [29]	1	Learning management system
Desire2Learn [30]	1	Learning management system
Discourse LLC [31]	1	Virtual patient simulator
Confluence [32]	1	Team collaboration software
MediaWiki [33]	1	Wiki platform
Microsoft Virtual Meeting [34]	1	Web-conference software
Sakai [35]	1	Learning management system

Medical education software used in the studies

Software	Articles	Description
CASUS [36–39]	3	Virtual patient simulator
HINTS [40–42]	3	Virtual patient simulator
INMEDEA [43–45]	3	Virtual patient simulator
Web-SP [14,46,47]	3	Virtual patient simulator
MEFANET [48,49]	2	Learning management system
EleUM [2]	1	Learning management system
ICFAS [50]	1	Web conferencing and LMS
GeriaSims [51]	1	Virtual patient simulator
FACS [52]	1	Virtual patient simulator
EMSAVE [53]	1	Serious 3D game
Xerte [54]	1	Learning management system
EKGtolkning [55]	1	Electrocardiography learning
INDIAM [56]	1	Mammogram learning
CaseTrain [57]	1	Virtual patient simulator
EEMeC [58]	1	Learning management system
ISP [59]	1	Virtual patient simulator
NLE [60]	1	Learning management system
Surgent [61]	1	Virtual patient simulator
SIMmersion [62]	1	Virtual patient simulator
Schoolbook [63]	1	Learning management system
Radiology Teacher [64]	1	Radiology cases learning system
IVIMEDS [65]	1	Virtual patient simulator
MyMiCROscope [66]	1	Virtual microscope software
MyCourses [67]	1	Learning management system
LRSMed [68]	1	Learning management system

References

1. Wiecha JM, Gramling R, Joachim P, Vanderschmidt H. Collaborative e-learning using streaming video and asynchronous discussion boards to teach the cognitive foundation of medical interviewing: a case study. J Med Internet Res 2003;5(2).

2. De Leng BA, Dolmans DH, Muijtjens AM, Van Der Vleuten CP. Student perceptions of a virtual learning environment for a problem-based learning undergraduate medical curriculum. Med Educ 2006;40(6):568–575.

3. Wiecha JM, Chetty V, Pollard T, Shaw PF. Web-based versus face-to-face learning of diabetes management: the results of a comparative trial of educational methods. Fam Med-Kans CITY- 2006;38(9):647.

4. Thakore H, McMahon T. Designing an Interactive Multimedia Rich Tutorial for Medical Students: Beyond a'Book on a Screen'. J Vis Commun Med 2008;31(1):4–10.

5. Cobus L. Using blogs and wikis in a graduate public health course. Med Ref Serv Q 2009;28(1):22–32.

6. Gray K, Tobin J. Introducing an online community into a clinical education setting: a pilot study of student and staff engagement and outcomes using blended learning. BMC Med Educ 2010;10(1):6.

7. Desai T, Stankeyeva D, Chapman A, Bailey J. Nephrology fellows show consistent use of, and improved knowledge from, a nephrologist-programmed teaching instrument. J Nephrol 2011;24(3):345.

8. Maggio MP, Hariton-Gross K, Gluch J. The use of independent, interactive media for education in dental morphology. J Dent Educ 2012;76(11):1497–1511.

9. Stebbings S, Bagheri N, Perrie K, Blyth P, McDonald J. Blended learning and curriculum renewal across three medical schools: The rheumatology module at the University of Otago. Australas J Educ Technol 2012;28(7):1176–1189.

10. Shah I, Walters M, McKillop J. Acute medicine teaching in an undergraduate medical curriculum: a blended learning approach. Emerg Med J 2008;25(6):354–357.

11. Kukolja Taradi S, \DJogaš Z, Dabić M, Drenjančević Perić I. Scaling-up undergraduate medical education: enabling virtual mobility by online elective courses. Croat Med J 2008;49(3):344–351.

12. Puljak L, Sapunar D. Web-Based Elective Courses for Medical Students: An Example in Pain. Pain Med 2011;12(6):854–863.

13. Sparacia G, Cannizzaro F, D'Alessandro DM, D'Alessandro MP, Caruso G, Lagalla R. Initial Experiences in Radiology e-Learning 1. Radiographics 2007;27(2):573–581.

14. Botezatu M, Hult H akan, Tessma MK, Fors UG. Virtual patient simulation for learning and assessment: Superior results in comparison with regular course exams. Med Teach 2010;32(10):845–850.

15. Seluakumaran K, Jusof FF, Ismail R, Husain R. Integrating an open-source course management system (Moodle) into the teaching of a first-year medical physiology course: a case study. Adv Physiol Educ 2011;35(4):369–377.

16. Worm BS, Jensen K. Does peer learning or higher levels of e-learning improve learning abilities? A randomized controlled trial. Med Educ Online 2013;18.

17. Iyeyasu JN, Sabbatini RME, Carvalho KM de. The development and evaluation of a distance learning system in ophthalmology. Rev Bras Educ Médica 2013;37(1):96–102.

18. Naidr J, Adla T, Janda A, Feberova J, Kasal P, Hladikova M. Long-term retention of knowledge after a distance course in medical informatics at Charles University Prague. Teach Learn Med 2004;16(3):255–259.

19. Taradi SK, Taradi M, Radić K, Pokrajac N. Blending problem-based learning with Web technology positively impacts student learning outcomes in acid-base physiology. Adv Physiol Educ 2005;29(1):35–39.

20. Bernhardt J, Hye F, Thallinger S, Bauer P, Ginter G, Smolle J. Simulation of a mycological KOH preparation–e-learning as a practical dermatologic exercise in an undergraduate medical curriculum. JDDG J Dtsch Dermatol Ges 2009;7(7):597–601.

21. Kandasamy T, Fung K. Interactive Internet-based cases for undergraduate otolaryngology education. Otolaryngol Neck Surg 2009;140(3):398–402.

22. Hu A, Wilson T, Ladak H, Haase P, Doyle P, Fung K. Evaluation of a three-dimensional educational computer model of the larynx: voicing a new direction. J Otolaryngol-Head Neck Surg J Oto-Rhino-Laryngol Chir Cervico-Faciale 2010;39(3):315–322.

23. Tan S, Hu A, Wilson T, Ladak H, Haase P, Fung K. Role of a computer-generated three-dimensional laryngeal model in anatomy teaching for advanced learners. J Laryngol Otol 2012;126(04):395–401.

24. Boulos MNK, Hetherington L, Wheeler S. Second Life: an overview of the potential of 3-D virtual worlds in medical and health education. Health Inf Libr J 2007;24(4):233–245.

25. Andrade AD, Cifuentes P, Mintzer MJ, Roos BA, Anam R, Ruiz JG. Simulating geriatric home safety assessments in a three-dimensional virtual world. Gerontol Geriatr Educ 2012;33(3):233–252.

26. Foroudi F, Pham D, Bressel M, Tongs D, Rolfo A, Styles C, et al. The utility of e-Learning to support training for a multicentre bladder online adaptive radiotherapy trial (TROG 10.01-BOLART). Radiother Oncol 2013;109(1):165–169.

27. Ruiz JG, Smith M, Rodriguez O, Van Zuilen MH, Mintzer MJ. An interactive e-learning tutorial for medical students on how to conduct the performance-oriented mobility assessment. Gerontol Geriatr Educ 2007;28(1):51–60.

28. Codd AM, Choudhury B. Virtual reality anatomy: Is it comparable with traditional methods in the teaching of human forearm musculoskeletal anatomy? Anat Sci Educ 2011;4(3):119–125.

29. Raupach T, Münscher C, Pukrop T, Anders S, Harendza S. Significant increase in factual knowledge with web-assisted problem-based learning as part of an undergraduate cardio-respiratory curriculum. Adv Health Sci Educ 2010;15(3):349–356.

30. Kumar AB, Hata JS, Bayman EO, Krishnan S. Implementing a hybrid web-based curriculum for an elective medical student clerkship in a busy surgical intensive care unit (ICU): Effect on test and satisfaction scores. J Surg Educ 2013;70(1):109–116.

31. Yang RL, Hashimoto DA, Predina JD, Bowens NM, Sonnenberg EM, Cleveland EC, et al. The virtual-patient pilot: testing a new tool for undergraduate surgical education and assessment. J Surg Educ 2013;70(3):394–401.

32. Harris ST, Zeng X. Using wiki in an online record documentation systems course. Perspect Health Inf Manag Am Health Inf Manag Assoc 2008;5.

33. Durosaro O, Lachman N, Pawlina W. Use of knowledge-sharing Web-based portal in gross and microscopic anatomy. Ann Acad Med Singap 2008;37(12):998.

34. Carriero A, Zobel BB, Bonomo L, Meloni G, Cotroneo A, Cova M, et al. E-learning in radiology: Italian multicentre experience. Radiol Med (Torino) 2011;116(7):989–999.

35. McCleskey PE. Clinic teaching made easy: A prospective study of the American Academy of Dermatology core curriculum in primary care learners. J Am Acad Dermatol 2013;69(2):273–279.

36. Fischer MR, Schauer S, Gräsel C, Baehring T, Mandl H, Gärtner R, et al. [CASUS model trial. A computer-assisted author system for problem-oriented learning in medicine]. Z Für Ärztl Fortbild 1996 Aug;90(5):385–389. PMID: 9157728

37. Abendroth M, Harendza S, Riemer M. Clinical decision making: a pilot e-learning study. Clin Teach 2013;10(1):51–55.

38. Maier EM, Hege I, Muntau AC, Huber J, Fischer MR. What are effects of a spaced activation of virtual patients in a pediatric course? BMC Med Educ 2013;13(1):45.

39. Radon K, Kolb S, Reichert J, Baumeister T, Fuchs R, Hege I, et al. Case-based e-learning in occupational medicine- the NetWoRM Project in Germany. Ann Agric Environ Med 2006;13(1):93–98.

40. Chen L-S, Cheng Y-M, Weng S-F, Chen Y-G, Lin C-H. Applications of a time sequence mechanism in the simulation cases of a web-based medical problem-based learning system. Educ Technol Soc 2009;12(1):149.

41. Cheng Y-M, Chen L-S, Huang H-C, Weng S-F, Chen Y-G, Lin C-H. Building a general purpose pedagogical agent in a web-based multimedia clinical simulation system for medical education. Learn Technol IEEE Trans On 2009;2(3):216–225.

42. Cheng Y-M, Kuo S-H, Lou S-J, Shih R-C. The Effect of Applying Online PBL Case System to Multiple Disciplines of Medical Education. Turk Online J Educ Technol-TOJET 2012;11(4):283–294.

43. Horstmann M, Horstmann C, Renninger M. Case Creation and E-Learning in a Web-Based Virtual Department of Urology Using the INMEDEA Simulator. Nephro-Urol Mon 2011;4(01):356–360.

44. Funke K, Bonrath E, Mardin WA, Becker JC, Haier J, Senninger N, et al. Blended learning in surgery using the Inmedea Simulator. Langenbecks Arch Surg 2013;398(2):335–340.

45. Wünschel M, Leichtle U, Wülker N, Kluba T. Using a web-based orthopaedic clinic in the curricular teaching of a German university hospital: analysis of learning effect, student usage and reception. Int J Med Inf 2010;79(10):716–721.

46. Botezatu M, Hult H akan, Tessma MK, Fors U. Virtual patient simulation: Knowledge gain or knowledge loss? Med Teach 2010;32(7):562–568.

47. Gunning WT, Fors UG. Virtual patients for assessment of medical student ability to integrate clinical and laboratory data to develop differential diagnoses: comparison of results of exams with/without time constraints. Med Teach 2012;34(4):e222–e228.

48. Schwarz D, Štourač P, Komenda M, Harazim H, Kosinová M, Gregor J, et al. Interactive algorithms for teaching and learning acute medicine in the network of medical faculties MEFANET. J Med Internet Res 2013;15(7).

49. Komenda M, Schwarz D, Feberová J, Štípek S, Mihál V, Dušek L. Medical faculties educational network: Multidimensional quality assessment. Comput Methods Programs Biomed 2012;108(3):900–909.

50. Kneebone R, Bello F, Nestel D, Mooney N, Codling A, Yadollahi F, et al. Learner-centred feedback using remote assessment of clinical procedures. Med Teach 2008;30(8):795–801.

51. Orton E, Mulhausen P. E-learning virtual patients for geriatric education. Gerontol Geriatr Educ 2008;28(3):73–88.

52. Leung JY, Critchley LA, Yung AL, Kumta SM. Introduction of virtual patients onto a final year anesthesia course: Hong Kong experience. Adv Med Educ Pract 2011;2:71.

53. Buttussi F, Pellis T, Vidani AC, Pausler D, Carchietti E, Chittaro L. Evaluation of a 3D serious game for advanced life support retraining. Int J Med Inf 2013;82(9):798–809.

54. Daunt LA, Umeonusulu PI, Gladman JR, Blundell AG, Conroy SP, Gordon AL. Undergraduate teaching in geriatric medicine using computer-aided learning improves student performance in examinations. Age Ageing 2013;42(4):541–544.

55. Nilsson M, Bolinder G, Held C, Johansson B-L, Fors U, Östergren J. Evaluation of a web-based ECG-interpretation programme for undergraduate medical students. BMC Med Educ 2008;8(1):25.

56. Guliato D, Bôaventura RS, Maia MA, Rangayyan RM, Simedo MS, Macedo TA. INDIAM—an e-learning system for the interpretation of mammograms. J Digit Imaging 2009;22(4):405–420.

57. Diessl S, Verburg FA, Hoernlein A, Schumann M, Luster M, Reiners C. Evaluation of an internet-based e-learning module to introduce nuclear medicine to medical students: a feasibility study. Nucl Med Commun 2010;31(12):1063–1067.

58. Ellaway R, Dewhurst D, Cumming A. Managing and supporting medical education with a virtual learning environment: the Edinburgh Electronic Medical Curriculum. Med Teach 2003;25(4):372–380.

59. Courteille O, Bergin R, Courteille O, Bergin R, Stockeld D, Ponzer S, et al. The use of a virtual patient case in an OSCE-based exam-a pilot study. Med Teach 2008;30(3):e66–e76.

60. Roberts C, Lawson M, Newble D, Self A. Managing the learning environment in undergraduate medical education: the Sheffield approach. Med Teach 2003;25(3):282–286.

61. Corrigan M, Reardon M, Shields C, Redmond H. “SURGENT”—student e-learning for reality: The application of interactive visual images to problem-based learning in undergraduate surgery. J Surg Educ 2008;65(2):120–125.

62. Fleming M, Olsen D, Stathes H, Boteler L, Grossberg P, Pfeifer J, et al. Virtual reality skills training for health care professionals in alcohol screening and brief intervention. J Am Board Fam Med 2009;22(4):387–398.

63. Citak M, Calafi A, Kendoff D, Kupka T, Haasper C, Behrends M, et al. An internet based learning tool in orthopaedic surgery: preliminary experiences and results. Technol Health Care 2009;17(2):141–148.

64. Talanow R. Radiology Teacher: a free, Internet-based radiology teaching file server. J Am Coll Radiol 2009;6(12):871–875.

65. Gormley GJ, Mcglade K, Thomson C, Mcgill M, Sun J. A virtual surgery in general practice: Evaluation of a novel undergraduate virtual patient learning package. Med Teach 2011;33(10):e522–e527.

66. Schmidt C, Reinehr M, Leucht O, Behrendt N, Geiler S, Britsch S. MyMiCROscope—Intelligent virtual microscopy in a blended learning model at Ulm University. Ann Anat-Anat Anz 2011;193(5):395–402.

67. Shaffer K, Small JE. Blended learning in medical education: use of an integrated approach with web-based small group modules and didactic instruction for teaching radiologic anatomy. Acad Radiol 2004 Sep;11(9):1059–70. PMID: 15350588

68. Geueke M, Stausberg J. A meta-data-based learning resource server for medicine. Comput Methods Programs Biomed 2003;72(3):197–208.
