# Supplementary material for: What Are We Looking for in Computer-Based Learning Interventions in Medical Education? A Systematic Review
Source: J Med Internet Res. 2016 Aug 1;18(8):e204. doi: 10.2196/jmir.5461 (PMC4985611; doi:10.2196/jmir.5461)
Supplement: Multimedia Appendix 3 [file jmir_v18i8e204_app3.pdf]

Appendix 3

Definition of study variables

**Teaching method**	
B	Blended Learning
E	E-learning
**Citations**	
CC1	Article is part of the main connected component of the studied articles graph
CC0	Article is not part of the main connected component of the studied articles graph
Ci0	No citations were found for this article at the time of this study
Ci1	Up to 10 citation were found for this article at the time of this study
Ci2	More than 10 citations were found for this article at the time of this study
Rf0	Article has less than 2 references in common with other articles in this study
Rf1	Article has 2 to 10 references in common with other articles in this study
Rf2	Article has more than 10 references in common with other articles in this study
**Platform support**	
A	Audio Response System
B	Blog
C	CD/DVD ROM
E	Ebook
We	Website
M	Email
Po	Podcast
Pt	Portfolio
T	Video Conference
Wi	Wiki
**Media features**	
An	Animation
Au	Audio
D	Diagram
I	Image

Description of platform features

Ref	Year	Subject	Teach. Method	Platform support	Media features	Interactive features	Accessibility features	Instructional design principles	Citations
[1]	2011	evidence based medicine	B	B A	n/a	n/a	n/a	n/a	Rf0 Ci1 CC1
[2]	2012	reproductive medicine	E	We E	T I D An	Q I F P	I F	Pr Se Me	Rf2 Ci1 CC1
[3]	2013	general	B	We	T I D	S V Q I F	n/a	Cn Se Mo Me	Rf1 Ci1 CC0
[4]	2012	general	B	We	T	Cb S	F	n/a	Rf1 Ci1 CC1
[5]	2009	anatomy	B	A	T I	I F	n/a	n/a	Rf0 Ci1 CC1
[6]	2011	oncology	E	We	T I D	C Q I F P	n/a	n/a	Rf2 Ci1 CC1
[7]	2010	dermatology	E	Po	V Au	n/a	n/a	n/a	Rf1 Ci1 CC0
[8]	2012	geriatrics	E	We	n/a	I	n/a	n/a	Rf1 Ci1 CC0
[9]	2012	cardiology	E	We	n/a	n/a	n/a	n/a	Rf2 Ci1 CC1
[10]	2012	professionalism	E	n/a	V	Q F P	n/a	Se	Rf0 Ci0 CC0
[11]	2010	anatomy genetics histology	B	T	V	n/a	n/a	n/a	Rf2 Ci2 CC1
[12]	2012	emergency	E	We M	T I	I	F	n/a	Rf1 Ci1 CC0
[13]	2008	pulmonology	E	We Wi	T V Au I D	Q I F	F	Me	Rf0 Ci1 CC1
[14]	2008	dermatology	E	We	T I D	S I F	H	n/a	Rf2 Ci1 CC1
[15]	2010	ophthalmology	E	We	T I D	Q I	n/a	n/a	Rf2 Ci1 CC1
[16]	2009	general surgery	E	We Po	T I	n/a	n/a	n/a	Rf1 Ci2 CC1
[17]	2013	general	B	E	n/a	n/a	n/a	n/a	Rf0 Ci0 CC0
[18]	2010	cardiology haematology	E	We	T I D	S V C I F P	n/a	n/a	Rf0 Ci2 CC1

Ref	Year	Subject	Teach. Method	Platform support	Media features	Interactive features	Accessibility features	Instructional design principles	Citations
[18]	2010	cardiology haematology	B	We	T I D	S V C I F P	n/a	n/a	Rf0 Ci1 CC1
[19]	2007	general	E	We	n/a	Cb S G I F	I F	n/a	Rf1 Ci2 CC1
[20]	2012	immunology	B	We	An	n/a	n/a	n/a	Rf2 Ci1 CC1
[21]	2011	general	E	We	T D	n/a	n/a	n/a	Rf0 Ci0 CC0
[22]	2009	general	B	n/a	V	n/a	n/a	n/a	Rf0 Ci1 CC1
[22]	2009	general	B	T	n/a	n/a	n/a	n/a	Rf0 Ci1 CC1
[23]	2013	cardiology	E	We	V	Q I F	n/a	n/a	Rf0 Ci0 CC0
[24]	2004	pathology	E	We	T	C Q I	F Ca	n/a	Rf0 Ci1 CC1
[25]	2013	emergency	E	We	n/a	S G I F	n/a	n/a	Rf0 Ci1 CC1
[26]	2008	professionalism	B	We	T V Au I D	Cb I F	I F D	Me	Rf2 Ci2 CC1
[27]	2004	general	E	Pt	n/a	n/a	n/a	n/a	Rf1 Ci2 CC0
[28]	2012	general	E	T	n/a	n/a	n/a	n/a	Rf1 Ci1 CC0
[29]	2011	radiology	E	T	V Au	n/a	n/a	n/a	Rf1 Ci1 CC0
[30]	2003	primary	E	We	T I D	C I F P	n/a	Se	Rf0 Ci1 CC1
[31]	2012	psychiatry	E	T	V	n/a	n/a	n/a	Rf2 Ci1 CC1
[32]	2009	general	B	We	T I D	C I P	n/a	n/a	Rf1 Ci1 CC1
[33]	2009	general	E	We	T Au I D	S V C I F P	n/a	n/a	Rf1 Ci1 CC1
[34]	2012	radiology	n/a	We	n/a	I F	n/a	n/a	Rf0 Ci0 CC1
[35]	2008	professionalism	B	B	T	n/a	n/a	n/a	Rf2 Ci2 CC1

Ref	Year	Subject	Teach. Method	Platform support	Media features	Interactive features	Accessibility features	Instructional design principles	Citations
[36]	2009	orthopaedics	B	We	T V I D	n/a	n/a	Me	Rf0 Ci1 CC0
[37]	2009	public health	B	B Wi	T	Cb	F	n/a	Rf1 Ci1 CC0
[38]	2012	ophthalmology	B	We	n/a	n/a	n/a	n/a	Rf0 Ci0 CC0
[39]	2011	anatomy	E	We	T	I	n/a	n/a	Rf1 Ci2 CC1
[40]	2012	immunology	E	We B	T	n/a	n/a	n/a	Rf0 Ci0 CC0
[41]	2012	general surgery	E	We	T	Cb I	n/a	n/a	Rf2 Ci1 CC1
[42]	2008	general surgery	B	We	T I	C I F	n/a	Me	Rf1 Ci2 CC1
[43]	2008	general surgery	B	We	n/a	S	n/a	n/a	Rf1 Ci1 CC1
[44]	2012	emergency	E	We	n/a	Cb G I F	n/a	n/a	Rf1 Ci1 CC0
[45]	2009	anaesthesia	B	We	T V Au I D	S C Q I F P	n/a	Me	Rf1 Ci1 CC0
[46]	2012	cardiovascular surgery	E	We	T V Au I	Q I F P	n/a	n/a	Rf1 Ci0 CC0
[47]	2013	geriatrics	B	We	T V Au I D	Q I F	n/a	Me	Rf0 Ci1 CC1
[48]	2011	physiology	B	We	T V Au I D An	S C Q I F P	n/a	Ch Pr Se Mo Me Pe	Rf0 Ci1 CC1
[49]	2011	anatomy	B	We	T V I D	Cb Q I F P	D	Me	Rf1 Ci1 CC0
[50]	2012	general	B	We	n/a	n/a	F D	n/a	Rf1 Ci1 CC0
[51]	2007	evidence based medicine	E	We C	T V Au	n/a	n/a	Me	Rf2 Ci2 CC1
[52]	2012	emergency	B	We	V Au	n/a	n/a	n/a	Rf2 Ci1 CC1
[53]	2012	biochemistry	B	We	T V Au I	Cb	F	n/a	Rf1 Ci0 CC0

Ref	Year	Subject	Teach. Method	Platform support	Media features	Interactive features	Accessibility features	Instructional design principles	Citations
[54]	2006	general	B	We	T V Au I	n/a	F	n/a	Rf2 Ci0 CC1
[55]	2007	general	E	We	V	n/a	n/a	n/a	Rf1 Ci1 CC1
[56]	2012	anatomy	E	We	n/a	S I	n/a	Ch	Rf1 Ci1 CC0
[57]	2011	nephrology	E	We	T V Au I D	Q I	F	n/a	Rf2 Ci0 CC1
[58]	2009	radiology	E	We	T I D	n/a	n/a	Ch Si Cn Pr Se Mo Me	Rf1 Ci0 CC0
[59]	2010	nuclear medicine	E B	We	T V I D	C Q I F	n/a	Me	Rf1 Ci1 CC1
[60]	2005	radiology	E	We	T I D An	S I F	n/a	Cn Me	Rf1 Ci1 CC1
[61]	2012	geriatrics	B	We T Pt	T V Au	G	n/a	n/a	Rf1 Ci2 CC1
[62]	2008	anatomy	B	We	T I D	Cb	F	n/a	Rf1 Ci1 CC1
[63]	2008	palliative	E	We	T	Q	n/a	n/a	Rf1 Ci1 CC1
[64]	2003	n/a	B	We Pt	T I D	Cb Q I F P	F Ca	Se Me	Rf2 Ci1 CC1
[65]	2005	dermatology	E	We	T I D	C Q I F P	n/a	Si Cn Pr Se Me	Rf2 Ci1 CC0
[66]	2013	paediatrics	B	We	T I D	C Q I F	n/a	Si Pr Se	Rf1 Ci1 CC0
[67]	2012	infectious diseases	B	We T Po	n/a	n/a	D	n/a	Rf0 Ci0 CC0
[68]	2009	psychiatry	E	We	T V Au I	S I F	n/a	n/a	Rf1 Ci1 CC0
[69]	2013	radiology	E	We	n/a	n/a	n/a	n/a	Rf1 Ci1 CC1
[70]	2005	cardiovascular surgery	E	We	T V Au I D An	S I	n/a	Cn V	Rf0 Ci0 CC1
[71]	2006	cardiovascular surgery	E	We	T V Au I D An	S I	n/a	Cn	Rf2 Ci1 CC1

Ref	Year	Subject	Teach. Method	Platform support	Media features	Interactive features	Accessibility features	Instructional design principles	Citations
[72]	2012	general surgery	B	We	T V I	Cb S C Q I F P	n/a	Me	Rf1 Ci1 CC1
[73]	2006	primary	B	We Pt	T	n/a	n/a	n/a	Rf0 Ci1 CC1
[74]	2013	general	B	We T	n/a	Cb	n/a	n/a	Rf0 Ci1 CC1
[75]	2003	general	E	We	T Au I D	C	D	n/a	Rf1 Ci1 CC1
[76]	2010	paediatrics	E	We Po	T V Au I D	Cb C Q I F	n/a	Me	Rf2 Ci1 CC0
[77]	2012	histology	E	B	n/a	C	n/a	n/a	Rf2 Ci0 CC0
[78]	2013	otolaryngology	B	Pt	T V Au I	Q I F P	D	n/a	Rf0 Ci1 CC0
[79]	2011	paediatrics	E	We	V An	n/a	n/a	n/a	Rf2 Ci1 CC0
[80]	2011	primary	E	We	T V Au I	V C I	n/a	n/a	Rf2 Ci1 CC1
[81]	2006	radiology	E B	We	T V I D	Cb Q I F	F D	Si Cn Pr Se Me	Rf0 Ci1 CC1
[82]	2008	anatomy	E	n/a	n/a	n/a	n/a	n/a	Rf1 Ci1 CC1
[83]	2012	otolaryngology	E	We	T I	C Q	n/a	n/a	Rf1 Ci1 CC0
[84]	2010	general	B	We	n/a	Cb	F	n/a	Rf1 Ci1 CC0
[85]	2010	palliative	B	We	T V I	C I	n/a	Me	Rf0 Ci2 CC0
[86]	2007	genetics	E	We	T An	I	D	Se	Rf0 Ci2 CC0
[87]	2013	epidemiology	B	We	T V Au I	n/a	F	n/a	Rf1 Ci0 CC1
[88]	2009	radiology	E	We	T I D	S Q I F	n/a	Me	Rf0 Ci1 CC1
[89]	2012	emergency	B	We	n/a	V	n/a	n/a	Rf0 Ci1 CC1
[90]	2013	general surgery	E	We M	T	C Q I	n/a	n/a	Rf1 Ci1 CC1

Ref	Year	Subject	Teach. Method	Platform support	Media features	Interactive features	Accessibility features	Instructional design principles	Citations
[91]	2010	evidence based medicine	E	We	n/a	n/a	D	n/a	Rf2 Ci2 CC1
[92]	2011	physics	E	We	T I D	Cb S V Q I F	n/a	n/a	Rf2 Ci2 CC0
[93]	2011	general	E	We	T V Au I D	Q I	n/a	Cn Se Mo Me	Rf1 Ci1 CC1
[94]	2012	advanced life support	E	We	n/a	n/a	D	n/a	Rf2 Ci0 CC0
[95]	2010	pulmonology	B	We	T	Q F	F	n/a	Rf0 Ci0 CC0
[96]	2008	informatics	E	Wi	n/a	Cb	D	n/a	Rf0 Ci1 CC0
[97]	2013	psychiatry	E	We	T V Au I	V I	n/a	n/a	Rf2 Ci1 CC0
[98]	2013	reproductive medicine	E	We	n/a	Q	n/a	n/a	Rf0 Ci0 CC0
[99]	2008	psychology	B	We C Pt	T V Au I	C Q I F	n/a	Me V	Rf2 Ci1 CC1
[100]	2012	professionalism	B	We	V	Q	n/a	n/a	Rf2 Ci1 CC0
[101]	2009	neurology	B	We	T V Au	Q	n/a	Me	Rf2 Ci1 CC0
[102]	2012	professionalism	E	We	T V Au I	Q F	n/a	n/a	Rf0 Ci0 CC0
[103]	2010	neuroscience	B	We	T I D	Q I F	n/a	Cn Me	Rf0 Ci0 CC1
[104]	2013	pharmacology	E	C Po	n/a	n/a	n/a	n/a	Rf1 Ci1 CC1
[105]	2006	radiology	E	We	T V Au I D An	Q F	n/a	n/a	Rf1 Ci1 CC1
[106]	2009	physiology	E	We	D	S Q I F	H	Ch Si Cn Pr Se Mo Me Pe	Rf1 Ci1 CC1
[107]	2009	urology	B	We	T I	S C Q I F	n/a	Me	Rf0 Ci1 CC1
[108]	2011	urology	E	We	T I D	S C I F P	n/a	Se	Rf2 Ci1 CC1

Ref	Year	Subject	Teach. Method	Platform support	Media features	Interactive features	Accessibility features	Instructional design principles	Citations
[109]	2006	radiology	E	We	T I	n/a	n/a	Cn	Rf0 Ci0 CC1
[110]	2010	otolaryngology	E	We	T	S I	n/a	n/a	Rf1 Ci2 CC0
[111]	2013	general	B	We	n/a	V I F P	n/a	n/a	Rf1 Ci1 CC0
[112]	2009	general	B	We Pt	T	Cb C Q P	n/a	n/a	Rf1 Ci0 CC1
[113]	2011	ophthalmology	B	We	n/a	n/a	n/a	n/a	Rf0 Ci0 CC1
[114]	2012	anatomy	B	We	V Au	n/a	n/a	n/a	Rf0 Ci2 CC1
[115]	2012	gynaecology	E	T	T V Au I	n/a	n/a	n/a	Rf0 Ci0 CC0
[116]	2009	dermatology	B	We	T V Au I D An	G Q I F P	D	n/a	Rf1 Ci1 CC0
[117]	2009	otolaryngology	E	We	T I	C Q I	n/a	n/a	Rf2 Ci2 CC1
[118]	2008	urology	E	We	T Au I D	Q I F	n/a	Pr Se Me	Rf2 Ci2 CC1
[119]	2009	urology	E	M	T	Q	n/a	n/a	Rf2 Ci1 CC1
[120]	2012	urology	E	We M	T	G Q I F P	n/a	n/a	Rf2 Ci1 CC1
[121]	2007	urology	E	M	n/a	Q	n/a	n/a	Rf2 Ci1 CC1
[122]	2009	urology	E	We M	T I D	Q F	n/a	Se Me	Rf2 Ci1 CC1
[123]	2007	urology	B	We M	T Au	C Q I	n/a	n/a	Rf1 Ci1 CC1
[124]	2010	pathology	E	We M	T I D	Q F	n/a	Se Me	Rf2 Ci1 CC1
[125]	2011	physiology	E	We	T V Au I D	Cb S Q I F P	F	Pr Me V	Rf2 Ci1 CC1
[126]	2013	anatomy	E	We	n/a	S I	n/a	n/a	Rf2 Ci1 CC1
[127]	2010	general	E	We	T V Au I D	S C Q I F	n/a	Me	Rf0 Ci0 CC0

Ref	Year	Subject	Teach. Method	Platform support	Media features	Interactive features	Accessibility features	Instructional design principles	Citations
[128]	2008	general	B	We	n/a	Cb S F P	I	Me	Rf1 Ci1 CC0
[129]	2011	physiology	E	We	T V Au I D An	S I F	n/a	n/a	Rf1 Ci1 CC1
[130]	2006	occupational medicine	E	We	T V Au I D	Q I F P	n/a	Me	Rf1 Ci1 CC1
[131]	2012	general	E	We	T V Au I D	Cb	n/a	n/a	Rf1 Ci1 CC1
[132]	2012	advanced life support	B	We	T V I	S V Q I F	n/a	Ch Si Cn Se Me	Rf1 Ci1 CC0
[133]	2005	haematology	B	We	T I	C Q F P	n/a	Cn Pr Se Me	Rf0 Ci1 CC1
[134]	2010	biochemistry	B	We	T Au I D An	Cb S Q I F	n/a	n/a	Rf2 Ci2 CC1
[135]	2008	anatomy genetics histology	E B	We	T I D	G Q I F	I	Me	Rf1 Ci2 CC1
[135]	2008	physiology	B	We Wi	n/a	Cb I	I F	n/a	Rf0 Ci2 CC1
[136]	2008	evidence based medicine	E	We C	T Au I	I	D	Me	Rf2 Ci1 CC1
[137]	2013	emergency	B	We Po	T V Au I D	Q	n/a	Se	Rf2 Ci1 CC1
[138]	2010	paediatrics	B	We	T V Au I D	S V I	n/a	Me	Rf0 Ci1 CC0
[139]	2013	paediatrics	B	We	T V Au I D	S Q I F	n/a	Cn Se Mo Me Pe	Rf1 Ci1 CC1
[140]	2012	radiology	E	We	T I	Q I	n/a	Me	Rf0 Ci1 CC1
[141]	2011	anaesthesia	B	We	T I D	S V C Q I F P	n/a	Se	Rf0 Ci1 CC0
[142]	2009	primary	B	We	T V Au I D An	C Q I	F	n/a	Rf2 Ci1 CC1
[143]	2006	cardiovascular surgery	B	n/a	n/a	S I	n/a	n/a	Rf0 Ci0 CC0
[144]	2005	microbiology	E	We	T I D	G F P	n/a	Me	Rf0 Ci1 CC0

Ref	Year	Subject	Teach. Method	Platform support	Media features	Interactive features	Accessibility features	Instructional design principles	Citations
[145]	2006	informatics	E	We	n/a	n/a	F	n/a	Rf0 Ci2 CC1
[146]	2011	psychiatry	B	We M Wi	T	C	F D	n/a	Rf2 Ci1 CC0
[147]	2010	telemedicine	B	T	n/a	Cb	n/a	n/a	Rf0 Ci1 CC0
[148]	2009	radiology	B	We Po	T V Au I D	I F	n/a	n/a	Rf0 Ci0 CC0
[149]	2010	anatomy	E	We	n/a	S I	n/a	n/a	Rf1 Ci1 CC0
[150]	2013	paediatrics	B	We	T V Au I D	Cb S V C Q I F	n/a	Se Me	Rf1 Ci0 CC0
[151]	2008	evidence based medicine	B	We	T	I	n/a	n/a	Rf1 Ci1 CC1
[152]	2011	urology	E	We	T V Au I D An	F	n/a	n/a	Rf1 Ci1 CC1
[153]	2011	radiology	E	We	T I	C Q I F P	n/a	Cn Se Mo Me Pe	Rf0 Ci2 CC1
[154]	2005	microbiology	B	We M	T I An	n/a	I F D	n/a	Rf2 Ci2 CC0
[155]	2009	urology	E	M	T	n/a	n/a	n/a	Rf1 Ci1 CC1
[156]	2013	dermatology	E	We	T I	Q	n/a	n/a	Rf1 Ci0 CC0
[157]	2010	infectious diseases	B	We	n/a	C I	n/a	n/a	Rf2 Ci1 CC0
[158]	2012	neurology	E	We	T I	Q I	n/a	Cn Me	Rf0 Ci0 CC0
[159]	2009	geriatrics	E	We	T	n/a	n/a	n/a	Rf1 Ci1 CC1
[160]	2011	pharmacology	B	We	T V Au I D	Q I	I F	Me	Rf2 Ci1 CC0
[161]	2010	paediatrics	B	We Wi	T V I	C I	I	Me	Rf1 Ci1 CC1
[162]	2010	biochemistry	E	We	T V I	G I F P	n/a	Me	Rf1 Ci2 CC1

Ref	Year	Subject	Teach. Method	Platform support	Media features	Interactive features	Accessibility features	Instructional design principles	Citations
[163]	2012	haematology	E	We	T V Au I D	C Q I F P	n/a	Ch Si Cn Pr Se Mo Me V	Rf2 Ci2 CC1
[164]	2010	informatics	B	We M	T	Q	D Ca	n/a	Rf0 Ci1 CC1
[165]	2012	general	B	Po	n/a	n/a	n/a	n/a	Rf1 Ci1 CC1
[166]	2009	dermatology	B	We T	V Au	n/a	n/a	Me	Rf1 Ci1 CC1
[167]	2012	anatomy	B	We	T V Au I D	n/a	n/a	n/a	Rf2 Ci1 CC1
[168]	2008	cardiology	B	We	T I D An	S C Q I F	n/a	n/a	Rf1 Ci1 CC1
[169]	2011	emergency	E	We C	n/a	S C I	n/a	n/a	Rf0 Ci1 CC0
[170]	2010	paediatrics	E	We	n/a	n/a	n/a	n/a	Rf1 Ci1 CC1
[171]	2013	anaesthesia	E	We	n/a	S	n/a	n/a	Rf2 Ci1 CC0
[172]	2010	radiology	E	T	n/a	n/a	n/a	n/a	Rf0 Ci1 CC0
[173]	2005	cardiology	E	We	T V Au I D	I F	n/a	n/a	Rf0 Ci0 CC0
[174]	2003	general	E	We E	T Au I	C Q I F	I F D	n/a	Rf0 Ci1 CC1
[175]	2008	geriatrics	E	We	T V Au I	S C I F P	D	Me	Rf2 Ci1 CC0
[176]	2011	microbiology	B	We	T	Q I F	n/a	n/a	Rf1 Ci2 CC0
[177]	2004	radiology	E	We	T I D	Cb S Q I F	n/a	n/a	Rf0 Ci1 CC0
[178]	2013	nuclear medicine	E	We T	T V Au I D	V Q I F	I F	n/a	Rf0 Ci0 CC0
[179]	2012	urology	E	C	T I	n/a	n/a	n/a	Rf1 Ci0 CC0
[180]	2012	general surgery	E	We Po	T V Au I	n/a	n/a	n/a	Rf2 Ci1 CC1
[181]	2007	anatomy	B	We M	n/a	Q I	F D	n/a	Rf2 Ci2 CC1

Ref	Year	Subject	Teach. Method	Platform support	Media features	Interactive features	Accessibility features	Instructional design principles	Citations
[182]	2010	advanced life support	E	C	T V Au I D	S I	n/a	Me	Rf2 Ci1 CC1
[183]	2012	advanced life support	B	We	V Au	Q F	n/a	n/a	Rf2 Ci1 CC1
[184]	2011	neuroscience	B	We	n/a	Q	F	n/a	Rf2 Ci1 CC1
[185]	2006	occupational medicine	B	We	T V Au I D	C Q I F	n/a	Me	Rf1 Ci1 CC1
[186]	2013	primary	E	We	T I	Q	n/a	n/a	Rf0 Ci0 CC0
[187]	2010	physiology	B	We	T I	C Q I F	F	n/a	Rf2 Ci2 CC1
[188]	2012	anatomy	B	We	T I D	Q I F P	n/a	Ch Si Cn Se Mo Me Pe	Rf1 Ci1 CC1
[189]	2007	general surgery	E	We	T Au I D	n/a	n/a	Me V	Rf1 Ci1 CC1
[190]	2012	evidence based medicine	E	We	T	C I	F	n/a	Rf1 Ci0 CC0
[191]	2003	general	B	We	T V Au I D	Cb Q I F P	F D Ca	Se Me	Rf1 Ci2 CC1
[192]	2006	emergency	E	We	T I D An	S I F P	n/a	n/a	Rf1 Ci1 CC0
[193]	2012	radiology	E	We	D	n/a	n/a	n/a	Rf1 Ci1 CC1
[194]	2007	geriatrics	B	We	T V Au I	S Q F	n/a	Pr Me	Rf2 Ci2 CC0
[195]	2013	general surgery	E	We	T V Au I D An	Cb S Q I F P	n/a	Ch Si	Rf1 Ci0 CC0
[196]	2012	histology	B	We	T I D	I F	F	n/a	Rf2 Ci1 CC1
[197]	2008	anatomy	B	C	V	n/a	n/a	n/a	Rf2 Ci1 CC1
[198]	2006	informatics	B	We	T	Cb P	F	n/a	Rf1 Ci2 CC1
[199]	2012	radiology	E	We	T V Au I D	C	n/a	Ch Se	Rf1 Ci1 CC0

Ref	Year	Subject	Teach. Method	Platform support	Media features	Interactive features	Accessibility features	Instructional design principles	Citations
[200]	2011	legal medicine	B	We	T Au I D	S C I F P	n/a	n/a	Rf1 Ci1 CC1
[201]	2011	histology	E	We	T I	S I	n/a	Me	Rf1 Ci1 CC0
[202]	2013	emergency	B	We	T V Au I D An	S Q I F P	n/a	n/a	Rf1 Ci1 CC1
[203]	2011	physiology	B	We	T V Au I D	Q Cl I	I F D Ca	n/a	Rf0 Ci1 CC1
[204]	2013	radiology	E	We T	V Au	n/a	D	Se	Rf2 Ci1 CC1
[205]	2010	paediatrics	E	We	V	n/a	n/a	n/a	Rf1 Ci0 CC1
[206]	2004	anatomy	B	We	T I D	Cb C Q I	n/a	Cn Pr Se Me	Rf1 Ci2 CC1
[207]	2007	emergency	B	We	n/a	C	n/a	n/a	Rf0 Ci0 CC0
[208]	2011	primary	B	We M	T	C Q F	n/a	n/a	Rf2 Ci1 CC1
[209]	2004	general	E	We T	T V Au I	Cb C I F P	F	Me	Rf0 Ci0 CC1
[210]	2008	radiology	E	We	n/a	n/a	n/a	Se	Rf1 Ci1 CC1
[211]	2011	dermatology	B	We	T V Au I	Q I F	F	Me	Rf1 Ci1 CC0
[212]	2009	pathology	E	We M	T V Au I D An	Q I F	I F D Ca	n/a	Rf1 Ci0 CC1
[213]	2013	urology	B	We	V	n/a	n/a	n/a	Rf2 Ci0 CC0
[214]	2007	emergency	E	We	T I D	S I	n/a	Si Cn Me	Rf1 Ci1 CC1
[215]	2007	paediatrics radiology	E	We	T V I D	C Q I	n/a	Me	Rf2 Ci2 CC1
[216]	2010	neurosurgery	B	We	n/a	S	n/a	n/a	Rf1 Ci0 CC1
[217]	2012	rheumatology	B	We Pt	V	Q I F	D	Si Pr	Rf2 Ci0 CC1
[218]	2012	ophthalmology	E	We	V Au	n/a	n/a	n/a	Rf0 Ci0 CC1

Ref	Year	Subject	Teach. Method	Platform support	Media features	Interactive features	Accessibility features	Instructional design principles	Citations
[219]	2009	pathology	E	We	T I D	Cb C Q I F	F D Ca	n/a	Rf0 Ci1 CC0
[220]	2012	paediatrics	B	We	T V Au I	n/a	n/a	Me	Rf0 Ci2 CC1
[221]	2013	ophthalmology	B	We	T V Au I	V C I	n/a	n/a	Rf1 Ci1 CC0
[222]	2008	anatomy	B	We	T V Au I D	C Q I F	n/a	Me	Rf0 Ci1 CC1
[223]	2008	paediatrics	E	We	T An	Cb G Q	n/a	n/a	Rf1 Ci1 CC0
[224]	2004	radiology	E	E	T I	n/a	n/a	Cn Se Me	Rf1 Ci1 CC0
[225]	2009	radiology	E	We	T I D	C Q I F	n/a	n/a	Rf0 Ci1 CC0
[226]	2012	otolaryngology	E	We	T I D An	V I	n/a	n/a	Rf1 Ci1 CC0
[227]	2004	physiology	B	We A	T V Au I D An	Cb S Q Cl I F P	I F	Si Pr Se Me	Rf0 Ci2 CC1
[228]	2006	pathology	E	We	n/a	C Q I	n/a	n/a	Rf0 Ci1 CC0
[229]	2008	gastrointestinal	E	We	T V Au I D An	C Q I F P	n/a	Se Me	Rf1 Ci0 CC0
[230]	2011	advanced life support paediatrics	B	We	n/a	n/a	n/a	n/a	Rf1 Ci1 CC1
[231]	2011	evidence based medicine	E	We T	T V Au	Q I F	I	n/a	Rf2 Ci2 CC1
[232]	2011	emergency	B	n/a	n/a	n/a	n/a	n/a	Rf2 Ci1 CC1
[233]	2010	professionalism	B	We B Wi	T	Cb	F	n/a	Rf1 Ci2 CC1
[234]	2012	biochemistry	B	We	n/a	C Q F	F D	n/a	Rf2 Ci1 CC1
[235]	2010	psychiatry	B	n/a	n/a	C I	D	n/a	Rf0 Ci1 CC1
[236]	2013	nephrology	E	We	T D	I	n/a	Ch Si	Rf2 Ci1 CC1

Ref	Year	Subject	Teach. Method	Platform support	Media features	Interactive features	Accessibility features	Instructional design principles	Citations
[237]	2012	general surgery	E	Pt	n/a	n/a	n/a	n/a	Rf1 Ci1 CC0
[238]	2007	neurology	E	We M	n/a	Cb Q I	I F	n/a	Rf2 Ci1 CC1
[239]	2006	primary	E	We	T	C I	F	n/a	Rf2 Ci1 CC1
[240]	2003	psychology	E	We	T V	I F P	F	Me	Rf0 Ci2 CC1
[241]	2011	physiology	E	n/a	T D	S I	n/a	Me	Rf0 Ci0 CC0
[242]	2006	rheumatology	B	We	T I	S C Q I F	n/a	Me	Rf1 Ci1 CC1
[243]	2010	general	B	We Wi	T V Au I	Cb S Q I F P	n/a	Pr Me	Rf1 Ci2 CC1
[244]	2009	traditional medicine	E	We	n/a	C	F	n/a	Rf2 Ci1 CC1
[245]	2013	general	B	We	T V Au I D An	Cb C	I F	n/a	Rf2 Ci1 CC1
[246]	2006	orthopaedics	E	We	T Au I An	C Q I F	n/a	Cn	Rf2 Ci1 CC1
[247]	2010	orthopaedics	E	We	T V Au I D An	S V C I F P	n/a	n/a	Rf2 Ci1 CC1
[248]	2006	radiology	E	We	V I	n/a	n/a	n/a	Rf1 Ci1 CC0
[249]	2012	general surgery	B	We	T V Au I	V I P	n/a	n/a	Rf2 Ci1 CC1
[250]	2009	anaesthesia	E	We	T I D	C Q I F	n/a	Me	Rf2 Ci2 CC1
[251]	2006	radiology	B	We	T V Au I D An	C	n/a	Cn Me	Rf0 Ci1 CC0

Experiment variables

Ref	Year	Study type	Participant total	Educ.	Duration	Knowledge	Attitudes	Skills	Usage	MERSQI	Findings
[1]	2011	Pr	190	B	<3Mo	n/a	Fo Lb	n/a	n/a	n/a	A+

Ref	Year	Study type	Participant total	Educ.	Duration	Knowledge	Attitudes	Skills	Usage	MERSQI	Findings
[2]	2012	Pr Pp C	277	C	<3Mo	M Li F E	Li	n/a	n/a	11	K+ A+
[3]	2013	Pr	522	C	<3Mo	n/a	Li	A	Ti	6	A+ U˜S+
[4]	2012	Pr	130	B	<3Mo	n/a	Li	n/a	Po R A	8	A+ U+
[5]	2009	Pr	150	B	<3Mo	M	Li	n/a	n/a	10	K+ A+
[6]	2011	Pr R Mt Pp C	37	R	<3Mo	M Li	Li	n/a	A Ti	11	K+ A+ U+
[8]	2012	Pr Pp	40	S	<1Wk	n/a	Li	A P	n/a	10	A+ S+
[9]	2012	Pr R C	60	B	<1Wk	M Li	Li	n/a	n/a	10	K˜A+
[10]	2012	Pr R Pp C	204	B	<1Wk	n/a	Li Lb	n/a	n/a	11	A-
[11]	2010	Rt	200	B	>3Mo	M E	Li	n/a	A Ti	7	K˜A+ U+
[12]	2012	Pr	101	S	>3Mo	n/a	Li Tx	n/a	A	9	A+ U˜
[14]	2008	Pr Pp	166	n/a	<1Wk	Tx P Lb	Li Lb	n/a	Ti	11	K+ A+ U+
[15]	2010	Pr Pp	137	S	<3Mo	M	Li	n/a	n/a	8	K+ A+
[16]	2009	Pr R Pp C	148	B	<1Wk	M	Li	n/a	n/a	12.5	K+ A+
[17]	2013	Pr Pp C	158	B	<3Mo	n/a	Li	n/a	n/a	10	A+
[18]	2010	Pr R Pp C	49	C	>3Mo	n/a	n/a	A	n/a	11	S+
[18]	2010	Pr R Mt Pp C	216	C	>3Mo	E	n/a	A P	n/a	11	S+
[20]	2012	Pr	125	B	>3Mo	M E	Li	n/a	Ti	11	K+ A+ U+
[21]	2011	Pr Mt	963	B	>3Mo	n/a	Tx	n/a	T A	9	A+ U˜

Ref	Year	Study type	Participant total	Educ.	Duration	Knowledge	Attitudes	Skills	Usage	MERSQI	Findings
[22]	2009	Rt	1736	B	>3Mo	E	n/a	n/a	n/a	11.5	K+
[22]	2009	Pr	1736	B	can't tell	E	Li	n/a	n/a	10	K+ A+
[23]	2013	Pr R Pp C	55	B	<3Mo	M	n/a	n/a	n/a	11	K+
[24]	2004	Pr R Pp C	11	C	<3Mo	n/a	Li P Fo Lb	n/a	Ti	8	A+ U~
[25]	2013	Pr Pp	40	R	<1Wk	M	Li	n/a	n/a	9	K+ A+
[26]	2008	Pr C	49	n/a	<3Mo	Li P	Li	A P Lb	Po	11	K+ U~S~
[28]	2012	Pr Mt	10261	S	>3Mo	n/a	Li	n/a	n/a	9	A+
[29]	2011	Pr Mt	7405	R	>3Mo	n/a	Li	n/a	n/a	7	A+
[30]	2003	Pr R Mt Pp C	3067	S	<1Wk	M	n/a	n/a	n/a	9	K+
[31]	2012	Pr Pp C	167	C	<1Wk	M Lb	n/a	n/a	n/a	10	K~
[32]	2009	Pr	50	C	can't tell	n/a	Li	n/a	n/a	9	A+
[33]	2009	Pr R C	80	C	<1Wk	M	Li	n/a	n/a	11	A+
[34]	2012	Pr	30	C	can't tell	n/a	Li	n/a	n/a	9	A+
[35]	2008	Pr	90	C	<3Mo	n/a	Li Tx	n/a	Po R A	9	A+ U~
[36]	2009	Pr	309	n/a	<3Mo	n/a	Li	n/a	A	7.5	A+ U+
[37]	2009	Pr	10	B	<3Mo	n/a	n/a	n/a	n/a	7	A+
[38]	2012	Pr C	150	S	>3Mo	n/a	n/a	n/a	n/a	9	K+
[39]	2011	Pr C	12	B	<1Wk	M	Li	n/a	n/a	10	K~A+

Ref	Year	Study type	Participant total	Educ.	Duration	Knowledge	Attitudes	Skills	Usage	MERSQI	Findings
[40]	2012	Pr Mt	50	B	<3Mo	n/a	P	n/a	Po A	n/a	A+ U˜
[41]	2012	Pr Mt	60	C	<3Mo	n/a	Li Fo Lb	n/a	Po A	8.5	A+ U+
[42]	2008	Pr C	117	C	>3Mo	E	Li	n/a	A	10.5	K+ A+ U˜
[43]	2008	Pr	118	C	>3Mo	n/a	Li Tx	A P	Po Ti	7	A+ U˜S+
[44]	2012	Pr Pp C	30	B	>3Mo	tf Lb	n/a	A P Lb	n/a	10	K+ S+
[45]	2009	Pr	149	C	<3Mo	M Tx E Lb	Tx	n/a	T A Ti	10.5	A+ U+
[46]	2012	Pr	43	S	>3Mo	n/a	Li	n/a	n/a	7	A+ U+
[47]	2013	Pr Mt C	562	C	<3Mo	M tf E Lb	Li	n/a	n/a	9	K+ A+
[48]	2011	Pr	70	S	<1Wk	n/a	Li Lb	n/a	n/a	10	A+
[49]	2011	Pr Pp	300	n/a	>3Mo	Li	Li	n/a	n/a	10.5	K+ A+
[50]	2012	Pr	387	B C	>3Mo	n/a	Li Fo	n/a	A	9	A+ U˜
[51]	2007	Pr R Mt Pp C	229	R	<1Wk	M O P Lb	Li Lb	n/a	n/a	12.5	K˜A+
[52]	2012	Pr C	128	S	<1Wk	n/a	n/a	A P Lb	n/a	12	S+
[53]	2012	Pr	106	B	>3Mo	n/a	Li	n/a	n/a	6	A+
[54]	2006	Pr	355	B	<3Mo	n/a	Li	n/a	n/a	6	A+
[55]	2007	Pr	21	B	>3Mo	n/a	Fo	n/a	n/a	6	A+
[57]	2011	Pr Pp	20	R	>3Mo	n/a	n/a	n/a	Po R	8	K˜U˜
[59]	2010	Pr	246	B	<3Mo	Li	Li	n/a	n/a	10	K+ A+
[61]	2012	Pr Pp	137	B	<3Mo	M Lb	Li	n/a	n/a	6	K+ A+

Ref	Year	Study type	Participant total	Educ.	Duration	Knowledge	Attitudes	Skills	Usage	MERSQI	Findings
[63]	2008	Rt Mt C	612	R	>3Mo	M	n/a	n/a	n/a	6	K+
[64]	2003	Pr	can't tell	B	can't tell	n/a	n/a	n/a	T A	10.5	A+ U+
[65]	2005	Pr	13	C	<1Wk	n/a	Li Tx Lb	n/a	n/a	8	A+
[66]	2013	Pr Pp	21	B R	<3Mo	M Li	Li	n/a	n/a	8	K+ A+
[68]	2009	Pr R Pp C	102	B	<3Mo	n/a	n/a	A P Lb	n/a	11	S+
[69]	2013	Pr Mt Pp	185	R	<1Wk	M Li	n/a	n/a	A Ti	11	K+ U˜
[70]	2005	Pr Pp	30	B C R	<3Mo	M E	Li Tx	n/a	n/a	8	K+ A+
[71]	2006	Pr R Pp C	126	B C	<1Wk	M Lb	Li Tx	A P Lb	Ti	9	K˜A+ U+ S+
[72]	2012	Pr	116	C	<3Mo	M F	Li	n/a	n/a	7	K+ A+
[73]	2006	Pr	6	C	<3Mo	n/a	Li Fo	n/a	Po R A Ti	6	A+ U˜
[74]	2013	Pr	26	B	>3Mo	n/a	Li	n/a	n/a	9	A+
[77]	2012	Rt C	36	B	<3Mo	E	Li	n/a	n/a	10	K+ A+
[78]	2013	Pr	112	C	<3Mo	n/a	Li	n/a	n/a	8	A+
[79]	2011	Pr Pp C	223	R	<3Mo	M	Li	n/a	n/a	11	K+ A+
[80]	2011	Pr	260	C	<3Mo	n/a	Li	n/a	n/a	10	A+
[81]	2006	Pr	276	B	<3Mo	M	Li Tx	n/a	T Po R A Ti	10.5	K+ A+ U˜
[82]	2008	Pr Mt	62	B S	<1Wk	n/a	Li	n/a	n/a	8	A+
[83]	2012	Pr Pp	245	C	<1Wk	M	Li	n/a	Ti	11	K+ A+ U˜

Ref	Year	Study type	Participant total	Educ.	Duration	Knowledge	Attitudes	Skills	Usage	MERSQI	Findings
[84]	2010	Pr Pp C	88	C	<3Mo	n/a	Li	A P	Po R A Ti	10	A+ U˜S˜
[85]	2010	Pr R Pp C	133	B	<3Mo	M Li tf Tx Lb	Li Lb	A P Lb	n/a	12.5	K+ A+ S+
[86]	2007	Pr	93	n/a	can't tell	n/a	Li	n/a	A	6	A+ U˜
[87]	2013	Pr Mt	54	R	<1Wk	Li	Li	n/a	Ti	10	A+ U+
[89]	2012	Pr R C	155	B	<1Wk	M E	n/a	n/a	Po Ti	11	K- U-
[90]	2013	Pr R C	97	R	<3Mo	M	Li	n/a	n/a	12	K+ A+
[91]	2010	Pr R Mt Pp C	237	B	<3Mo	M E	n/a	n/a	n/a	12	K˜
[92]	2011	Pr	304	B	<3Mo	n/a	Li	n/a	n/a	7	A˜
[94]	2012	Pr R Pp C	58	R	<1Wk	n/a	n/a	A P	n/a	11	S˜
[95]	2010	Pr	10	R	<3Mo	n/a	Li	n/a	Ti	9	A+ U˜
[96]	2008	Pr	82	B	>3Mo	n/a	Li Tx	n/a	n/a	6	A˜
[97]	2013	Pr R Mt C	120	S	<1Wk	M	n/a	n/a	Ti	11	K˜U˜
[98]	2013	Pr	341	B	<3Mo	n/a	Li	n/a	n/a	9	A+
[99]	2008	Pr	302	C	<3Mo	Tx P Lb	Li Tx Fo Lb	n/a	n/a	10.5	K+ A+
[100]	2012	Pr R Pp C	166	B	<3Mo	n/a	n/a	A P	n/a	13	S+
[101]	2009	Pr C	92	C	<3Mo	M E	n/a	A P Lb	n/a	10.5	K˜S+
[103]	2010	Rt	can't tell	n/a	can't tell	E	n/a	n/a	n/a	9	K+ A+
[104]	2013	Pr	62	n/a	<3Mo	n/a	Li	n/a	n/a	7	A+

Ref	Year	Study type	Participant total	Educ.	Duration	Knowledge	Attitudes	Skills	Usage	MERSQI	Findings
[106]	2009	Pr R Pp C	92	B	<1Wk	M	n/a	n/a	n/a	11	K+
[107]	2009	Pr	83	C	can't tell	n/a	Li Tx P Fo Lb	n/a	n/a	8	A+
[110]	2010	Pr R C	100	B C	<1Wk	M Lb	Li Lb	n/a	n/a	11	K˜A+
[111]	2013	Pr C	116	C	>3Mo	n/a	Fo	n/a	n/a	11	A+
[112]	2009	Pr	can't tell	n/a	can't tell	n/a	n/a	n/a	A	n/a	U+
[113]	2011	Pr R Pp C	16	R	>3Mo	M	Li	n/a	n/a	11	K˜A+
[114]	2012	Pr	91	B	>3Mo	n/a	Li	n/a	A Ti	7	A+ U˜
[115]	2012	Pr	84	B	<3Mo	n/a	Li	n/a	n/a	9	A˜
[116]	2009	Pr	42	B	<3Mo	M	Li	n/a	n/a	9	K˜A+
[117]	2009	Pr R Pp C	133	B	<1Wk	M	Li	n/a	Ti	11	K+ A+ U+
[118]	2008	Pr R C	237	B	<3Mo	M E Lb	Tx	n/a	n/a	12.5	K˜A+
[119]	2009	Pr R Mt C	537	R	>3Mo	M	n/a	n/a	n/a	12	K+
[120]	2012	Pr R Mt C	1470	R	>3Mo	M	Li	n/a	n/a	11	K+ A+
[121]	2007	Pr R Mt Pp C	537	R	>3Mo	M	n/a	n/a	Ti	10	K+ U˜
[122]	2009	Pr R Mt Pp C	330	B	>3Mo	M Li Tx E Lb	Li	n/a	n/a	12	K+
[123]	2007	Pr R Pp C	133	B	>3Mo	M Lb	Li Tx	n/a	n/a	12	K+
[124]	2010	Pr R Mt Pp C	724	R	>3Mo	M	n/a	n/a	n/a	12.5	K+ A+
[125]	2011	Pr	164	B	<3Mo	n/a	Li Tx Lb	n/a	Ti	7.5	A+

Ref	Year	Study type	Participant total	Educ.	Duration	Knowledge	Attitudes	Skills	Usage	MERSQI	Findings
[126]	2013	Pr R C	60	B	<1Wk	M Lb	n/a	n/a	n/a	11	K-
[127]	2010	Pr Mt	153	S	can't tell	n/a	Li	n/a	T	9	A+ U˜
[130]	2006	Pr Mt	212	B	<3Mo	n/a	Li	n/a	n/a	9	A+
[132]	2012	Pr R Pp C	226	B	<3Mo	tf F Lb	Li	A P Lb	T A Ti	12	K+ A+ U+ S+
[133]	2005	Pr	150	B C	<3Mo	M E	Li	n/a	T	7	K+ A+ U+
[134]	2010	Pr R C	295	B	<1Wk	M O Tx F	Li	n/a	n/a	10	K+ A+
[135]	2008	Pr Mt Pp	68	B	<1Wk	n/a	Li	n/a	T A	11.5	K+ A+ U˜
[135]	2008	Pr Mt Pp	68	S	can't tell	n/a	Li	n/a	n/a	8	K+ A+
[136]	2008	Pr Mt Pp	112	S	<1Wk	M	n/a	n/a	n/a	6	K+ A+
[137]	2013	Rt Pp	121	C	<3Mo	M	Li	n/a	n/a	8	K+ A+
[138]	2010	Pr	30	C	can't tell	n/a	Li Tx	n/a	n/a	8	A+
[139]	2013	Pr	310	C	<3Mo	Li	Li Tx	A	n/a	8	K+ A+ S+
[140]	2012	Pr Pp C	127	C	<3Mo	Lb	Li Lb	n/a	A	11	K+ A+ U+
[141]	2011	Pr	140	C	>3Mo	n/a	Li	n/a	A	6	A+ U˜
[142]	2009	Pr C	41	B	<3Mo	M E	Li	n/a	Ti	9	K˜A+ U+
[143]	2006	Pr	209	B	<1Wk	n/a	Li	n/a	n/a	6	A+
[144]	2005	Pr	134	B	<1Wk	n/a	Li	n/a	n/a	8	A+
[145]	2006	Pr	1232	B	<1Wk	n/a	Li Lb	n/a	n/a	8	A˜

Ref	Year	Study type	Participant total	Educ.	Duration	Knowledge	Attitudes	Skills	Usage	MERSQI	Findings
[146]	2011	Pr Pp	272	S	<3Mo	M	Tx	n/a	n/a	8	K+ A+
[147]	2010	Pr R Mt C	42	S	<1Wk	M Lb	Li	n/a	Ti	9	K˜A˜U˜
[148]	2009	Pr	102	S	<1Wk	n/a	Li	n/a	n/a	9	A+
[149]	2010	Pr	22	C	can’t tell	n/a	Li	n/a	n/a	9	A+
[150]	2013	Pr R C	207	C	<3Mo	M F E Lb	n/a	n/a	T A	9	K˜U+
[151]	2008	Pr	141	B	>3Mo	n/a	Li Tx	n/a	n/a	7.5	A+
[152]	2011	Pr Pp	20	C R	<1Wk	M	n/a	n/a	n/a	9	K+
[153]	2011	Pr Pp	177	R	<3Mo	M	n/a	n/a	n/a	10	K+
[154]	2005	Pr Pp	50	B	<3Mo	n/a	Li	n/a	n/a	9	A˜
[155]	2009	Pr R C	55	R	>3Mo	n/a	Li	n/a	n/a	11	n/a
[156]	2013	Pr Pp	82	R	<1Wk	M	Li	n/a	n/a	10	K+ A+
[157]	2010	Pr	can’t tell	n/a	<1Wk	n/a	n/a	n/a	R A Ti	n/a	U˜
[158]	2012	Pr	52	C S	can’t tell	n/a	Li	n/a	n/a	8	A+
[159]	2009	Pr R Pp C	72	R	<3Mo	M	Li	A	R A	12	K+ A+ U˜S+
[161]	2010	Pr R Pp C	237	n/a	<3Mo	M Li Lb	Li P Fo	n/a	n/a	12.5	K+
[162]	2010	Pr R C	143	n/a	<1Wk	n/a	Li Lb	n/a	n/a	10	A+
[163]	2012	Pr R Pp C	520	C	<3Mo	M Lb	Li Tx Lb	n/a	Ti	11.5	K+ A+ U+
[164]	2010	Pr Pp	38	B	>3Mo	M E Lb	Li Lb	n/a	n/a	10.5	K+ A+

Ref	Year	Study type	Participant total	Educ.	Duration	Knowledge	Attitudes	Skills	Usage	MERSQI	Findings
[165]	2012	Pr R Mt C	70	C	<3Mo	n/a	Li	n/a	n/a	10	A+
[166]	2009	Pr	325	n/a	>3Mo	n/a	Li	n/a	n/a	8	A+
[167]	2012	Pr	804	B	>3Mo	n/a	n/a	n/a	A Ti	9	U+
[168]	2008	Pr C	62	B	>3Mo	M E	Li	n/a	A	8	K+ A+ U˜
[169]	2011	Pr Pp	93	S	<3Mo	M Li	Li	n/a	n/a	12	K+ A+
[170]	2010	Pr Pp	28	B	<3Mo	M Li Lb	Tx	A P Lb	n/a	11	K+ A+ S+
[171]	2013	Pr R C	20	B	<1Wk	n/a	n/a	A	n/a	11	S+
[172]	2010	Pr	18	R	>3Mo	n/a	Li Tx	n/a	n/a	10	A+
[175]	2008	Pr Mt	287	C	can't tell	n/a	Li	n/a	Ti	8	U˜
[176]	2011	Pr Pp	307	B	<3Mo	M	Li	n/a	n/a	10	K+ A+
[177]	2004	Pr	17	R	can't tell	n/a	Li Lb	n/a	n/a	6	A+
[179]	2012	Pr Pp	10	S	<1Wk	M Li	Li	n/a	n/a	8	K+ A+
[180]	2012	Pr R Pp C	154	C	<1Wk	M Li	Li	n/a	n/a	11	K˜A-
[181]	2007	Pr C	134	B	>3Mo	M E	Li	n/a	A	8	K+ A+ U+
[182]	2010	Pr R Mt Pp C	657	S	can't tell	M E	Li	A P Lb	n/a	13.5	K˜A+ S˜
[183]	2012	Pr R Mt Pp C	3732	S	<1Wk	M E	n/a	A P	n/a	12	K- S-
[184]	2011	Pr Pp	73	B	<1Wk	M O E Lb	Li	n/a	n/a	11	K+ A+
[185]	2006	Pr Mt	557	C	>3Mo	M	Li	n/a	T Ti	8	K+ A+ U˜

Ref	Year	Study type	Participant total	Educ.	Duration	Knowledge	Attitudes	Skills	Usage	MERSQI	Findings
[186]	2013	Pr	280	S	can't tell	Li	n/a	n/a	n/a	8	K+
[187]	2010	Pr R C	183	C	<3Mo	M Li	n/a	n/a	n/a	11	K+
[188]	2012	Pr Pp	129	B	>3Mo	M O Tx E Lb	Li Tx	n/a	A	7	K˜A+ U˜
[189]	2007	Pr C	88	C	<3Mo	M E Lb	Li	n/a	A	10	K+ A+ U˜
[190]	2012	Pr	61	R	<1Wk	n/a	Li Fo	n/a	n/a	8	A+
[192]	2006	Pr Pp C	29	S	<1Wk	M F E	n/a	n/a	n/a	8	K+ U+
[193]	2012	Pr Mt C	80	B S	<1Wk	n/a	Li Lb	A P Lb	Ti	10	A+ U˜S+
[194]	2007	Pr Pp C	140	B	can't tell	M Li	Li P Fo Lb	A P Lb	n/a	10.5	K+ A+ S+
[195]	2013	Pr	10	S	<1Wk	n/a	Li Tx	n/a	n/a	10	A+
[196]	2012	Pr Pp	89	B	>3Mo	n/a	Li	n/a	A	7	A+ U+
[197]	2008	Pr	282	B	<3Mo	M E	Li	n/a	A	11	K˜A+ U˜
[198]	2006	Pr R Pp C	238	B	<3Mo	Li O Tx F E P	Li	A P Lb	n/a	12.5	K+ A+ S+
[200]	2011	Pr Pp	36	B	<3Mo	n/a	Li	n/a	n/a	8.5	A+
[201]	2011	Pr	447	B	<3Mo	n/a	Li	n/a	A	8	A+ U+
[203]	2011	Rt C	104	B	>3Mo	M E	Li	n/a	T Po R A Ti	10	K+ A+ U+
[204]	2013	Pr C	191	B	<1Wk	P	Li	A P	n/a	10	K+ A+ S+
[205]	2010	Pr	100	C	can't tell	n/a	n/a	n/a	A	9	A+
[206]	2004	Pr	508	B	>3Mo	n/a	Li Tx	n/a	n/a	8.5	A+
[207]	2007	Pr	210	C	<3Mo	n/a	Li	n/a	n/a	n/a	A+

Ref	Year	Study type	Participant total	Educ.	Duration	Knowledge	Attitudes	Skills	Usage	MERSQI	Findings
[208]	2011	Pr R C	300	S	>3Mo	n/a	Li	n/a	n/a	14	A+
[210]	2008	Pr	62	B	<3Mo	n/a	Li	n/a	n/a	6	A+
[211]	2011	Pr R Pp C	44	B	<3Mo	M	Li	n/a	n/a	12.5	K+
[212]	2009	Pr	38	C	<3Mo	n/a	Li	n/a	Po R A	9	A+ U+
[213]	2013	Pr Pp	7	R	<1Wk	Li	n/a	A P	n/a	7	K+ S+
[214]	2007	Pr Pp	41	C	<1Wk	M Lb	Tx Lb	n/a	Ti	10.5	K+ A+ U+
[215]	2007	Pr	can't tell	n/a	can't tell	n/a	n/a	n/a	Po A Ti	n/a	U˜
[216]	2010	Pr R C	65	C R	<1Wk	n/a	Li Lb	A P Lb	n/a	11	A- S-
[217]	2012	Rt C	18	C	<3Mo	M E	Li Fo	n/a	A	9	K˜A+ U+
[218]	2012	Pr R C	25	B	<1Wk	M	Li	n/a	Ti	11	K+ A+ U+
[219]	2009	Pr	14	S	<3Mo	n/a	Li Tx Fo Lb	n/a	n/a	7	A+
[220]	2012	Pr R C	81	C	<3Mo	n/a	Li	A P Lb	n/a	12.5	A+ S+
[221]	2013	Pr R Mt Pp C	188	C	<3Mo	M	Li	n/a	n/a	11	K+ A+
[222]	2008	Pr	205	B	<3Mo	M E Lb	Li Lb	n/a	n/a	10.5	K˜A˜
[223]	2008	Pr R Pp C	108	B	<3Mo	M	Li Tx	n/a	n/a	11	K˜
[226]	2012	Pr R C	40	R	<1Wk	M Lb	Li	n/a	n/a	12	K˜A+
[227]	2004	Pr C	121	B	<3Mo	Lb	Li Lb	n/a	T	11.5	K+ A+ U+
[228]	2006	Pr	can't tell	n/a	can't tell	n/a	n/a	n/a	n/a	n/a	A+

Ref	Year	Study type	Participant total	Educ.	Duration	Knowledge	Attitudes	Skills	Usage	MERSQI	Findings
[229]	2008	Pr	200	B	<3Mo	n/a	n/a	n/a	n/a	9	A+
[230]	2011	Pr Pp	21	B C	can't tell	M Li	Li Tx	n/a	n/a	10.5	K+ A+
[231]	2011	Pr	176	S	>3Mo	M F E	n/a	n/a	A	9	K+ U~
[232]	2011	Pr R Pp C	19	C	<3Mo	O E P Lb	Li Tx	n/a	n/a	12.5	K+ A+
[233]	2010	Pr	32	B	<3Mo	n/a	Li P Fo Lb	n/a	n/a	8	A+
[234]	2012	Pr	60	B	>3Mo	F E	Li Tx	n/a	A	11	K~A+
[235]	2010	Pr R Pp C	389	S	<3Mo	M	Li	n/a	T	11	K~A+ U+
[236]	2013	Pr Pp	35	C	<3Mo	M Li	Li	A P	T	8	K+ A+ S+
[237]	2012	Pr	40	R	>3Mo	n/a	Li Fo	n/a	n/a	10	A+
[238]	2007	Pr Pp C	41	S	>3Mo	M E	Li	n/a	Ti	8	K+ A+ U~
[239]	2006	Pr R Pp C	159	B	>3Mo	M Tx	Li	n/a	Ti	9	K+ A~U~
[240]	2003	Pr Mt Pp	10	C	<3Mo	Li O P	Li P Lb	n/a	T Po R A	13.5	K+ A+ U+
[241]	2011	Pr	310	n/a	can't tell	n/a	n/a	n/a	T	n/a	U~
[242]	2006	Pr	84	B R	can't tell	M O P	Li Tx	n/a	n/a	n/a	K+ A+
[243]	2010	Pr Pp C	185	B	<3Mo	M E	Li Tx P Lb	n/a	T A	10.5	A+ U+
[244]	2009	Pr R C	1267	S	<3Mo	M Li	n/a	n/a	T Po R A Ti	12	K+ U+
[245]	2013	Pr R Pp C	120	B C	<1Wk	M tf	Li	n/a	n/a	11	K+ A+
[246]	2006	Pr	18	n/a	can't tell	n/a	Li	n/a	n/a	6	A+

Ref	Year	Study type	Participant total	Educ.	Duration	Knowledge	Attitudes	Skills	Usage	MERSQI	Findings
[247]	2010	Pr Pp	160	C	<1Wk	M	Li Tx	n/a	A Ti	8	K+ A+ U˜
[249]	2012	Pr C	99	B	<3Mo	Li	Li	A P Lb	n/a	10	A+ S˜
[250]	2009	Pr Mt Pp	454	S	can't tell	n/a	n/a	n/a	n/a	9	n/a

Experimental groups for controlled trials

Ref	Year	Controls
[2]	2012	B-learning vs. Lecture
[6]	2011	B-learning vs. Lecture
[9]	2012	Exploratory practice vs. Blocked practice
[10]	2012	Virtual Patient vs. OSCE vs. Role-play
[16]	2009	E-learning vs. Lecture
[17]	2013	B-learning vs. Lecture
[18]	2010	Simulation vs. Lecture
[18]	2010	Virtual patient + lecture vs. Virtual patient vs. Lecture
[23]	2013	Multimedia content vs. Text content
[24]	2004	Complex user interface vs. Simple user interface
[26]	2008	B-learning vs. Lecture
[30]	2003	B-learning vs. No intervention
[31]	2012	E-learning vs. Lecture

Ref	Year	Controls
[33]	2009	Adaptive system vs. Non-adaptive system
[38]	2012	E-learning vs. Lecture
[39]	2011	Simulation vs. Dissection + Simulation vs. No intervention
[42]	2008	B-learning vs. Lecture
[44]	2012	Immediate feedback vs. Delayed feedback
[47]	2013	B-learning vs. Lecture
[51]	2007	E-learning vs. Lecture
[52]	2012	E-learning vs. No intervention
[63]	2008	B-learning vs. Lecture
[68]	2009	Simulation vs. No Intervention
[71]	2006	Digital multimedia content vs. Printed content
[77]	2012	B-learning vs. Lecture
[79]	2011	E-learning vs. No intervention
[84]	2010	E-learning vs. Lecture
[85]	2010	E-learning vs. Lecture
[89]	2012	Timed virtual patient vs. Untimed virtual patient
[90]	2013	Spaced education vs. No intervention
[91]	2010	E-learning vs. Lecture
[94]	2012	Lecture notes vs. Lecture + Group discussion

Ref	Year	Controls
[97]	2013	Interactive virtual patient vs. Audio virtual patient vs. No intervention
[100]	2012	Immediate feedback vs. Delayed feedback
[101]	2009	B-learning vs. Lecture
[106]	2009	Simulation + Explanation vs. Simulation vs. Multimedia content
[110]	2010	3d content vs. 2d content
[111]	2013	Different sequences of Lecture, Discussion and Virtual patient activities
[113]	2011	E-learning vs. No intervention
[117]	2009	Clinical cases vs. Research articles
[118]	2008	Spaced education vs. Bolus education
[119]	2009	Spaced education vs. Bolus education
[120]	2012	Two-week spaced education vs. Four-week spaced education
[121]	2007	Spaced education vs. Bolus education
[122]	2009	Spaced education vs. Bolus education
[123]	2007	Spaced education vs. No intervention
[124]	2010	Spaced education vs. Bolus education
[126]	2013	3d content vs. 2d content vs. Physical model
[132]	2012	B-learning vs. Lecture
[134]	2010	Simulation vs. Laboratory vs. No intervention

Ref	Year	Controls
[140]	2012	In-person e-learning session vs. Distant e-learning session
[142]	2009	B-learning vs. Lecture
[147]	2010	In-person e-learning session vs. Distant e-learning session
[150]	2013	Spaced education vs. Bolus education
[153]	2011	E-learning vs. No intervention
[155]	2009	Spaced education vs. No intervention
[159]	2009	B-learning vs. Lecture
[161]	2010	Wiki vs. Instant messaging vs. Links to external resources
[162]	2010	B-learning vs. Lecture
[163]	2012	Integrated web-application vs. Online resources
[165]	2012	E-learning vs. No intervention
[168]	2008	B-learning vs. Lecture
[171]	2013	E-learning vs. No intervention
[180]	2012	E-learning vs. Lecture
[181]	2007	B-learning vs. Lecture
[182]	2010	B-learning vs. Lecture
[183]	2012	B-learning vs. Lecture

Ref	Year	Controls
[187]	2010	B-learning vs. Lecture
[189]	2007	Multimedia content vs. Text content
[192]	2006	Adaptive system vs. Non-adaptive system
[193]	2012	3d content vs. 2d content
[194]	2007	B-learning vs. Lecture
[198]	2006	B-learning vs. Lecture
[203]	2011	B-learning vs. Lecture
[204]	2013	E-learning vs. Lecture
[208]	2011	Immediate feedback vs. Delayed feedback
[211]	2011	E-learning vs. Lecture
[216]	2010	3d content vs. 2d content vs. Physical model
[217]	2012	B-learning vs. Lecture
[218]	2012	Multimedia content vs. Text content
[220]	2012	B-learning vs. Lecture
[221]	2013	B-learning vs. Lecture
[223]	2008	Exploratory practice vs. Blocked practice
[226]	2012	3d content vs. 2d content

Ref	Year	Controls
[227]	2004	B-learning vs. Lecture
[232]	2011	Spaced education vs. Bolus education
[235]	2010	B-learning vs. Lecture
[238]	2007	E-learning vs. No intervention
[239]	2006	E-learning vs. Lecture
[243]	2010	B-learning vs. Lecture
[244]	2009	Many hyperlinks vs. Few hyperlinks, Spaced education vs. Bolus education
[245]	2013	Complex user interface vs. Simple user interface
[249]	2012	Virtual patient vs. No intervention

References

[1] Laura E Abate, Alexandra Gomes, and Anne Linton. Engaging students in active learning: Use of a blog and audience response system. Medical reference services quarterly, 30(1):12–18, 2011.

[2] Rehab Abdelhai, Sahar Yassin, Mohamad F Ahmad, and Uno GH Fors. An e-learning reproductive health module to support improved student learning and interaction: a prospective interventional study at a medical school in egypt. BMC medical education, 12(1):11, 2012.

[3] Martin Abendroth, Sigrid Harendza, and Martin Riemer. Clinical decision making: a pilot e-learning study. The clinical teacher, 10(1):51–55, 2013.

[4] Ahmad S Alamro and Susie Schofield. Supporting traditional pbl with online discussion forums: A study from qassim medical school. Medical teacher, 34(s1):S20–S24, 2012.

[5] Cara J Alexander, Weronika M Crescini, Justin E Juskewitch, Nirusha Lachman, and Wojciech Pawlina. Assessing the integration of audience response system technology in teaching of anatomical sciences. Anatomical sciences education, 2(4):160–166, 2009.

[6] Joanne Alfieri, Lorraine Portelance, Luis Souhami, Yvonne Steinert, Peter McLeod, Fleure Gallant, and Giovanni Artho. Development and impact evaluation of an e-learning radiation oncology module. International Journal of Radiation Oncology* Biology* Physics, 82(3):e573–e580, 2012.

[7] Ali Alikhan, Ravneet R Kaur, and Steven R Feldman. Podcasting in dermatology education. Journal of dermatological treatment, 21(2):73–79, 2010.

[8] Allen D Andrade, Pedro Cifuentes, Michael J Mintzer, Bernard A Roos, Ramanakumar Anam, and Jorge G Ruiz. Simulating geriatric home safety assessments in a three-dimensional virtual world. Gerontology & geriatrics education, 33(3):233–252, 2012.

[9] Fernando Aparicio, Manuel De Buenaga, Margarita Rubio, and Asunción Hernando. An intelligent information access system assisting a case based learning methodology evaluated in higher education with medical students. Computers & Education, 58(4):1282–1295, 2012.

[10] Leen Aper, Jan Reniers, Sebastiaan Koole, Martin Valcke, and Anselme Derese. Impact of three alternative consultation training formats on self-efficacy and consultation skills of medical students. Medical teacher, 34(7):e500–e507, 2012.

[11] Thierry RH Bacro, Mulugeta Gebregziabher, and Timothy P Fitzharris. Evaluation of a lecture recording system in a medical curriculum. Anatomical sciences education, 3(6):300–308, 2010.

[12] David P Bahner, Eric Adkins, Nilesh Patel, Chad Donley, Rollin Nagel, and Nicholas E Kman. How we use social media to supplement a novel curriculum in medical education. Medical teacher, 34(6):439–444, 2012.

[13] PD Bamidis, S Konstantinidis, CL Papadelis, E Perantoni, C Styliadis, C Kourtidou-Papadeli, C Kourtidou-Papadeli, and C Pappas. An e-learning platform for aerospace medicine. Hippokratia, 12(Suppl 1):15, 2008.

[14] Johannes Bernhardt, Florian Hye, Sigrid Thallinger, Pamela Bauer, Gabriele Ginter, and Josef Smolle. Simulation of a mycological koh preparation–e-learning as a practical dermatologic exercise in an undergraduate medical curriculum. JDDG: Journal der Deutschen Dermatologischen Gesellschaft, 7(7):597–601, 2009.

[15] Julien Beynat, A Ben Mehidi, J-P Aubert, A-M Bron, P Massin, and Catherine Creuzot-Garcher. Retidiab®: Assessment of a continuing medical education website for the improvement of diabetic retinopathy management. Diabetes & metabolism, 37(2):118–123, 2011.

[16] I Bhatti, K Jones, L Richardson, D Foreman, J Lund, and G Tierney. E-learning vs lecture: which is the best approach to surgical teaching? Colorectal Disease, 13(4):459–462, 2011.

[17] Majid Zare Bidaki, Ali Rajabpour Sanati, and Fateme Rajai Ghannad. Producing and introducing mobile books, as a new model of providing learning content in medical sciences. Procedia-Social and Behavioral Sciences, 83:99–102, 2013.

[18] Mihaela Botezatu, Haakan Hult, Mesfin Kassaye Tessma, and Uno Fors. Virtual patient simulation: Knowledge gain or knowledge loss? Medical teacher, 32(7):562–568, 2010.

[19] Maged N Kamel Boulos, Lee Hetherington, and Steve Wheeler. Second life: an overview of the potential of 3-d virtual worlds in medical and health education. Health Information & Libraries Journal, 24(4):233–245, 2007.

[20] Sondre Boye, Torolf Moen, and Torstein Vik. An e-learning course in medical immunology: Does it improve learning outcome? Medical teacher, 34(9):e649–e653, 2012.

[21] Nicola Brennan and Karen Mattick. Exploring the map of medicine's potential in undergraduate medical education. Medical teacher, 33(8):e454–e460, 2011.

[22] Patrick D Bridge, Matt Jackson, and Leah Robinson. The effectiveness of streaming video on medical student learning: A case study. Medical education online, 14, 2009.

[23] SS Bruce-Low, S Burnet, K Arber, D Price, L Webster, and M Stopforth. Interactive mobile learning: a pilot study of a new approach for sport science and medical undergraduate students. Advances in physiology education, 37(4):292–297, 2013.

[24] Jean Marc Brunetaud, Nicolas Leroy, Sylvia Pelayo, Caroline Wascat, Jean Marie Renard, Lionel Prin, and Marie Catherine Beuscart-Zéphir. Comparative evaluation of two applications for delivering a multimedia medical course in the french-speaking virtual medical university (umvf). International journal of medical informatics, 74(2):209–212, 2005.

[25] Fabio Buttussi, Tommaso Pellis, Alberto Cabas Vidani, Daniele Pausler, Elio Carchietti, and Luca Chittaro. Evaluation of a 3d serious game for advanced life support retraining. International journal of medical informatics, 82(9):798–809, 2013.

[26] Mike Carbonaro, Sharla King, Elizabeth Taylor, Franziska Satzinger, Fern Snart, and Jane Drummond. Integration of e-learning technologies in an interprofessional health science course. Medical Teacher, 30(1):25–33, 2008.

[27] Carol Carraccio and Robert Englander. Analyses/literature reviews: evaluating competence using a portfolio: a literature review and web-based application to the acgme competencies. Teaching and learning in medicine, 16(4):381–387, 2004.

[28] Alessandro Carriero, Lorenzo Bonomo, Fabrizio Calliada, PAOLO Campioni, CESARE Colosimo, Antonio Cotroneo, M Cova, Giovanni Carlo Ettorre, Carlo Fugazzola, Giacomo Garlaschi, et al. E-learning in radiology: an italian multicentre experience. European journal of radiology, 81(12):3936–3941, 2012.

[29] Alessandro Carriero, B Beomonte Zobel, Lorenzo Bonomo, G Meloni, Antonio Cotroneo, M Cova, Giovanni Carlo Ettorre, Carlo Fugazzola, Giacomo Garlaschi, Luca Macarini, et al. E-learning in radiology: Italian multicentre experience. La radiologia medica, 116(7):989–999, 2011.

[30] Linda L Casebeer, Sheryl M Strasser, Claire M Spettell, Terry C Wall, Norman Weissman, Midge N Ray, and Jeroan J Allison. Designing tailored web-based instruction to improve practicing physicians' preventive practices. Journal of Medical Internet Research, 5(3), 2003.

[31] Serena H Chao, Belle Brett, John M Wiecha, Lisa E Norton, and Sharon A Levine. Use of an online curriculum to teach delirium to fourth-year medical students: A comparison with lecture format. Journal of the American Geriatrics Society, 60(7):1328–1332, 2012.

[32] Lih-Shyang Chen, Yuh-Ming Cheng, Sheng-Feng Weng, Yong-Guo Chen, and Chyi-Her Lin. Applications of a time sequence mechanism in the simulation cases of a web-based medical problem-based learning system. Educational Technology & Society, 12(1):149, 2009.

[33] Yuh-Ming Cheng, Lih-Shyang Chen, Hui-Chung Huang, Sheng-Feng Weng, Yong-Guo Chen, and Chyi-Her Lin. Building a general purpose pedagogical agent in a web-based multimedia clinical simulation system for medical education. Learning Technologies, IEEE Transactions on, 2(3):216–225, 2009.

[34] Yuh-Ming Cheng, Sheng-Huang Kuo, Shi-Jer Lou, and Ru-Chu Shih. The effect of applying online pbl case system to multiple disciplines of medical education. Turkish Online Journal of Educational Technology-TOJET, 11(4):283–294, 2012.

[35] Katherine Chretien, Ellen Goldman, and Charles Faselis. The reflective writing class blog: using technology to promote reflection and professional development. Journal of general internal medicine, 23(12):2066–2070, 2008.

[36] Musa Citak, Afshin Calafi, Daniel Kendoff, Thomas Kupka, Carl Haasper, Marianne Behrends, Christian Krettek, Herbert K Matthies, and Tobias Hufner. An internet based learning tool in orthopaedic surgery: preliminary experiences and results. Technology and Health Care, 17(2):141–148, 2009.

[37] Laura Cobus. Using blogs and wikis in a graduate public health course. Medical reference services quarterly, 28(1):22–32, 2009.

[38] Rosa M Coco, M Rosa Sanabria, and Itziar Fernandez. E-learning strategies to improve general practitioners' knowledge of age-related macular degeneration. Medical education, 46(5):517–518, 2012.

[39] Anthony M Codd and Bipasha Choudhury. Virtual reality anatomy: Is it comparable with traditional methods in the teaching of human forearm musculoskeletal anatomy? Anatomical sciences education, 4(3):119–125, 2011.

[40] Zoe Cohen and J John Cohen. Inflammablog: Peer-to-peer online learning in immunology. Immunologic research, 55(1-3):71–74, 2013.

[41] Mark Corrigan, Seamus McHugh, Athar Sheikh, Elaine Lehane, Conor Shields, Paul Redmond, Michael Kerin, and Arnold Hill. Surgent university the establishment and evaluation of a national online clinical teaching repository for surgical trainees and students. Surgical innovation, 19(2):200–204, 2012.

[42] Mark Corrigan, Michelle Reardon, Connor Shields, and Henry Redmond. “surgent”—student e-learning for reality: The application of interactive visual images to problem-based learning in undergraduate surgery. Journal of surgical education, 65(2):120–125, 2008.

[43] O Courteille, R Bergin, O Courteille, R Bergin, D Stockeld, S Ponzer, and U Fors. The use of a virtual patient case in an osce-based exam-a pilot study. Medical Teacher, 30(3):e66–e76, 2008.

[44] Johan Creutzfeldt, Leif Hedman, and Li Fellander-Tsai. Effects of pre-training using serious game technology on cpr performance–an exploratory quasi-experimental transfer study. Scandinavian journal of trauma, resuscitation and emergency medicine, 20(1):1–9, 2012.

[45] LA Critchley, SM Kumta, J Ware, and JW Wong. Web-based formative assessment case studies: role in a final year medicine two-week anaesthesia course. Anaesth Intensive Care, 37(4):637–645, 2009.

[46] Brad Dalton, Keith McNeil, Anne Keogh, Trevor Williams, S Proudman, E Gabbay, Eugene Kotlyar, Robert Weintraub, F Kermeen, David Celermajer, et al. Design and delivery of an e-learning curriculum for physicians involved in the management of pulmonary hypertension. International journal of clinical practice, 66(11):1117–1124, 2012.

[47] Laura A Daunt, Patience I Umeonusulu, John RF Gladman, Adrian G Blundell, Simon P Conroy, and Adam L Gordon. Undergraduate teaching in geriatric medicine using computer-aided learning improves student performance in examinations. Age and ageing, 42(4):541–544, 2013.

[48] Mogamat Razeen Davids, Usuf ME Chikte, and Mitchell L Halperin. Development and evaluation of a multimedia e-learning resource for electrolyte and acid-base disorders. Advances in Physiology Education, 35(3):295–306, 2011.

[49] Lindsay K Davidson. A 3-year experience implementing blended tbl: Active instructional methods can shift student attitudes to learning. Medical teacher, 33(9):750–753, 2011.

[50] Bethany S Davies, Jethin Rafique, Tim R Vincent, Jil Fairclough, Mark H Packer, Richard Vincent, and Inam Haq. Mobile medical education (momed)-how mobile information resources contribute to learning for undergraduate clinical students-a mixed methods study. BMC medical education, 12(1):1, 2012.

[51] James Davis, Evi Chryssafidou, Javier Zamora, David Davies, Khalid Khan, and Arri Coomarasamy. Computer-based teaching is as good as face to face lecture-based teaching of evidence based medicine: a randomised controlled trial. BMC medical education, 7(1):23, 2007.

[52] James S Davis, George D Garcia, Mary M Wyckoff, Salman Alsafran, Jill M Graygo, Kelly F Withum, and Carl I Schulman. Use of mobile learning module improves skills in chest tube insertion. Journal of Surgical Research, 177(1):21–26, 2012.

[53] Rosilaine de Fátima Wardenski, Marina Bazzo de Espíndola, Miriam Struchiner, and Taís Rabetti Giannella. Blended learning in biochemistry education: Analysis of medical students' perceptions. Biochemistry and Molecular Biology Education, 40(4):222–228, 2012.

[54] Bas A De Leng, Diana HJM Dolmans, Arno MM Muijtjens, and Cees PM Van Der Vleuten. Student perceptions of a virtual learning environment for a problem-based learning undergraduate medical curriculum. Medical education, 40(6):568–575, 2006.

[55] Bas A De Leng, Diana HJM Dolmans, Margje WJ Van de Wiel, AMM Muijtjens, and Cees PM Van Der Vleuten. How video cases should be used as authentic stimuli in problem-based medical education. Medical education, 41(2):181–188, 2007.

[56] Sandrine de Ribaupierre and Timothy D Wilson. Construction of a 3-d anatomical model for teaching temporal lobectomy. Computers in biology and medicine, 42(6):692–696, 2012.

[57] Tejas Desai, Darina Stankeyeva, Arlene Chapman, and James Bailey. Nephrology fellows show consistent use of, and improved knowledge from, a nephrologist-programmed teaching instrument. Journal of nephrology, 24(3):345, 2011.

[58] Pieter Devolder, Bram Pynoo, Tony Voet, Luc Adang, Jan Vercruysse, and Philippe Duyck. Optimizing physicians' instruction of pacs through e-learning: cognitive load theory applied. Journal of digital imaging, 22(1):25–33, 2009.

[59] Stefanie Diessl, Frederik A Verburg, Alexander Hoernlein, Martin Schumann, Markus Luster, and Christoph Reiners. Evaluation of an internet-based e-learning module to introduce nuclear medicine to medical students: a feasibility study. Nuclear medicine communications, 31(12):1063–1067, 2010.

[60] Aditya Dikshit, Dawei Wu, Chunyan Wu, and Weizhao Zhao. An online interactive simulation system for medical imaging education. Computerized Medical Imaging and Graphics, 29(6):395–404, 2005.

[61] Gustavo Duque, Oddom Demontiero, Sarah Whereat, Piumali Gunawardene, Oliver Leung, Peter Webster, Luis Sardinha, Derek Boersma, and Anita Sharma. Evaluation of a blended learning model in geriatric medicine: A successful learning experience for medical students. Australasian journal on ageing, 32(2):103–109, 2013.

[62] Olayemi Durosaro, Nirusha Lachman, and Wojciech Pawlina. Use of knowledge-sharing web-based portal in gross and microscopic anatomy. Annals Academy of Medicine Singapore, 37(12):998, 2008.

[63] Sydney Morss Dy, Mark Hughes, Carlos Weiss, and Stephen Sisson. Evaluation of a web-based palliative care pain management module for housestaff. Journal of pain and symptom management, 36(6):596–603, 2008.

[64] Rachel Ellaway, David Dewhurst, and Allan Cumming. Managing and supporting medical education with a virtual learning environment: the edinburgh electronic medical curriculum. Medical Teacher, 25(4):372–380, 2003.

[65] H Farrimond, TL Dornan, A Cockcroft, and LE Rhodes. Development and evaluation of an e-learning package for teaching skin examination. action research. British Journal of Dermatology, 155(3):592–599, 2006.

[66] Mark Feist, Mary Ciccarelli, Brian A McFerron, and Jean P Molleston. Methods and effects of a case-based pediatric gastroenterology online curriculum. Journal of pediatric gastroenterology and nutrition, 56(2):161–165, 2013.

[67] RG Finch, FB Blasi, TJM Verheij, H Goossens, S Coenen, K Loens, G Rohde, H Saenz, and M Akova. Grace and the development of an education and training curriculum. Clinical Microbiology and Infection, 18(9):E308–E313, 2012.

[68] Michael Fleming, Dale Olsen, Hilary Stathes, Laura Boteler, Paul Grossberg, Judie Pfeifer, Stephanie Schiro, Jane Banning, and Susan Skochelak. Virtual reality skills training for health care professionals in alcohol screening and brief intervention. The Journal of the American Board of Family Medicine, 22(4):387–398, 2009.

[69] Farshad Foroudi, Daniel Pham, Mathias Bressel, David Tongs, Aldo Rolfo, Colin Styles, Suki Gill, and Tomas Kron. The utility of e-learning to support training for a multicentre bladder online adaptive radiotherapy trial (trog 10.01-bolart). Radiotherapy and Oncology, 109(1):165–169, 2013.

[70] R Friedl, H Hoppler, K Ecard, W Scholz, A Hannekum, and S Stracke. Development and prospective evaluation of a multimedia teaching course on aortic valve replacement. The Thoracic and cardiovascular surgeon, 54(1):1–9, 2006.

[71] Reinhard Friedl, Helmut Hoppler, Karl Ecard, Wilfried Scholz, Andreas Hannekum, Wolfgang Ochsner, and Sylvia Stracke. Multimedia-driven teaching significantly improves students' performance when compared with a print medium. The Annals of thoracic surgery, 81(5):1760–1766, 2006.

[72] Katrin Funke, Esther Bonrath, Wolf Arif Mardin, Jan Carl Becker, Joerg Haier, Norbert Senninger, Thorsten Vowinkel, Jens Peter Hoelzen, and Soeren Torge Mees. Blended learning in surgery using the inmedea simulator. Langenbeck's Archives of Surgery, 398(2):335–340, 2013.

[73] Bernard Mark Garrett and Cathryn Jackson. A mobile clinical e-portfolio for nursing and medical students, using wireless personal digital assistants (pdas). Nurse Education Today, 26(8):647–654, 2006.

[74] Paul George, Luba Dumenco, Richard Dollase, Julie Scott Taylor, Hedy S Wald, and Shmuel P Reis. Introducing technology into medical education: Two pilot studies. Patient education and counseling, 93(3):522–524, 2013.

[75] Martin Geueke and Jurgen Stausberg. A meta-data-based learning resource server for medicine. Computer methods and programs in biomedicine, 72(3):197–208, 2003.

[76] Peter Gill, Lauren Kitney, Daniel Kozan, and Melanie Lewis. Online learning in paediatrics: a student-led web-based learning modality. The clinical teacher, 7(1):53–57, 2010.

[77] Fernanda Ginani, Rodrigo Gadelha Vasconcelos, and Carlos Augusto Galvão Barboza. Use of clinical cases in a virtual learning environment as an approach to teaching human embryology. Int. J. Morphol, 30(4):1395–1398, 2012.

[78] Serafinn Sánchez Gómez, Elisa María Cabot Ostos, Juan Manuel Maza Solano, and Tomás Francisco Herrero Salado. An electronic portfolio for quantitative assessment of surgical skills in undergraduate medical education. BMC medical education, 13(1):65, 2013.

[79] Morris Gordon, Madawa Chandratilake, and Paul Baker. Improved junior paediatric prescribing skills after a short e-learning intervention: a randomised controlled trial. Archives of disease in childhood, 96(12):1191–1194, 2011.

[80] Gerard J Gormley, Kieran Mcglade, Clare Thomson, Maria Mcgill, and Julia Sun. A virtual surgery in general practice: Evaluation of a novel undergraduate virtual patient learning package. Medical teacher, 33(10):e522–e527, 2011.

[81] Martin Gotthardt, Maria J Siegert, Anja Schlieck, Stefan Schneider, Alfred Kohnert, Markus W Grob, Christine Schafer, Richard Wagner, Stefan Hormann, Thomas M Behr, et al. How to successfully implement e-learning for both students and teachers. Academic radiology, 13(3):379–390, 2006.

[82] Douglas J Gould, Mark A Terrell, and Jo Fleming. A usability study of users' perceptions toward a multimedia computer-assisted learning tool for neuroanatomy. Anatomical sciences education, 1(4):175–183, 2008.

[83] Matthaeus C Grasl, Peter Pokieser, Andreas Gleiss, Juergen Brandstaetter, Thorsten Sigmund, Boban M Erovic, and Martin R Fischer. A new blended learning concept for medical students in otolaryngology. Archives of Otolaryngology–Head & Neck Surgery, 138(4):358–366, 2012.

[84] Kathleen Gray and Jacinta Tobin. Introducing an online community into a clinical education setting: a pilot study of student and staff engagement and outcomes using blended learning. BMC medical education, 10(1):6, 2010.

[85] Michael J Green and Benjamin H Levi. Teaching advance care planning to medical students with a computer-based decision aid. Journal of Cancer Education, 26(1):82–91, 2011.

[86] Karen Gresty, Heather Skirton, and Andrew Evenden. Addressing the issue of e-learning and online genetics for health professionals. Nursing & health sciences, 9(1):14–22, 2007.

[87] Rolf HH Groenwold and Mirjam J Knol. Learning styles and preferences for live and distance education: an example of a specialisation course in epidemiology. BMC medical education, 13(1):93, 2013.

[88] Denise Guliato, Ricardo S Bôaventura, Marcelo A Maia, Rangaraj M Rangayyan, Mariângela S Simedo, and Túlio AA Macedo. Indiam—an e-learning system for the interpretation of mammograms. Journal of digital imaging, 22(4):405–420, 2009.

[89] William T Gunning and Uno GH Fors. Virtual patients for assessment of medical student ability to integrate clinical and laboratory data to develop differential diagnoses: comparison of results of exams with/without time constraints. Medical teacher, 34(4):e222–e228, 2012.

[90] David E Gyorki, Tim Shaw, James Nicholson, Caroline Baker, Meron Pitcher, Anita Skandarajah, Eva Segelov, and G Bruce Mann. Improving the impact of didactic resident training with online spaced education. ANZ journal of surgery, 83(6):477–480, 2013.

[91] Julie Hadley, Regina Kulier, Javier Zamora, Sjors FPJ Coppus, Susanne Weinbrenner, Berrit Meyerrose, Tamas Decsi, Andrea R Horvath, Eva Nagy, Jose I Emparanza, et al. Effectiveness of an e-learning course in evidence-based medicine for foundation (internship) training. Journal of the Royal Society of Medicine, 103(7):288–294, 2010.

[92] Josef Hanus, Tomas Nosek, Jiri Zahora, Ales Bezrouk, and Vladimir Masin. On-line integration of computer controlled diagnostic devices and medical information systems in undergraduate medical physics education for physicians. Physica Medica, 29(1):83–90, 2013.

[93] RM Harden, IH Gessner, M Gunn, SB Issenberg, SD Pringle, and A Stewart. Creating an e-learning module from learning objects using a commentary or’personal learning assistant’. Medical teacher, 33(4):286–290, 2011.

[94] Andrea Hards, Sharon Davies, Aliya Salman, Magda Erik-Soussi, and Mrinalini Balki. Management of simulated maternal cardiac arrest by residents: didactic teaching versus electronic learning. Canadian Journal of Anesthesia/Journal canadien d’anesthésie, 59(9):852–860, 2012.

[95] Andrew B Hardy, Llinos Jones, and Jack Kastelik. Development of a web-based training programme for respiratory physicians in yorkshire. Clinical medicine, 10(4):344–348, 2010.

[96] Susie T Harris and Xiaoming Zeng. Using wiki in an online record documentation systems course. Perspectives in health information management/AHIMA, American Health Information Management Association, 5, 2008.

[97] John M Harris Jr and Huaping Sun. A randomized trial of two e-learning strategies for teaching substance abuse management skills to physicians. Academic medicine: journal of the Association of American Medical Colleges, 88(9):1357, 2013.

[98] Mohammed Ahmed Hassanien, Abdulmoneam Al-Hayani, Rasha Abu-Kamer, and Adnan Almazrooa. A six step approach for developing computer based assessment in medical education. Medical teacher, 35(s1):S15–S19, 2013.

[99] Kamila Hawthorne, Hayley Prout, Paul Kinnersley, and Helen Houston. Evaluation of different delivery modes of an interactive e-learning programme for teaching cultural diversity. Patient education and counseling, 74(1):5–11, 2009.

[100] Heather L Heiman, Toshiko Uchida, Craig Adams, John Butter, Elaine Cohen, Stephen D Persell, Paul Pribaz, William C McGaghie, and Gary J Martin. E-learning and deliberate practice for oral case presentation skills: A randomized trial. Medical teacher, 34(12):e820–e826, 2012.

[101] Ann Helms, Kathryn Denson, Diane Brown, and Deborah Simpson. One specialty at a time: Achieving competency in geriatrics through an e-learning neurology clerkship module. Academic Medicine, 84(10):S67–S69, 2009.

[102] William Hendee, Jennifer L Bosma, Linda B Bresolin, Leonard Berlin, R Nick Bryan, and Richard B Gunderman. Web modules on professionalism and ethics. Journal of the American College of Radiology, 9(3):170–173, 2012.

[103] Craig K Henkel. Creating interactive learning objects with powerpoint: primer for lecture on the autonomic nervous system. Medical teacher, 32(8):e355–e359, 2010.

[104] Pouria Heydarpour, Nima Hafezi-Nejad, Ali Khodabakhsh, Mohsen Khosravi, Shayan Khoshkish, Majid Sadeghian, Bijan Samavat, Ali Faturechi, Parvin Pasalar, and Ahmad Reza Dehpour. Medical podcasting in iran; pilot, implementation and attitude evaluation. Acta Medica Iranica, 51(1):59–61, 2013.

[105] Denis Hoa, Antoine Micheau, and Gerald Gahide. Creating an interactive web-based e-learning course: A practical introduction for radiologists 1. Radiographics, 26(6):e25–e25, 2006.

[106] Andreas Holzinger, Michael D Kickmeier-Rust, Sigi Wassertheurer, and Michael Hessinger. Learning performance with interactive simulations in medical education: Lessons learned from results of learning complex physiological models with the haemodynamics simulator. Computers & Education, 52(2):292–301, 2009.

[107] M Horstmann, M Renninger, J Hennenlotter, CC Horstmann, and A Stenzl. Blended e-learning in a web-based virtual hospital: a useful tool for undergraduate education in urology. Education for health (Abingdon, England), 22(2):269–269, 2009.

[108] Marcus Horstmann, Carolin Horstmann, Markus Renninger, et al. Case creation and e-learning in a web-based virtual department of urology using the inmedea simulator. Nephro-Urology Monthly, 4(01):356–360, 2011.

[109] Chia-Hung Hsiao, Tien-Cheng Hsu, Jing Ning Chang, Stephen JH Yang, Shuenn-Tsong Young, and Woei Chyn Chu. Developing a medical image content repository for e-learning. Journal of digital imaging, 19(3):207–215, 2006.

[110] Amanda Hu, Tim Wilson, Hanif Ladak, Peter Haase, Philip Doyle, and Kevin Fung. Evaluation of a three-dimensional educational computer model of the larynx: voicing a new direction. Journal of otolaryngology-head & neck surgery= Le Journal d'oto-rhino-laryngologie et de chirurgie cervico-faciale, 39(3):315–322, 2010.

[111] Soren Huwendiek, Cecilia Duncker, Friedrich Reichert, Bas A De Leng, Diana Dolmans, Cees PM van der Vleuten, Martin Haag, Georg Friedrich Hoffmann, and Burkhard Tonshoff. Learner preferences regarding integrating, sequencing and aligning virtual patients with other activities in the undergraduate medical curriculum: A focus group study. Medical teacher, 35(11):920–929, 2013.

[112] Jane Ichord. Virtual information for patient care—vip care: An electronic collection to promote learning among third-year medical students. Medical reference services quarterly, 28(2):123–132, 2009.

[113] Josie Naomi Iyeyasu, Renato Marcos Endrizzi Sabbatini, Keila Monteiro de Carvalho, et al. The development and evaluation of a distance learning system in ophthalmology. Revista Brasileira de Educação Médica, 37(1):96–102, 2013.

[114] Akram Abood Jaffar. Youtube: An emerging tool in anatomy education. Anatomical sciences education, 5(3):158–164, 2012.

[115] A Kalinowska-Przybyłko, J Kowalczyk-Nowakowska, B Baranowska, and E Dmoch-Gajzlerska. On-line seminars in the education of warsaw medical university students. Progress in Health Sciences, 2(1), 2012.

[116] F Kaliyadan, J Manoj, AD Dharmaratnam, and G Sreekanth. Self-learning digital modules in dermatology: a pilot study. Journal of the European Academy of Dermatology and Venereology, 24(6):655–660, 2010.

[117] Thileeban Kandasamy and Kevin Fung. Interactive internet-based cases for undergraduate otolaryngology education. Otolaryngology–Head and Neck Surgery, 140(3):398–402, 2009.

[118] B Price Kerfoot. Interactive spaced education versus web based modules for teaching urology to medical students: a randomized controlled trial. The Journal of Urology, 179(6):2351–2357, 2008.

[119] B Price Kerfoot. Learning benefits of on-line spaced education persist for 2 years. The Journal of urology, 181(6):2671–2673, 2009.

[120] B Price Kerfoot and Harley Baker. An online spaced-education game for global continuing medical education: a randomized trial. Annals of surgery, 256(1):33–38, 2012.

[121] B Price Kerfoot, Harley E Baker, Michael O Koch, Donna Connelly, David B Joseph, and Michael L Ritchey. Randomized, controlled trial of spaced education to urology residents in the united states and canada. The Journal of Urology, 177(4):1481–1487, 2007.

[122] B Price Kerfoot and Erica Brotschi. Online spaced education to teach urology to medical students: a multi-institutional randomized trial. The American Journal of Surgery, 197(1):89–95, 2009.

[123] B Price Kerfoot, William C DeWolf, Barbara A Masser, Paul A Church, and Daniel D Federman. Spaced education improves the retention of clinical knowledge by medical students: a randomised controlled trial. Medical education, 41(1):23–31, 2007.

[124] B Price Kerfoot, Yineng Fu, Harley Baker, Donna Connelly, Michael L Ritchey, and Elizabeth M Genega. Online spaced education generates transfer and improves long-term retention of diagnostic skills: a randomized controlled trial. Journal of the American College of Surgeons, 211(3):331–337, 2010.

[125] SEO Khogali, David A Davies, PT Donnan, Alastair Gray, Ronald M Harden, J McDonald, MJ Pippard, Steven D Pringle, and N Yu. Integration of e-learning resources into a medical school curriculum. Medical teacher, 33(4):311–318, 2011.

[126] Zaid Khot, Kaitlyn Quinlan, Geoffrey R Norman, and Bruce Wainman. The relative effectiveness of computer-based and traditional resources for education in anatomy. Anatomical sciences education, 6(4):211–215, 2013.

[127] Kyong-Jee Kim, Joungho Han, IeB Park, and Changwon Kee. Evaluating effectiveness and usability of an e-learning portal: the e-mededu site. Medical teacher, 32(8):702–703, 2009.

[128] Roger Kneebone, Fernando Bello, Debra Nestel, Neville Mooney, Andrew Codling, Faranak Yadollahi, Tanya Tierney, David Wilcockson, and Ara Darzi. Learner-centred feedback using remote assessment of clinical procedures. Medical teacher, 30(8):795–801, 2008.

[129] Jiri Kofranek, Stanislav Matousek, Jan Rusz, Petr Stodulka, Pavol Privitzer, Marek Matejak, and Martin Tribula. The atlas of physiology and pathophysiology: Web-based multimedia enabled interactive simulations. Computer methods and programs in biomedicine, 104(2):143–153, 2011.

[130] S Kolb, J Reichert, I Hege, G Praml, MC Bellido, B Martinez-Jaretta, M Fischer, D Nowak, K Radon, et al. European dissemination of a web- and case-based learning system for occupational medicine: Networm europe. International archives of occupational and environmental health, 80(6):553–557, 2007.

[131] Martin Komenda, Daniel Schwarz, Jitka Feberova, Stanislav Štipek, Vladimir Mihal, and Ladislav Dušek. Medical faculties educational network: Multidimensional quality assessment. Computer methods and programs in biomedicine, 108(3):900–909, 2012.

[132] Andrzej A Kononowicz, Paweł Krawczyk, Grzegorz Cebula, Marta Dembkowska, Edyta Drab, Bartosz Fraczek, Aleksandra J Stachoń, and Janusz Andres. Effects of introducing a voluntary virtual patient module to a basic life support with an automated external defibrillator course: a randomised trial. BMC medical education, 12(1):41, 2012.

[133] Doris Kraemer, Stanislaus Reimer, Alexander Hornlein, Christian Betz, Frank Puppe, and Christian Kneitz. Evaluation of a novel case-based training program (d3web. train) in hematology. Annals of hematology, 84(12):823–829, 2005.

[134] Klaus-Dietrich Kroncke. Computer-based learning versus practical course in pre-clinical education: Acceptance and knowledge retention. Medical teacher, 32(5):408–413, 2010.

[135] Sunčana Kukolja Taradi, Zoran ogaš, Marina Dabić, and Ines Drenjančević Perić. Scaling-up undergraduate medical education: enabling virtual mobility by online elective courses. Croatian medical journal, 49(3):344–351, 2008.

[136] Regina Kulier, Julie Hadley, Susanne Weinbrenner, Berrit Meyerrose, Tamas Decsi, Andrea R Horvath, Eva Nagy, Jose I Emparanza, Sjors FPJ Coppus, Theodoros N Arvanitis, et al. Harmonising evidence-based medicine teaching: a study of the outcomes of e-learning in five european countries. BMC Medical Education, 8(1):27, 2008.

[137] Avinash B Kumar, J Steven Hata, Emine O Bayman, and Sundar Krishnan. Implementing a hybrid web-based curriculum for an elective medical student clerkship in a busy surgical intensive care unit (icu): Effect on test and satisfaction scores. Journal of surgical education, 70(1):109–116, 2013.

[138] Ronny Lehmann, Hans M Bosse, and Soren Huwendiek. Blended learning using virtual patients and skills laboratory training. Medical education, 44(5):521–522, 2010.

[139] Ronny Lehmann, Hans Martin Bosse, Anke Simon, Christoph Nikendei, and Soren Huwendiek. An innovative blended learning approach using virtual patients as preparation for skills laboratory training: perceptions of students and tutors. BMC medical education, 13(1):23, 2013.

[140] Sum Leong, Patrick Mc Laughlin, Owen J O'Connor, Siun O'Flynn, and Michael M Maher. An assessment of the feasibility and effectiveness of an e-learning module in delivering a curriculum in radiation protection to undergraduate medical students. Journal of the American College of Radiology, 9(3):203–209, 2012.

[141] Joseph YC Leung, Lester AH Critchley, Alex LK Yung, and Shekhar M Kumta. Introduction of virtual patients onto a final year anesthesia course: Hong kong experience. Advances in Medical Education and Practice, 2:71, 2011.

[142] Linda O Lewin, Mamta Singh, Betzi L Bateman, and Pamela B Glover. Improving education in primary care: development of an online curriculum using the blended learning model. BMC medical education, 9(1):33, 2009.

[143] Erle CH Lim, Raymond Seet, and Benjamin KC Ong. A core skills simulation module. Medical education, 40(11):1135–1136, 2006.

[144] Chao-Cheng Lin, Yu-Chuan Li, Ya-Mei Bai, Jen-Yeu Chen, Chien-Yeh Hsu, Chih-Hung Wang, Hung-Wen Chiu, and Hsu-Tien Wan. The evaluation of game-based e-learning for medical education: a preliminary survey. In AMIA Annual Symposium Proceedings, volume 2005, page 1032. American Medical Informatics Association, 2005.

[145] Thomas M Link and Richard Marz. Computer literacy and attitudes towards e-learning among first year medical students. BMC medical education, 6(1):34, 2006.

[146] Laura Llambi, Elba Esteves, Elisa Martinez, Thais Forster, Sofia Garcia, Natalia Miranda, Antonio Lopez Arredondo, and Alvaro Margolis. Teaching tobacco cessation skills to uruguayan physicians using information and communication technologies. Journal of Continuing Education in the Health Professions, 31(1):43–48, 2011.

[147] Craig Locatis, Eta S Berner, Glenn Hammack, Steve Smith, Richard Maisiak, and Michael Ackerman. An exploratory study of co-location as a factor in synchronous, collaborative medical informatics distance education. BMC research notes, 3(1):30, 2010.

[148] Jenny Lorimer and Alan Hilliard. Incorporating learning technologies into undergraduate radiography education. Radiography, 15(3):214–219, 2009.

[149] Jianfeng Lu, Li Li, and Goh Poh Sun. A multimodal virtual anatomy e-learning tool for medical education. In Entertainment for Education. Digital Techniques and Systems, pages 278–287. Springer, 2010.

[150] Esther M Maier, Inga Hege, Ania C Muntau, Johanna Huber, and Martin R Fischer. What are effects of a spaced activation of virtual patients in a pediatric course? BMC medical education, 13(1):45, 2013.

[151] Samuel Mark Keim, David Howse, Paul Bracke, and Kathryn Mendoza. Promoting evidence based medicine in preclinical medical students via a federated literature search tool. Medical teacher, 30(9-10):880–884, 2008.

[152] Andrew Marks, Max Maizels, Jennie Mickelson, Elizabeth Yerkes, CD Anthony Herndon, Jerry Lane, Tamar Ben-Ami, Evelyn Maizels, Rachel Stork Stoltz, Scott Dixon, et al. Effectiveness of the computer enhanced visual learning method in teaching the society for fetal urology hydronephrosis grading system for urology trainees. Journal of pediatric urology, 7(2):113–117, 2011.

[153] Nina L Marshall, Muirne Spooner, P Leo Galvin, Joanna P Ti, N Gerald McElvaney, and Michael J Lee. Informatics in radiology: evaluation of an e-learning platform for teaching medical students competency in ordering radiologic examinations. Radiographics, 31(5):1463–1474, 2011.

[154] Italo Masiello, Robert Ramberg, and Kirsti Lonka. Attitudes to the application of a web-based learning system in a microbiology course. Computers & Education, 45(2):171–185, 2005.

[155] Kimberly A Matzie, B Price Kerfoot, Janet P Hafler, and Elizabeth M Breen. Spaced education improves the feedback that surgical residents give to medical students: a randomized trial. The American Journal of Surgery, 197(2):252–257, 2009.

[156] Patrick E McCleskey. Clinic teaching made easy: A prospective study of the american academy of dermatology core curriculum in primary care learners. Journal of the American Academy of Dermatology, 69(2):273–279, 2013.

[157] Seamus Mark McHugh, Mark Corrigan, Borislav Dimitrov, Seamus Cowman, Sean Tierney, Hilary Humphreys, and Arnold Hill. A targeted e-learning program for surgical trainees to enhance patient safety in preventing surgical infection. Journal of Continuing Education in the Health Professions, 30(4):257–259, 2010.

[158] Danielle McKenna, Caroline Wilkinson, and Jean Ker. Diagnostic recognition of facial changes associated with chronic conditions: Use of an e-learning tool to enhance medical student education. Journal of visual communication in medicine, 33(2):55–62, 2010.

[159] Thomas G Mcleod, Dean A McNaughton, Gregory J Hanson, and Stephen S Cha. Educational effectiveness of a personal digital assistant-based geriatric assessment tool. Medical teacher, 31(5):409–414, 2009.

[160] David C McMillan. Web-based course in neuroimmune pharmacology. Journal of Neuroimmune Pharmacology, 6(1):76–79, 2011.

[161] Stefan Moeller, Klaus Spitzer, and Cord Spreckelsen. How to configure blended problem based learning-results of a randomized trial. Medical teacher, 32(8):e328–e346, 2010.

[162] Pablo Moreno-Ger, Javier Torrente, Julián Bustamante, Carmen Fernández-Galaz, Baltasar Fernández-Manjón, and María Dolores Comas-Rengifo. Application of a low-cost web-based simulation to improve students' practical skills in medical education. International Journal of Medical Informatics, 79(6):459–467, 2010.

[163] Yuri Morgulis, Rakesh K Kumar, Robert Lindeman, and Gary M Velan. Impact on learning of an e-learning module on leukaemia: a randomised controlled trial. BMC medical education, 12(1):36, 2012.

[164] JP Naidr, T Adla, A Janda, J Feberova, P Kasal, and M Hladikova. Long-term retention of knowledge after a distance course in medical informatics at charles university prague. Teaching and learning in medicine, 16(3):255–259, 2004.

[165] Neeraj Narula, Liban Ahmed, and Jill Rudkowski. An evaluation of the'5 minute medicine'video podcast series compared to conventional medical resources for the internal medicine clerkship. Medical teacher, 34(11):e751–e755, 2012.

[166] Alexander Nast, Gregor Schafer-Hesterberg, Hendrik Zielke, Wolfram Sterry, and Berthold Rzany. Online lectures for students in dermatology: A replacement for traditional teaching or a valuable addition? Journal of the European Academy of Dermatology and Venereology, 23(9):1039–1043, 2009.

[167] Gary L Nieder and Nicole J Borges. An eight-year study of online lecture use in a medical gross anatomy and embryology course. Anatomical sciences education, 5(6):311–320, 2012.

[168] Mikael Nilsson, Gunilla Bolinder, Claes Held, Bo-Lennart Johansson, Uno Fors, and Jan Ostergren. Evaluation of a web-based ecg-interpretation programme for undergraduate medical students. BMC medical education, 8(1):25, 2008.

[169] Fenton M O'Leary. Paediatric resuscitation training: Is e-learning the answer? a before and after pilot study. Journal of paediatrics and child health, 48(6):529–533, 2012.

[170] Fenton M O'Leary and Philip Janson. Can e-learning improve medical students' knowledge and competence in paediatric cardiopulmonary resuscitation? a prospective before and after study. Emergency Medicine Australasia, 22(4):324–329, 2010.

[171] GS Oliveira, R Glassenberg, R Chang, P Fitzgerald, and RJ McCarthy. Virtual airway simulation to improve dexterity among novices performing fibreoptic intubation. Anaesthesia, 68(10):1053–1058, 2013.

[172] Kevin O'Regan, Paul Marsden, Gerardine Sayers, Mary Morrissey, Heather Hegarty, Michael Allen, Owen J O'Connor, Dermot Malone, and Michael M Maher. Videoconferencing of a national program for residents on evidence-based practice: early performance evaluation. Journal of the American College of Radiology, 7(2):138–145, 2010.

[173] E Medélez Ortega, Y Lessard, A Burgun, and P Le Beux. Virtu@ i consult@ tion: an interactive and multimedia environment for remote clinical reasoning learning in cardiology. In Computers in Cardiology, 2005, pages 829–832. IEEE, 2005.

[174] Elizabeth Medélez Ortega, Anita Burgun, Franck Le Duff, and Pierre Le Beux. Collaborative environment for clinical reasoning and distance learning sessions. International journal of medical informatics, 70(2):345–351, 2003.

[175] Eric Orton and Paul Mulhausen. E-learning virtual patients for geriatric education. Gerontology & geriatrics education, 28(3):73–88, 2008.

[176] E O'Neill, NT Stevens, E Clarke, P Cox, B O'Malley, and H Humphreys. Use of e-learning to enhance medical students' understanding and knowledge of healthcare-associated infection prevention and control. Journal of Hospital Infection, 79(4):368–370, 2011.

[177] N Pallikarakis. Development and evaluation of an odl course on medical image processing. Medical engineering & physics, 27(7):549–554, 2005.

[178] Thomas NB Pascual, Maurizio Dondi, Diana Paez, Ravi Kashyap, and Rodolfo Nunez-Miller. Iaea programs in empowering the nuclear medicine profession through online educational resources. In Seminars in nuclear medicine, volume 43, pages 161–166. Elsevier, 2013.

[179] P Pavese, M Coulouma, E Sellier, J-P Stahl, C Wintenberger, and P François. Cd-rom continuous medical education model for the management of urinary tract infections in family practice. Médecine et maladies infectieuses, 42(7):321–326, 2012.

[180] Oliver Peacock, Edward Watts, David Foreman, Jonathan N Lund, and Gillian M Tierney. Evaluation of teaching methods for students on hernias: an observational study. ANZ journal of surgery, 83(1-2):11–14, 2013.

[181] José A Pereira, Eulogio Pleguezuelos, Alex Merí, Antoni Molina-Ros, M Carmen Molina-Tomás, and Carlos Masdeu. Effectiveness of using blended learning strategies for teaching and learning human anatomy. Medical education, 41(2):189–195, 2007.

[182] Gavin D Perkins, James N Fullerton, Nicole Davis-Gomez, Robin P Davies, Catherine Baldock, Harry Stevens, Ian Bullock, and Andrew S Lockey. The effect of pre-course e-learning prior to advanced life support training: a randomised controlled trial. Resuscitation, 81(7):877–881, 2010.

[183] Gavin D Perkins, Peter K Kimani, Ian Bullock, Tom Clutton-Brock, Robin P Davies, Mike Gale, Jenny Lam, Andrew Lockey, Nigel Stallard, et al. Improving the efficiency of advanced life support training: a randomized, controlled trial. Annals of internal medicine, 157(1):19–28, 2012.

[184] Livia Puljak and Damir Sapunar. Web-based elective courses for medical students: An example in pain. Pain Medicine, 12(6):854–863, 2011.

[185] Katja Radon, Stefanie Kolb, Jorg Reichert, Thomas Baumeister, Reinhard Fuchs, Inga Hege, Georg Praml, Martin Fischer, and Dennis Nowak. Case-based e-learning in occupational medicine- the networm project in germany. Annals of Agricultural and Environmental Medicine, 13(1):93–98, 2006.

[186] Rajkumar Rajasekaran and Nallani Chackravatula Sriman Narayana Iyengar. Peer-to-peer jxta architecture for continuing mobile medical education incorporated in rural public health centers. Osong Public Health and Research Perspectives, 4(2):99–106, 2013.

[187] T Raupach, C Munscher, T Pukrop, S Anders, and S Harendza. Significant increase in factual knowledge with web-assisted problem-based learning as part of an undergraduate cardio-respiratory curriculum. Advances in health sciences education, 15(3):349–356, 2010.

[188] Peter Rich and Richard Guy. A “do-it-yourself” interactive bone structure module: Development and evaluation of an online teaching resource. Anatomical sciences education, 6(2):107–113, 2013.

[189] Paul F Ridgway, Athar Sheikh, Karl J Sweeney, Denis Evoy, Enda McDermott, Patrick Felle, Arnold D Hill, and Niall J O’Higgins. Surgical e-learning: validation of multimedia web-based lectures. Medical education, 41(2):168–172, 2007.

[190] Gillian Robb, Susan Wells, and Felicity Goodyear-Smith. Values add value: An online tool enhances postgraduate evidence-based practice learning. Medical teacher, 34(11):e743–e750, 2012.

[191] Chris Roberts, Mary Lawson, David Newble, and Ashley Self. Managing the learning environment in undergraduate medical education: the sheffield approach. Medical teacher, 25(3):282–286, 2003.

[192] Cristóbal Romero, Sebastián Ventura, Eva L Gibaja, Cesar Hervás, and Francisco Romero. Web-based adaptive training simulator system for cardiac life support. Artificial Intelligence in Medicine, 38(1):67–78, 2006.

[193] Pablo Ruisoto, Juan Antonio Juanes, Israel Contador, Paula Mayoral, and Alberto Prats-Galino. Experimental evidence for improved neuroimaging interpretation using three-dimensional graphic models. Anatomical sciences education, 5(3):132–137, 2012.

[194] Jorge G Ruiz, Michael Smith, Osvaldo Rodriguez, Maria H Van Zuilen, and Michael J Mintzer. An interactive e-learning tutorial for medical students on how to conduct the performance-oriented mobility assessment. Gerontology & geriatrics education, 28(1):51–60, 2007.

[195] Patricia Sánchez-González, Ignacio Oropesa, V Romero, A Fernández, A Albacete, E Asenjo, J Noguera, F Sánchez-Margallo, D Burgos, and Enrique J Gómez. Telma: technology enhanced learning environment for minimally invasive surgery. Procedia Computer Science, 3:316–321, 2011.

[196] Bjoern Sander and Mariola Monika Golas. Histoviewer: An interactive e-learning platform facilitating group and peer group learning. Anatomical sciences education, 6(3):182–190, 2013.

[197] Varun Saxena, Pradeep Natarajan, Patricia S O'Sullivan, and Sharad Jain. Effect of the use of instructional anatomy videos on student performance. Anatomical sciences education, 1(4):159–165, 2008.

[198] Katherine Schilling, John Wiecha, Deepika Polineni, and Souad Khalil. An interactive web-based curriculum on evidence-based medicine: Design and effectiveness. FAMILY MEDICINE-KANSAS CITY-, 38(2):126, 2006.

[199] C Schlorhaufer, M Behrends, G Diekhaus, M Keberle, and J Weidemann. Implementation of a web-based, interactive polytrauma tutorial in computed tomography for radiology residents: How we do it. European journal of radiology, 81(12):3942–3946, 2012.

[200] Andreas Schmeling, Manuel Kellinghaus, Jan Carl Becker, Ronald Schulz, Angelika Schafer, and Heidi Pfeiffer. A web-based e-learning programme for training external post-mortem examination in curricular medical education. International journal of legal medicine, 125(6):857–861, 2011.

[201] C Schmidt, M Reinehr, O Leucht, N Behrendt, S Geiler, and S Britsch. Mymicroscope—intelligent virtual microscopy in a blended learning model at ulm university. Annals of Anatomy-Anatomischer Anzeiger, 193(5):395–402, 2011.

[202] Daniel Schwarz, Petr Štourač, Martin Komenda, Hana Harazim, Martina Kosinová, Jakub Gregor, Richard Hlek, Olga Smékalová, Ivo Křikava, Roman Štoudek, et al. Interactive algorithms for teaching and learning acute medicine in the network of medical faculties mefanet. Journal of medical Internet research, 15(7), 2013.

[203] Kumar Seluakumaran, Felicita Fedelis Jusof, Rosnah Ismail, and Ruby Husain. Integrating an open-source course management system (moodle) into the teaching of a first-year medical physiology course: a case study. Advances in physiology education, 35(4):369–377, 2011.

[204] Francisco Sendra-Portero, Oscar E Torales-Chaparro, Miguel J Ruiz-Gómez, and Manuel Martínez-Morillo. A pilot study to evaluate the use of virtual lectures for undergraduate radiology teaching. European journal of radiology, 82(5):888–893, 2013.

[205] John Senga, Moses Ndiritu, Juliana Osundwa, Grace Irimu, and Mike English. Computer aided learning to link evidence to paediatric learning and practice: a pilot in a medical school in a low income setting. International health, 2(3):212–215, 2010.

[206] Kitt Shaffer and Juan E Small. Blended learning in medical education: Use of an integrated approach with web-based small group modules and didactic instruction for teaching radiologic anatomy 1. Academic radiology, 11(9):1059–1070, 2004.

[207] IM Shah, MR Walters, and JH McKillop. Acute medicine teaching in an undergraduate medical curriculum: a blended learning approach. Emergency Medicine Journal, 25(6):354–357, 2008.

[208] Timothy Shaw, Andrea Long, Sanjiv Chopra, and B Price Kerfoot. Impact on clinical behavior of face-to-face continuing medical education blended with online spaced education: A randomized controlled trial. Journal of Continuing Education in the Health Professions, 31(2):103–108, 2011.

[209] Fong-Ming Shyu, Ya-Fen Liang, WT Hsu, Jer-Junn Luh, and Heng-Shuen Chen. A problem-based e-learning prototype system for clinical medical education. Medinfo, 11(Pt 2):983–987, 2004.

[210] Charlotte Silén, Staffan Wirell, Joanna Kvist, Eva Nylander, and Orjan Smedby. Advanced 3d visualization in student-centred medical education. Medical teacher, 30(5):e115–e124, 2008.

[211] Cristiana Silveira Silva, Murilo Barreto Souza, Roberto Silveira Silva Filho, Luciana Molina de Medeiros, and Paulo Ricardo Criado. E-learning program for medical students in dermatology. Clinics, 66(4):619–622, 2011.

[212] Raymond A Smego Jr, Thomas A Herning, Lal Davis, Waheedah Hossain, and Saleh bin Mohammed Al-Khusaiby. A personal computer-based undergraduate medical school curriculum using sole. Teaching and learning in medicine, 21(1):38–44, 2009.

[213] Angela Smith, Max Maizels, Ruslan Korets, John S Wiener, Michael Stiener, Dennis B Liu, and Richard W Sutherland. A novel method of teaching surgical techniques to residents–computerized enhanced visual learning (cevl) with simulation to certify mastery of training: A model using newborn clamp circumcision. Journal of pediatric urology, 9(6):1210–1213, 2013.

[214] Josef Smolle, Gerhard Prause, and Freyja-Maria Smolle-Juttner. Emergency treatment of chest trauma—an e-learning simulation model for undergraduate medical students. European Journal of Cardio-Thoracic Surgery, 32(4):644–647, 2007.

[215] Gianvincenzo Sparacia, Floreana Cannizzaro, Donna M D'Alessandro, Michael P D'Alessandro, Giuseppe Caruso, and Roberto Lagalla. Initial experiences in radiology e-learning 1. Radiographics, 27(2):573–581, 2007.

[216] Axel Thomas Stadie, Ines Degenhardt, Gerardo Conesa, Robert Reisch, Ralf Alfons Kockro, Gerrit Fischer, and Heiko Hecht. Comparing expert and novice spatial representation on the basis of vr simula-tion, mri images, and physical objects. Journal of CyberTherapy & Rehabilitation, 3(3), 2010.

[217] Simon Stebbings, Nasser Bagheri, Kellie Perrie, Phil Blyth, and Jenny McDonald. Blended learning and curriculum renewal across three medical schools: The rheumatology module at the university of otago. Australasian Journal of Educational Technology, 28(7):1176–1189, 2012.

[218] Michael Steedman, Marwan Abouammoh, and Sanjay Sharma. Multimedia learning tools for teaching undergraduate ophthalmology: results of a randomized clinical study. Canadian Journal of Ophthalmology/Journal Canadien d'Ophtalmologie, 47(1):66–71, 2012.

[219] Nikolaos Stergiou, Giannis Georgoulakis, Niki Margari, Dionisios Aninos, Melina Stamataki, Efi Stergiou, Abraam Pouliakis, and Petros Karakitsos. Using a web-based system for the continuous distance education in cytopathology. International journal of medical informatics, 78(12):827–838, 2009.

[220] Alice Stewart, Garry Inglis, Luke Jardine, Pieter Koorts, and Mark William Davies. A randomised controlled trial of blended learning to improve the newborn examination skills of medical students. Archives of Disease in Childhood-Fetal and Neonatal Edition, 98(2):F141–F144, 2013.

[221] T Succar, G Zebington, F Billson, K Byth, S Barrie, P McCluskey, and J Grigg. The impact of the virtual ophthalmology clinic on medical students' learning: a randomised controlled trial. Eye, 27(10):1151–1157, 2013.

[222] Elena Svirko and Jane Mellanby. Attitudes to e-learning, learning style and achievement in learning neuroanatomy by medical students. Medical teacher, 30(9-10):e219–e227, 2008.

[223] Katherine A Sward, Stephanie Richardson, Jeremy Kendrick, and Chris Maloney. Use of a web-based game to teach pediatric content to medical students. Ambulatory Pediatrics, 8(6):354–359, 2008.

[224] S Tabakov, VC Roberts, B-A Jonsson, Michael Ljungberg, CA Lewis, Ronnie Wirestam, S-E Strand, I-L Lamm, F Milano, A Simmons, et al. Development of educational image databases and e-books for medical physics training. Medical engineering & physics, 27(7):591–598, 2005.

[225] Roland Talanow. Radiology teacher: a free, internet-based radiology teaching file server. Journal of the American College of Radiology, 6(12):871–875, 2009.

[226] S Tan, A Hu, T Wilson, H Ladak, P Haase, and K Fung. Role of a computer-generated three-dimensional laryngeal model in anatomy teaching for advanced learners. The Journal of Laryngology & Otology, 126(04):395–401, 2012.

[227] Sunčana Kukolja Taradi, Milan Taradi, Krešimir Radić, and Nikša Pokrajac. Blending problem-based learning with web technology positively impacts student learning outcomes in acid-base physiology. Advances in physiology education, 29(1):35–39, 2005.

[228] Hemal Thakore and Tim McMahon. An interactive e-tutorial in pathology. Medical education, 40(11):1135–1135, 2006.

[229] Hemal Thakore and Tim McMahon. Designing an interactive multimedia rich tutorial for medical students: Beyond a'book on a screen'. Journal of visual communication in medicine, 31(1):4–10, 2008.

[230] Natasha M Thomson, Dianne E Campbell, and Fenton M O'Leary. Teaching medical students to resuscitate children: An innovative two-part programme. Emergency Medicine Australasia, 23(6):741–747, 2011.

[231] Yukio Tsugihashi, Naoki Kakudate, Yoko Yokoyama, Yosuke Yamamoto, Hiroki Mishina, Norio Fukumori, Fumiaki Nakamura, Misa Takegami, Shinya Ohno, Takafumi Wakita, et al. A novel internet-based blended learning programme providing core competency in clinical research. Journal of evaluation in clinical practice, 19(2):250–255, 2013.

[232] Nigel Mcbeth Turner, Ria Scheffer, Eugene Custers, and Olle Th J Ten Cate. Use of unannounced spaced telephone testing to improve retention of knowledge after life-support courses. Medical teacher, 33(9):731–737, 2011.

[233] Tunde Varga-Atkins, Peter Dangerfield, and David Brigden. Developing professionalism through the use of wikis: A study with first-year undergraduate medical students. Medical teacher, 32(10):824–829, 2010.

[234] Joe Varghese, Minnie Faith, and Molly Jacob. Impact of e-resources on learning in biochemistry: first-year medical students' perceptions. BMC medical education, 12(1):21, 2012.

[235] Horst C Vollmar, Herbert Mayer, Thomas Ostermann, Martin E Butzlaff, John E Sandars, Stefan Wilm, and Monika A Rieger. Knowledge transfer for the management of dementia: a cluster randomised trial of blended learning in general practice. Implement Sci, 5(1):1, 2010.

[236] Minhong Wang, Bian Wu, Nian-Shing Chen, J Michael Spector, et al. Connecting problem-solving and knowledge-construction processes in a visualization-based learning environment. Computers & Education, 68:293–306, 2013.

[237] Travis P Webb and Taylor R Merkley. An evaluation of the success of a surgical resident learning portfolio. Journal of surgical education, 69(1):1–7, 2012.

[238] Verena Hézser-v Wehrs, Margarete Pfafflin, and Theodor W May. E-learning courses in epilepsy—concept, evaluation, and experience with the e-learning course “genetics of epilepsies”. Epilepsia, 48(5):872–879, 2007.

[239] John M Wiecha, VK Chetty, Timothy Pollard, and Peter F Shaw. Web-based versus face-to-face learning of diabetes management: the results of a comparative trial of educational methods. FAMILY MEDICINE-KANSAS CITY-, 38(9):647, 2006.

[240] John M Wiecha, Robert Gramling, Phyllis Joachim, and Hannelore Vanderschmidt. Collaborative e-learning using streaming video and asynchronous discussion boards to teach the cognitive foundation of medical interviewing: a case study. Journal of Medical Internet Research, 5(2), 2003.

[241] Reto A Wildhaber, François Verrey, and Roland H Wenger. A graphical simulation software for instruction in cardiovascular mechanics physiology. Biomedical engineering online, 10(1):8, 2011.

[242] AS Wilson, JE Goodall, G Ambrosini, DM Carruthers, H Chan, SG Ong, C Gordon, and SP Young. Development of an interactive learning tool for teaching rheumatology—a simulated clinical case studies program. Rheumatology, 45(9):1158–1161, 2006.

[243] Vanessa Woltering, Andreas Herrler, Klaus Spitzer, and Cord Spreckelsen. Blended learning positively affects students’ satisfaction and the role of the tutor in the problem-based learning process: results of a mixed-method evaluation. Advances in Health Sciences Education, 14(5):725–738, 2009.

[244] Charles R Woods and Kathi J Kemper. Curriculum resource use and relationships with educational outcomes in an online curriculum. Academic Medicine, 84(9):1250–1258, 2009.

[245] Bjarne Skjodt Worm and Kenneth Jensen. Does peer learning or higher levels of e-learning improve learning abilities? a randomized controlled trial. Medical education online, 18, 2013.

[246] Ting Wu, Andreas Zimolong, Norbert Schiffers, and Klaus Radermacher. A software framework for the development of web-based medical education using learning object classes. Informatics for Health and Social Care, 31(1):9–22, 2006.

[247] Markus Wunschel, Ulf Leichtle, Nikolaus Wulker, and Torsten Kluba. Using a web-based orthopaedic clinic in the curricular teaching of a german university hospital: analysis of learning effect, student usage and reception. international journal of medical informatics, 79(10):716–721, 2010.

[248] G Yang and CC Tchoyoson Lim. Singapore national medical image resource centre (sn. mirc): a world wide web resource for radiology education. ANNALS-ACADEMY OF MEDICINE SINGAPORE, 35(8):558, 2006.

[249] Rachel L Yang, Daniel A Hashimoto, Jarrod D Predina, Nina M Bowens, Elizabeth M Sonnenberg, Emily C Cleveland, Charlotte Lawson, Jon B Morris, and Rachel R Kelz. The virtual-patient pilot: testing a new tool for undergraduate surgical education and assessment. Journal of surgical education, 70(3):394–401, 2013.

[250] Leanne M Yanni, John W Priestley, Jeanne B Schlesinger, Jessica M Ketchum, Betty A Johnson, and Sarah E Harrington. Development of a comprehensive e-learning resource in pain management. Pain Medicine, 10(1):95–105, 2009.

[251] JEW Zajaczek, F Gotz, T Kupka, M Behrends, B Haubitz, F Donnerstag, T Rodt, GF Walter, HK Matthies, and H Becker. elearning in education and advanced training in neuroradiology: introduction of a web-based teaching and learning application. Neuroradiology, 48(9):640–646, 2006.
