# Supplementary material for: What Are We Looking for in Computer-Based Learning Interventions in Medical Education? A Systematic Review
Source: J Med Internet Res. 2016 Aug 1;18(8):e204. doi: 10.2196/jmir.5461 (PMC4985611; doi:10.2196/jmir.5461)
Supplement: Multimedia Appendix 4 [file jmir_v18i8e204_app4.zip › dist/index.html]

       Visualization   

## About this visualization Dismiss

Use this visualization to explore the articles included in the review.

### Variable menu on the left side of the screen

Use this menu to pick the variables of interest.

Remove a variable from the pivot table.

Add a variable to the pivot table rows.

Add a variable to the pivot table columns.

Set the graph node labels to the values on this variable.

Partition nodes on the graph according to the values in this variable.

Show / hide variable values. Pick those to filter the papers included in the pivot table and in the graph.

### Graph in the background

Explore the relationships between papers.

Each node represents a paper included in this review.

Nodes are connected if they cite each other or if they have citation or references in common.

The connection line is broader for papers that share more citations or references.

#### Navigation

pan Click and hold on empty space.

zoom Scroll on empty space.

click Highlight a paper node and its connections.

### Actions menu on the bottom of the screen

Resets the filters and removes all columns and rows from the pivot table.

Shows / hides the menus.

Shows / hides connections between the nodes in the graph.

Shows / hides node labels.

Built using open source libraries (Angular, D3, Semantic-UI, jQuery, jQuery-ui, pivottable and Gulp).

Dismiss

  

**A systematic review on computer based learning interventions in medical education - what are we looking for?**  An interactive visualization

#### {{::key }}

{{::attr.label}}                       

{{::cat}}

#### {{selected.Title}}

Pubmed  Google Scholar

| {{::key }} | |
| --- | --- |
| {{attr.label}} | {{selected[attr.key]}} |

   
